# A splice site variant in *MADD* affects hormone expression in pancreatic **β** cells and pituitary gonadotropes

**DOI:** 10.1172/jci.insight.167598

**Published:** 2024-05-22

**Authors:** Kristiina Pulli, Jonna Saarimäki-Vire, Pekka Ahonen, Xiaonan Liu, Hazem Ibrahim, Vikash Chandra, Alice Santambrogio, Yafei Wang, Kirsi Vaaralahti, Anna-Pauliina Iivonen, Johanna Känsäkoski, Johanna Tommiska, Yasmine Kemkem, Markku Varjosalo, Sanna Vuoristo, Cynthia L. Andoniadou, Timo Otonkoski, Taneli Raivio

**Affiliations:** 1 Stem Cells and Metabolism Research Program (STEMM), Research Programs Unit, Faculty of Medicine, and; 2 Institute of Biotechnology, Helsinki Institute of Life Science (HiLIFE), University of Helsinki, Helsinki, Finland.; 3 Centre for Craniofacial and Regenerative Biology, King’s College London, London, United Kingdom.; 4 Department of Medicine III, University Hospital Carl Gustav Carus, Technische Universität Dresden, Dresden, Germany.; 5 Department of Physiology, Faculty of Medicine;; 6 Department of Obstetrics and Gynecology; and; 7 HiLIFE, University of Helsinki, Helsinki, Finland.; 8 New Children’s Hospital, Helsinki University Hospital, Pediatric Research Center, Helsinki, Finland.

**Keywords:** Endocrinology, Reproductive biology, Beta cells, Genetic diseases, Neuroendocrine regulation

## Abstract

MAPK activating death domain (MADD) is a multifunctional protein regulating small GTPases RAB3 and RAB27, MAPK signaling, and cell survival. Polymorphisms in the *MADD* locus are associated with glycemic traits, but patients with biallelic variants in *MADD* manifest a complex syndrome affecting nervous, endocrine, exocrine, and hematological systems. We identified a homozygous splice site variant in *MADD* in 2 siblings with developmental delay, diabetes, congenital hypogonadotropic hypogonadism, and growth hormone deficiency. This variant led to skipping of exon 30 and in-frame deletion of 36 amino acids. To elucidate how this mutation causes pleiotropic endocrine phenotypes, we generated relevant cellular models with deletion of *MADD* exon 30 (*dex30*). We observed reduced numbers of β cells, decreased insulin content, and increased proinsulin-to-insulin ratio in *dex30* human embryonic stem cell–derived pancreatic islets. Concordantly, *dex30* led to decreased insulin expression in human β cell line EndoC-βH1. Furthermore, *dex30* resulted in decreased luteinizing hormone expression in mouse pituitary gonadotrope cell line LβT2 but did not affect ontogeny of stem cell–derived GnRH neurons. Protein-protein interactions of wild-type and *dex30* *MADD* revealed changes affecting multiple signaling pathways, while the GDP/GTP exchange activity of *dex30* *MADD* remained intact. Our results suggest *MADD*-specific processes regulate hormone expression in pancreatic β cells and pituitary gonadotropes.

## Abstract 2

A splice variant of MADD that skips exon 30 causes diabetes and hypopituitarism and affects insulin and luteinizing hormone production a mouse model.

## Graphical abstract

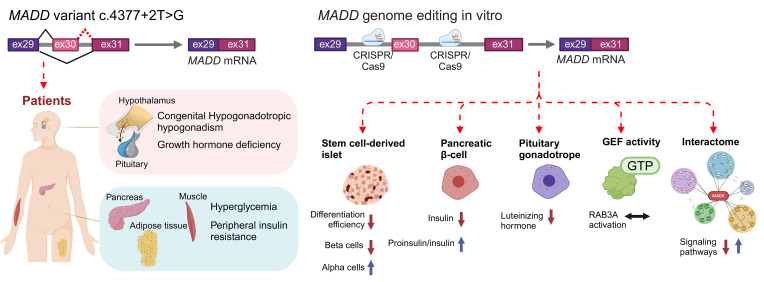

## Introduction

MAPK activating death domain (MADD) is a ubiquitously expressed protein (1) regulating activation of secretory small GTPases RAB3 and RAB27 (2–4). MADD has been implicated in exocytosis (4–10) and linking endocytic and secretory pathways (11). MADD regulates transportation of synaptic vesicles (10, 12) and storage granules (3, 4, 13), possibly by a mechanism involving localization of activated RAB3/RAB27 (3, 4, 13) and protein-protein interactions (12). MADD has an N-terminal DENN domain and C-terminal serine-rich and death domains. DENN domain is a common component of Rab GDP/GTP exchange factors (GEFs) (14), and it directly binds to target Rabs (12). Intact death domain is likewise important for the GEF activity of MADD (13, 15), as well as for protein-protein interactions (12, 16). Interaction of death domains of MADD and TNF receptor 1 (TNFR1) leads to activation of TNF-α–stimulated ERK1/2 phosphorylation and protection from apoptosis (16–19). Indeed, high expression of MADD appears to protect cells from apoptosis (17, 20–22), though this effect may be splice variant dependent (1, 20, 23, 24).

Polymorphisms in *MADD* locus are associated with fasting glucose and proinsulin levels in genome- and exome-wide studies (25–34). Conditional knockout of *Madd* in mouse β cells leads to hyperglycemia, glucose intolerance, and reduced and delayed glucose-induced insulin release (35), and rhythmic alternative splicing of *Madd* transcript regulates glucose-stimulated insulin secretion in mouse β cells (36). However, biallelic *MADD* variants cause a complex phenotype ranging from mild developmental delay to severe multisystem disease affecting nervous, endocrine, exocrine, and hematological systems (37–39). The majority of pathogenic *MADD* variants affect the conserved protein domains, but there is no clear genotype-phenotype correlation. Most of the patients have hypopituitarism, with neonatal growth hormone deficiency (GHD) and low follicle-stimulating hormone (FSH) level being the most common findings (37–39). Patient-derived fibroblasts display decreased *MADD* expression, reduced TNF-α–stimulated ERK1/2 phosphorylation, increased susceptibility to cell death, and reduced endocytosis of EGF (37).

Here, we extend these observations by describing a homozygous exon 30–skipping variant in *MADD* leading to in-frame deletion of 36 amino acids close to the C-terminal death domain. Homozygous carriers of the exon 30–skipping variant had hyperglycemia, insulin resistance, congenital hypogonadotropic hypogonadism (CHH), hypopituitarism, and developmental delay. To model the endocrine disease, we deleted *MADD* exon 30 (*dex30*) from the genomic DNA of relevant cell types in vitro. We discovered reduced differentiation efficiency of stem cell–derived *dex30* pancreatic progenitors and deficient insulin content and processing in *dex30* pancreatic β cells. While *dex30* did not affect the ontogeny of stem cell–derived gonadotropin-releasing hormone (GnRH) neurons, we observed reduced luteinizing hormone (LH) expression in *dex30* gonadotropes. Surprisingly, *dex30* did not cause crude secretory defects or reduced activation of RAB3A, suggesting that MADD may regulate hormone content via mechanisms independent of its GEF activity and that these functions are disrupted by *dex30*. Concordantly, *dex30* led to changes in the protein-protein interactions of MADD.

## Results

### Clinical description of the patients

**Figure 1:**
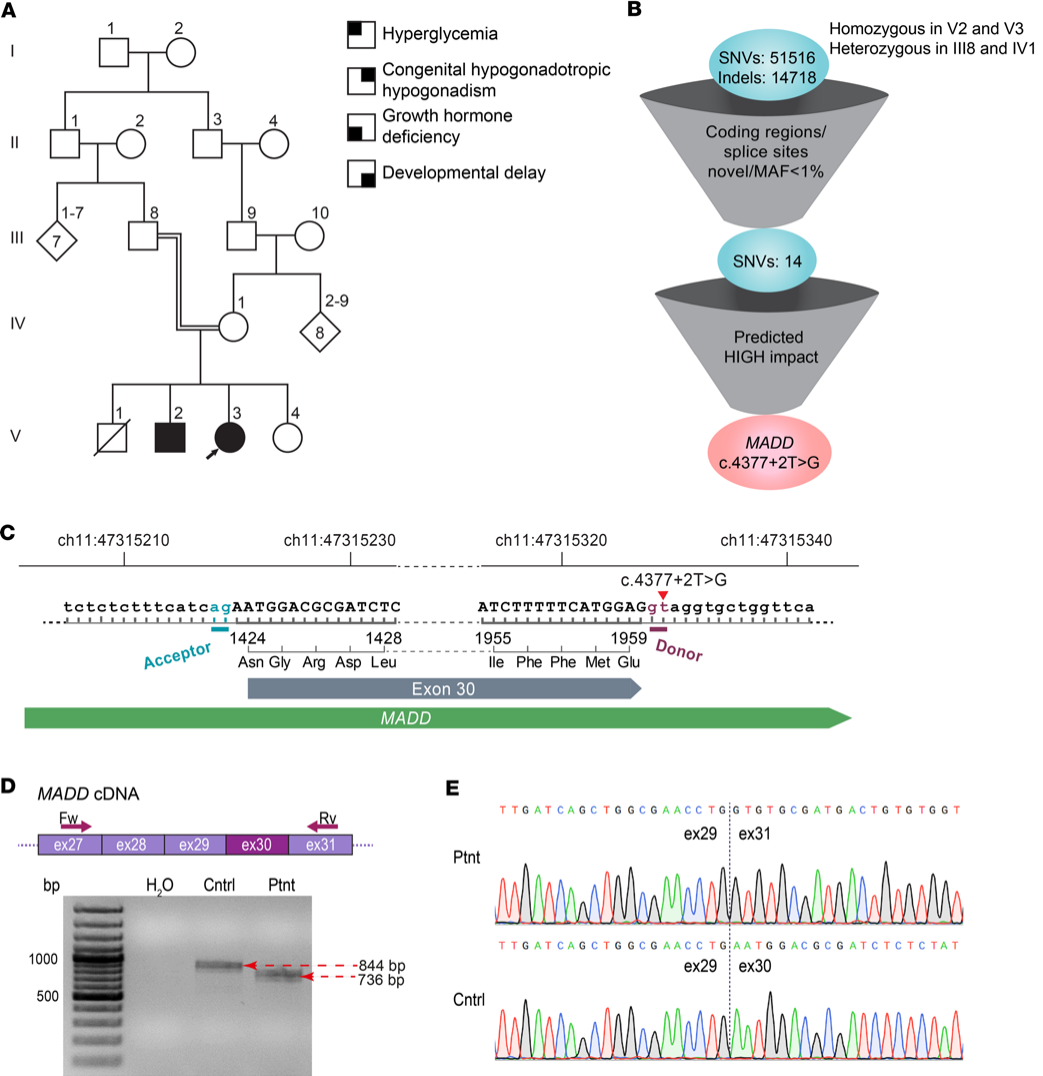
Discovering a splice site variant in *MADD*. (**A**) Consanguineous family with 2 siblings affected by a complex syndrome. Index patient is marked with an arrow. (**B**) Filtering of the single nucleotide variants (SNVs) in the affected siblings and their parents. MAF, minor allele frequency. (**C**) A schematic showing the c.4377+2T>G variant in *MADD* affecting the consensus splice donor site and leading to in-frame deletion of 36 amino acids (NCBI Reference Sequence: NP_003673.3: p.Asn1424_Glu1959del). (**D**) RT-PCR amplification of *MADD* transcript around exon 30. (**E**) Sequencing chromatograms of the PCR products from **D**. Cntrl, a healthy control individual; Ptnt, index patient.

The consanguineous family with 2 affected siblings is depicted in Figure 1A. Clinical measurements are in Supplemental Data 1; supplemental material available online with this article; https://doi.org/10.1172/jci.insight.167598DS1

#### Patient 1.

The index patient (individual V3 in Figure 1A) had a developmental delay, and at the age of 14 years, elevated level of glycated hemoglobin (HbA1c) was observed. The oral glucose tolerance test revealed elevated fasting plasma glucose and glucose intolerance. Insulin, C-peptide, and markers of β cell autoimmunity were within normal range, suggesting insulin resistance. The patient was prepubertal and had low estradiol and LH levels and minimal LH response to GnRH stimulation, suggesting gonadotropin deficiency. Her serum insulin-like growth factor 1 (IGF-1) was low for age, and low growth hormone (GH) response to arginine was suggestive of GHD. She was treated with metformin and estrogen but not with GH. At the age of 19 years her puberty was incomplete despite estrogen therapy, consistent with CHH. Her GH response to arginine stimulation remained low, confirming GHD.

#### Patient 2.

Patient 2 (individual V2 in Figure 1A) had severe developmental delay and epilepsy. Elevated fasting plasma glucose and HbA1c were first observed at the age of 21 years. Patient 2 had prepubertal levels of testosterone and LH, and no physical signs of puberty, indicating absent puberty due to CHH. Patient 2’s IGF-1 level was low, suggesting GHD. At the age of 28 years, the patient developed polyuria due to hyperglycemia and was treated with metformin.

The family includes 2 other children, of which the firstborn (individual V1 in Figure 1A) died of asphyxia caused by strangulation of the umbilical cord during the delivery, and the youngest (individual V4 in Figure 1A) is developing normally at the age of 5 years.

### A splice site variant in MADD leads to skipping of exon 30

To reveal the underlying mutation, we performed whole-genome sequencing (WGS) of the affected siblings and their parents at the Beijing Genomic Institute (BGI). WGS data had an average sequencing depth of 34.27 reads and coverage of at least 99.09% for each sample. Due to the consanguinity of the parents, a recessive homozygous mutation was considered the most likely cause of the phenotype. The WGS data were first filtered to include SNVs or insertions/deletions homozygous in both affected patients and heterozygous in their parents, then to include only variants in the coding regions or splice sites with a frequency of less than 1% in the 1000 Genomes database (40) (Figure 1B).

After the WGS data filtering, 14 SNVs (8 synonymous, 5 missense, and 1 splice site) were left (Figure 1B and Supplemental Table 1). According to predictions from the BGI, only a splice site variant in *MADD* had a high impact. This variant, c.4377+2T>G at National Center for Biotechnology (NCBI) Reference Sequence NM_003682.4, affects the canonical consensus splice donor site of exon 30 (Figure 1C) and has not been reported in the gnomAD, dbSNP, and Ensembl databases. Human Splicing Finder (41) predicted an alteration that most probably affects splicing, and this prediction was corroborated by SpliceAI/Pangolin (42), NNSplice (43), HAL Splice Prediction (44), and Spliceator (45) (Supplemental Table 2). Reverse transcriptase PCR (RT-PCR) and Sanger sequencing validated complete skipping of *MADD* exon 30 in the RNA obtained from the index patient (Figure 1, D and E). The defective splicing of exon 30 in the c.4377+2T>G variant was further validated by a minigene assay (Supplemental Figure 1). At the protein level, skipping of exon 30 leads to in-frame deletion of 36 amino acids in the C-terminal part of the MADD protein, 8 amino acids downstream from the conserved death domain (Supplemental Figure 2).

We further assessed the predicted impacts of the 5 additional missense variants with SIFT (46) and PolyPhen2 (47) web tools. Two of the variants, p.(Arg544Cys) in 1-aminocyclopropane-1-carboxylate synthase homolog (inactive) like, encoded by *ACCSL*, and p.(Arg78Cys) in tetraspanin 18, encoded by *TSPAN18*, were predicted to be deleterious/damaging by both tools (Supplemental Table 1). However, *ACCSL* and *TSPAN18* have not been implicated in neurological or endocrine phenotypes, and given that biallelic mutations in *MADD* have been previously linked with developmental delay and hypopituitarism (37, 38) (Supplemental Figure 2), we were confident that the causative variant in the probands was the splice site mutation in *MADD*.

### MADD regulates insulin expression in immortalized human pancreatic β cells

**Figure 2:**
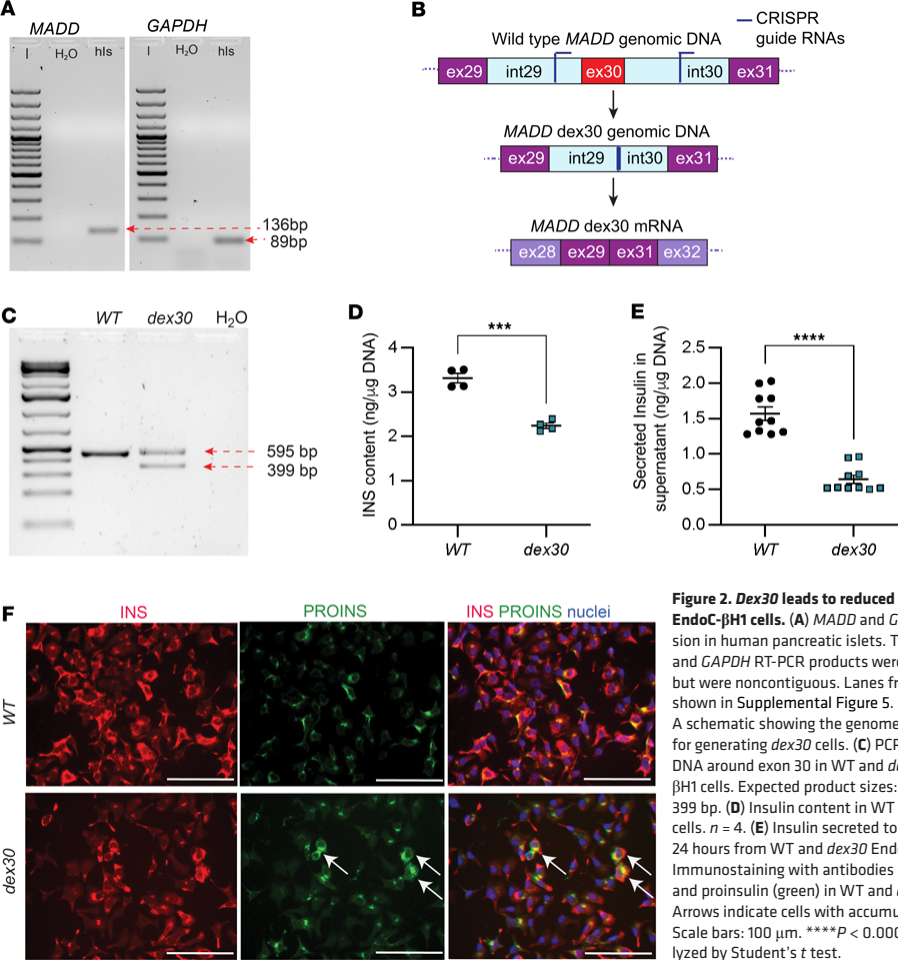
*Dex30* leads to reduced insulin expression in EndoC-βH1 cells. (**A**) *MADD* and *GAPDH* mRNA expression in human pancreatic islets. The lanes with *MADD* and *GAPDH* RT-PCR products were run on the same gel but were noncontiguous. Lanes from the same gel are shown in Supplemental Figure 5. hIs, human islets. (**B**) A schematic showing the genome-editing strategy used for generating *dex30* cells. (**C**) PCR with *MADD* genomic DNA around exon 30 in WT and *dex30*-enriched EndoC-βH1 cells. Expected product sizes: WT 595 bp, *dex30* 399 bp. (**D**) Insulin content in WT and *dex30* EndoC-βH1 cells. *n* = 4. (**E**) Insulin secreted to culture media during 24 hours from WT and *dex30* EndoC-βH1 cells. *n* = 10. (**F**) Immunostaining with antibodies specific to insulin (red) and proinsulin (green) in WT and *dex30* EndoC-βH1 cells. Arrows indicate cells with accumulation of proinsulin. Scale bars: 100 μm. ****P < 0.0001, ***P < 0.001, analyzed by Student’s *t* test.

To elucidate the role of *MADD* in human β cells, we verified robust expression of *MADD* in cadaveric human islet cDNA by RT-PCR (Figure 2A). We proceeded to silence *MADD* expression in human immortalized β cell line EndoC-βH1 (48) using siRNAs (si*MADD*) that resulted in over 90% reduction of *MADD* mRNA expression (Supplemental Figure 3A). We observed a significant reduction of insulin (*INS*) and pre*INS* mRNA levels in si*MADD*-treated cells compared with cells treated with scrambled siRNAs (siNT, Supplemental Figure 3, B and C). Silencing of *MADD* expression did not affect mRNA levels of *PDX1* and *MAFA* (Supplemental Figure 3, D and E), indicating that *MADD* regulates insulin expression independently from these transcription factors in EndoC-βH1 cells. Reduction of *INS* mRNA was reflected by decreased insulin content and glucose-stimulated insulin secretion in the si*MADD*-treated EndoC-βH1 cells (Supplemental Figure 3, F and G). However, the percentage secretion relative to the total insulin content was not affected, indicating that there were no crude defects in the secretion machinery (Supplemental Figure 3H). In addition, the proinsulin/C-peptide ratio was comparable between si*MADD* and siNT cells (Supplemental Figure 3I). These results suggest that *MADD* regulates insulin expression in EndoC-βH1 cells.

### Dex30 leads to decreased insulin expression in immortalized human pancreatic β cells

To investigate the effect of skipping of *MADD* exon 30 on insulin expression in EndoC-βH1 cells, we directed CRISPR/Cas9-GFP by 2 guide RNAs that were designed to target the intronic sites around *MADD* exon 30 (Figure 2B). The genome-edited *dex30* cells were enriched by FACS and validated by PCR (Figure 2C). Again, we observed decreased pre*INS* and *INS* mRNA levels in *dex30* EndoC-βH1 cells (Supplemental Figure 3, J and K), while mRNA levels of *PDX1* and *MADD* remained unaltered (Supplemental Figure 3, L and M). Insulin content and basal insulin secretion into the culture media were significantly reduced in *dex30* EndoC-βH1 cells when normalized to total DNA (Figure 2, D and E). However, the basal and glucose-stimulated insulin secretion in *dex30* cells was comparable to wild-types when normalized to the total insulin content, suggesting that the secretion machinery remained functional (Supplemental Figure 3N). In addition, immunostaining suggested accumulation of proinsulin in some of the *dex30* EndoC-βH1 cells (Figure 2F). In summary, our results indicate that *dex30* affects insulin expression in EndoC-βH1 cells.

### Dex30 in human embryonic stem cells reduces their differentiation efficiency into stem cell–derived pancreatic islets and affects insulin processing

**Figure 3:**
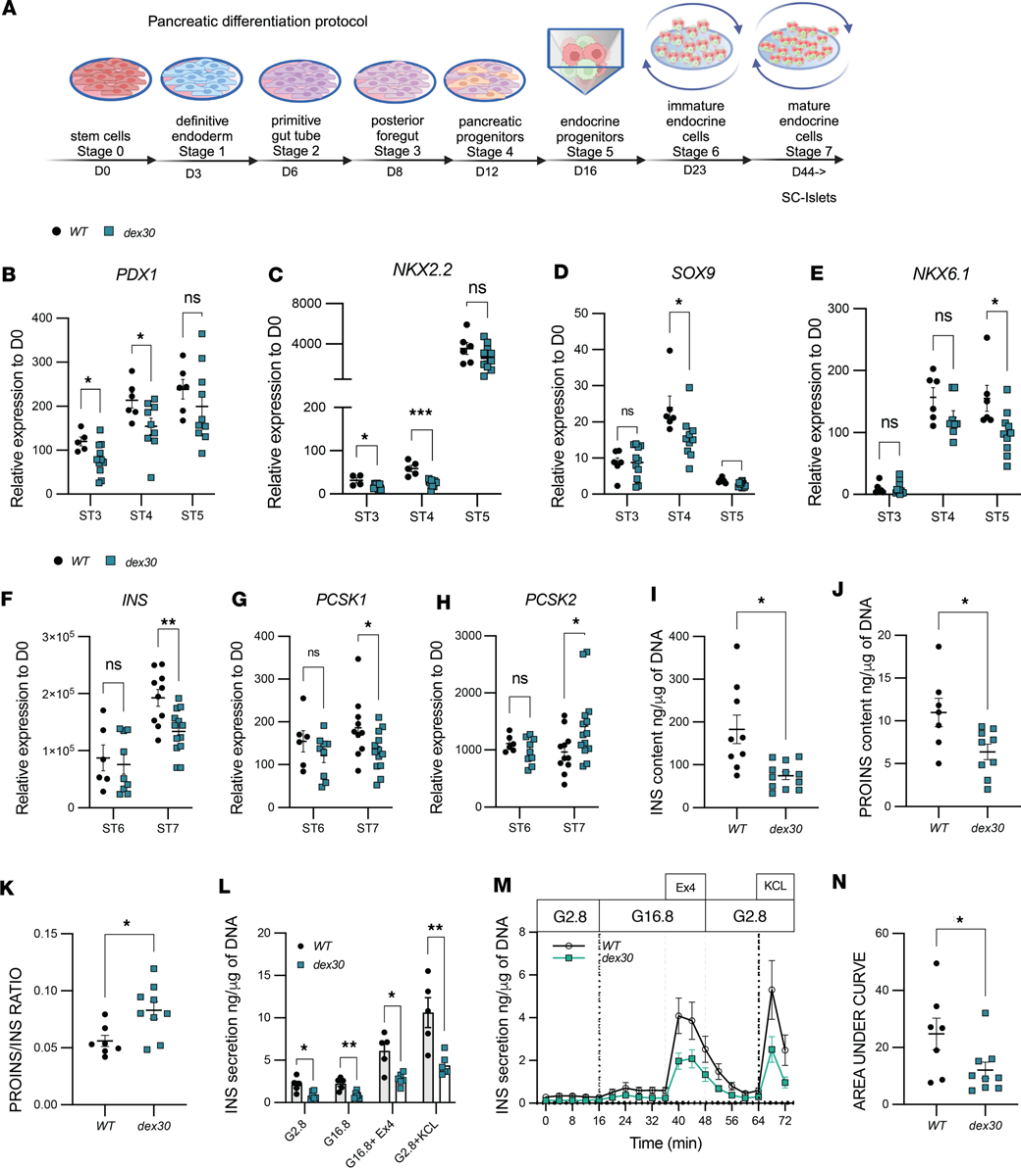
*Dex30* leads to reduced number of β cells, lowered insulin content, and impaired insulin processing in SC-islets. (**A**) Schematic of SC-islet differentiation protocol. (**B**) *PDX1*, (**C**) *NKX2-2*, (**D**) *SOX9*, and (**E**) *NKX6-1* mRNA levels in WT and *dex30* cultures at stages 3–5, relative to undifferentiated stem cells (*n* = 4–6 for WT, *n* = 8–11 for *dex30*). (**F**) *INS*, (**G**) *PCSK1*, and (**H**) *PCSK2* mRNA levels in WT and *dex30* cultures at stages 6–7, relative to undifferentiated stem cells (*n* = 6 for WT, *n* = 9 for *dex30* at ST6, *n* = 10 for WT, and *n* = 13–14 for *dex30* at stage 7). (**I**) Total insulin and (**J**) proinsulin content in WT and *dex30* SC-islets at stage 7 (*n* = 7–9 for WT, *n* = 9–12 for *dex30*). (**K**) Proinsulin-to-insulin ratio in WT and *dex30* SC-islets at stage 7 (*n* = 7 for WT, *n* = 9 for *dex30*). (**L**) Static insulin secretion from WT and *dex30* SC-islets at stage 7, in 2.8 mM glucose (G2.8), 16.8 mM glucose (G16.8), 16.8 mM glucose + 50 mM Exendin 4 (Ex4), and 2.8 mM glucose with 30 mM KCl (*n* = 5 for WT, *n* = 6 for *dex30*). (**M**) Dynamic insulin secretion in WT and *dex30* SC-islets at stage 7. Conditions like in **L** (*n* = 7 for WT, *n* = 9 for *dex30*). (**N**) Area under curve quantification of dynamic insulin secretion in **M**. ***P < 0.001, **P < 0.01, and *P < 0.05 analyzed by Student’s *t* test (**I**–**K** and **N**) or multiple *t* tests (**B**–**H** and **L**).

To explore the effects of *dex30* on development of pancreatic β cells, we used the CRISPR/Cas9 strategy described for EndoC-βH1 cells in human embryonic stem cell (hESC) line H9 (49). Genome-edited cells were single-cell–sorted to generate clonal populations. We identified 2 homozygous *dex30* clones (Supplemental Figure 4A) that were differentiated into stem cell–derived pancreatic islets (SC-islets) alongside isogenic controls following a 7-stage differentiation protocol (Figure 3A) (50). The *dex30* cells differentiated into stage 1 definitive endoderm comparably to wild-types (Supplemental Figure 4B). At stage 4, we observed a reduced number of NKX6-1^+^ pancreatic progenitors (Supplemental Figure 4C), indicating lower differentiation efficiency. The mRNA levels of *MADD* and *NGN3* were comparable between the *dex30* and the wild-type cells at stages 3–5 (Supplemental Figure 4, D and E). However, decreased mRNA levels of *PDX1* and *NKX2-2* at stages 3 and 4, *SOX9* at stages 4 and 5, and *NKX6-1* at stage 5 indicated that differentiation to pancreatic progenitors was less efficient in *dex30* cultures, especially at stages 4 and 5 (Figure 3, B–E). This was reflected by lower number of C-PEP^+^NKX6-1^+^ *dex30* β cells at stage 7 (Supplemental Figure 4F). The total number of endocrine cells in *dex30* SC-islets was comparable to wild-types by flow cytometry and immunohistochemistry (Supplemental Figure 4, F–H), but *dex30* SC-islets had significantly higher numbers of glucagon-positive α cells concomitant with a trend of reduced insulin-positive β cells (Supplemental Figure 4, G and H). Concordantly, *INS* mRNA expression was reduced at stage 7 (Figure 3F). *MADD* mRNA level in *dex30* SC-islets was comparable to wild-types at stage 6 and mildly increased at stage 7 (Supplemental Figure 4I), verifying that *dex30* does not lead to degradation of *MADD* mRNA.

During insulin biosynthesis, proinsulin is transported through the Golgi network to secretory granules, in which proprotein convertase 1/3 encoded by *PCSK1* and exopeptidase carboxypeptidase E/H encoded by *CPE* remove the C-peptide, leading to the formation of mature insulin (51). In humans *PCSK1* is specifically expressed in β cells, whereas proprotein convertase 2 encoded by *PCSK2* is specific to glucagon-producing α cells (52) To study insulin processing in the *dex30* SC-islets, we quantitated the mRNA expression of *PCSK1*, *PCSK2*, and *CPE* during stages 6–7. The mRNA level of *PCSK1* was significantly decreased, while the mRNA level of *PCSK2* was significantly increased in *dex30* SC-islets at stage 7 (Figure 3, G and H), likely reflecting the increased proportion of α cells. The mRNA level of *CPE* was not altered in *dex30* SC-islets (Supplemental Figure 4J).

Concordant with the reduced insulin mRNA expression, insulin content was reduced in *dex30* SC-islets when normalized to total DNA (Figure 3I) or to β cell DNA (Supplemental Figure 4M), suggesting that insulin content was lower in individual *dex30* β cells. Interestingly, the proinsulin content was reduced to a lesser extent, leading to significantly increased proinsulin-to-insulin ratio in *dex30* SC-islets (Figure 3, J and K), but immunohistochemistry revealed no clear changes in the proportions of proinsulin^+^, proinsulin^+^insulin^+^, and insulin^+^ cells (Supplemental Figure 4, K and L). These results suggest that insulin processing was compromised in *dex30* β cells.

To assess the functionality of *dex30* β cells, we analyzed insulin secretion in the SC-islets (Figure 3, L–N, and Supplemental Figure 4, N–P). We performed sequential incubations with low glucose (2.8 mM), high glucose (16.8 mM), high glucose with 50 nM glucagon like peptide-1 receptor agonist Ex4 (potentiates glucose-stimulated insulin secretion), and low glucose with 30 mM KCl (induces exocytosis of remaining insulin granules). In each tested condition, the *dex30* SC-islets secreted significantly less insulin compared with wild-types when normalized to total DNA (Figure 3L). We studied the dynamics of insulin secretion using a perifusion assay with the same stimuli (Figure 3M). AUC quantifications verified the reduced insulin secretion in the *dex30* SC-islets (Figure 3N). However, the relative responses, measured as the stimulation index (Supplemental Figure 4N) or insulin secretion normalized to total insulin content (Supplemental Figure 4, O and P), were not affected. These results indicate that the absolute insulin secretion in the *dex30* SC-islets is reduced, but the secretory machinery remains intact. We did not observe increased apoptosis in *dex30* SC-islets (Supplemental Figure 4, Q and R). In summary, the *dex30* hESCs differentiated into SC-islets less efficiently than the wild-type cells, and their insulin content and secretion were markedly reduced, possibly reflecting defects in insulin processing as suggested by increased proinsulin-to-insulin ratio.

### MADD is expressed in human and mouse hypothalamic neurons, but dex30 does not compromise hESCs’ potential to differentiate into GnRH-expressing neurons

**Figure 4:**
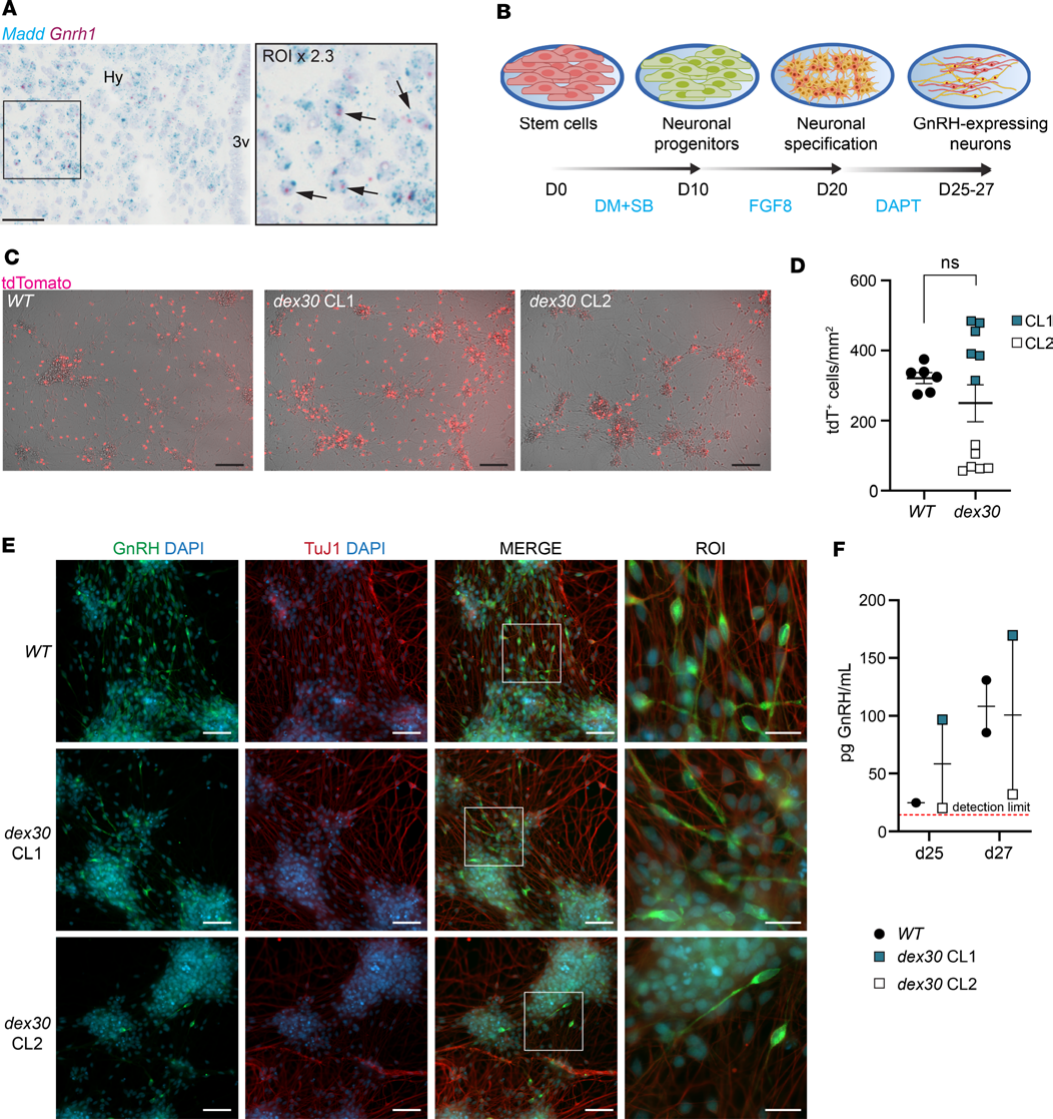
*Dex30* hESCs can differentiate to GnRH-expressing neurons. (**A**) A representative RNAscope RNA in situ hybridization using probes against *Madd* (blue) and *Gnrh1* (red) in adult (P45) mouse hypothalamus, coronal section. Experiments were repeated >10 times. Arrows indicate examples of double-positive cells. Scale bar: 50 μm. 3v, 3rd ventricle; Hy, hypothalamus. (**B**) A schematic of the hESC-derived GnRH-expressing neuron differentiation protocol. DM, dorsomorphin; SB, SB431542; FGF8, fibroblast growth factor 8; DAPT, gamma-secretase inhibitor IX. (**C**) tdTomato fluorescent reporter indicating expression of *GNRH1* in WT and *dex30* cultures on day 27. Scale bars: 100 μm. (**D**) The number of tdTomato^+^ cells on day 27 (*n* = 6 for WT, *n* = 12 for *dex30*). (**E**) Immunocytochemistry with antibodies against GnRH (green) and neuron-specific class II β-tubulin TuJ1 (red) in WT and *dex30* cultures on day 27. Scale bars: 50 μm, region of interest (ROI) scale bar: 20 μm. (**F**) Secretion of GnRH to culture medium during 48 hours from WT and *dex30* GnRH-expressing cells on days 25 and 27 (*n* = 1–2). NS *P* ≥ 0.05, analyzed by Student’s *t* test. CL, clone.

Hypothalamic GnRH neurons regulate the onset of puberty by secreting GnRH and activating pituitary gonadotropes to release LH and FSH, which in turn regulate gonadal estrogen and testosterone production (53). The patients with *MADD* exon 30–skipping variant manifested absent puberty and low levels of LH, estrogen, and testosterone, which could originate from either hypothalamic or pituitary defects. To elucidate the hypothalamic component, we confirmed robust *MADD* mRNA expression by RT-PCR in a human hypothalamus cDNA library (Supplemental Figure 5A). To explore the coexpression of *Madd* and *Gnrh1*, we performed RNAscope mRNA in situ hybridization in mouse hypothalamus and observed ubiquitous expression of *Madd*, including in the *Gnrh1*-expressing cells (Figure 4A). A negative control demonstrating absence of *Gnrh1* signal in a caudal section is shown in Supplemental Figure 5B, and negative and positive probe controls are shown in Supplemental Figure 5C. Concordantly, single-cell RNA-Seq data from mouse hypothalamus (54) revealed the highest expression of *Madd* in the cluster of GABAergic neurons (Supplemental Figure 5D), including GnRH neurons (55, 56). With relevance to the GHD in the proband, we observed coexpression of *Madd* and GH-releasing hormone (*Ghrh*) transcripts (Supplemental Figure 5E).

To explore the impact of deleting *MADD* exon 30 on the development of GnRH neurons, we differentiated the *dex30* hESC clones into SC-derived GnRH neurons according to our established protocol (Figure 4B) (49, 55, 57, 58). The hESCs used in this study carry a *GNRH1-tdTomato* reporter gene allowing visualization of *GNRH1*-expressing cells (49). We detected tdTomato^+^ cells (Figure 4, C and D) and GnRH-immunopositive and neuron-specific class III β-tubulin–immunopositive (TuJ1-immunopositive) neurons in both wild-type and *dex30* cultures on differentiation day 27 (Figure 4E), verifying that *dex30* hESCs were able to differentiate to GnRH-expressing neurons. Additionally, *dex30* cells secreted GnRH decapeptide to the culture medium (Figure 4F). The variability in the differentiation efficiency is consistent with previous observations in wild-type cultures (57, 58). In summary, *dex30* hESCs were able to differentiate to GnRH-secreting neurons.

### MADD is expressed in pituitary hormone–producing cells, and dex30 pituitary gonadotropes display reduced LH expression while retaining their responsiveness to GnRH

**Figure 5:**
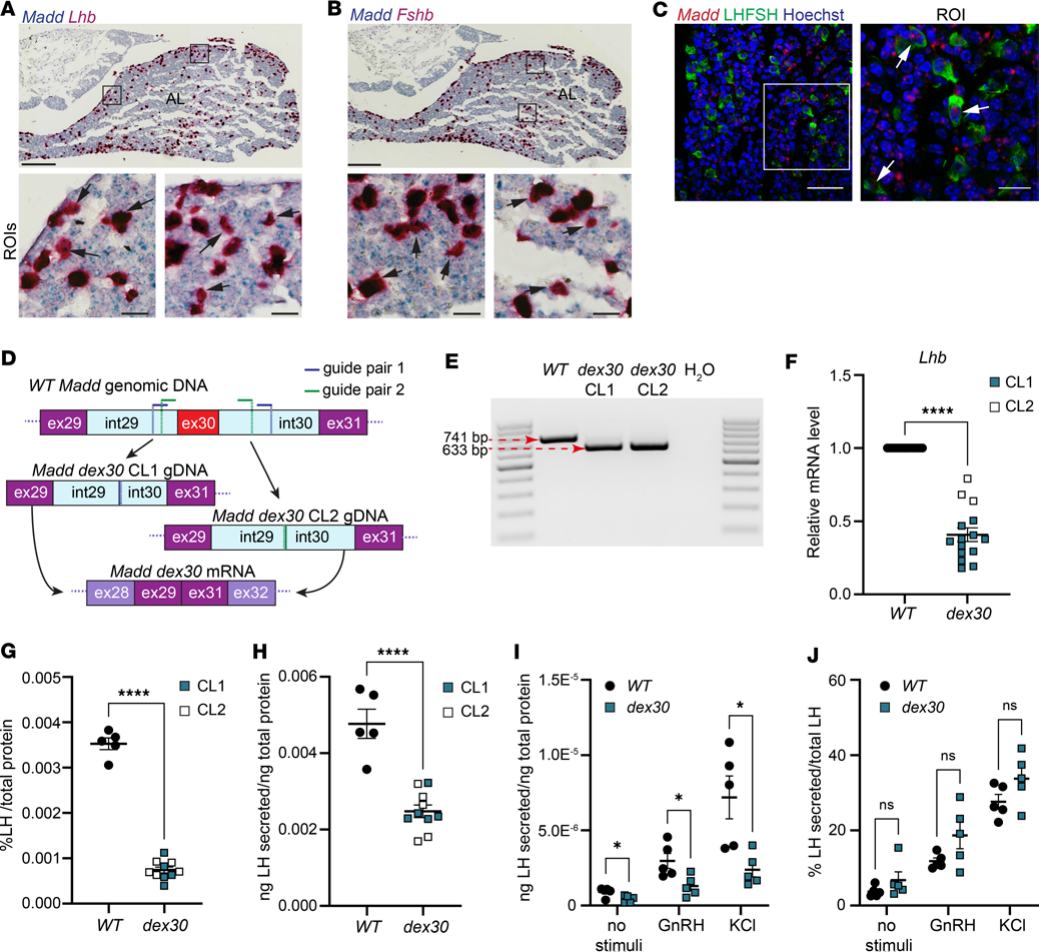
*Madd* is expressed in mouse pituitary gonadotropes; *dex30* leads to reduced expression and secretion of LH. (**A** and **B**) Representative RNAscope mRNA in situ hybridizations using probes against *Madd* (blue) and (**A**) *Lhb* or (**B**) *Fshb* (red) in adult (P45) mouse pituitary, coronal section. Arrows indicate examples of double-positive cells. Scale bar: 200 μm, ROI scale bar: 20 μm. AL, anterior lobe. (**C**) A representative RNAscope mRNA in situ hybridization using probes against *Madd* (red) combined with immunostaining using antibodies against LH+FSH (green) in adult (P45) mouse anterior pituitary. Arrows indicate examples of double-positive cells. Scale bar: 50 μm, ROI scale bar: 15 μm. RNAscope mRNA in situ hybridizations (**A**–**C**) were repeated 5–8 times. (**D**) A schematic of the genome-editing strategy used for generating *dex30* LβT2 clones. (**E**) An RT-PCR around *Madd* exon 30 showing truncated transcript in *dex30* LβT2 clones. (**F**) mRNA expression of *Lhb* in WT and *dex30* LβT2 cells (*n* = 15). (**G**) LH content in WT and *dex30* LβT2 cells normalized to total protein (*n* = 5 for WT, *n* = 10 for *dex30*). (**H**) Spontaneous LH secretion in WT and *dex30* LβT2 cells during 24 hours, normalized to total protein (*n* = 5 for WT, *n* = 10 for *dex30*). (**I** and **J**) LH secretion from WT and *dex30* LβT2 clone 1 during 20 minutes of static incubations with no stimuli, 50 nM GnRH, and 60 mM KCl (*n* = 5). Normalized to total protein (**I**) or LH content (**J**). ****P < 0.0001, and *P < 0.05 analyzed by Student’s *t* test (**F**–**H**) or multiple *t* tests (**I** and **J**).

We hypothesized that the skipping of *MADD* exon 30 may cause CHH by directly affecting the pituitary gonadotropes. We observed robust *MADD* mRNA expression in a human pituitary cDNA library by RT-PCR (Supplemental Figure 5A) and verified *MADD* expression in a human gonadotrope cluster by using Single Nucleus Pituitary Atlas (59) (Supplemental Figure 5F). Furthermore, we detected robust *Madd* expression in mouse gonadotropes by RNAscope RNA in situ hybridization (Figure 5, A–C) and reanalysis of a single-cell RNA-Seq data set from mouse pituitary (60) (Supplemental Figure 5G). Of note, we observed ubiquitous expression of *Madd* transcripts in multiple pituitary hormone–producing cells (Supplemental Figure 5, G and H).

As in vitro models of human gonadotropes are currently not available, we utilized a mouse immortalized gonadotrope cell line LβT2 (61) for disease modeling and targeted CRISPR/Cas9 with 2 guide RNAs to intronic sites around *Madd* exon 30 (Figure 5D). We identified 2 homozygous *dex30* LβT2 clones generated using 2 different guide RNA pairs (Figure 5, D and E). We did not observe differences in morphology (Supplemental Figure 6A) or viability (Supplemental Figure 6B) between wild-type and *dex30* LβT2 cells. *Madd* mRNA and protein levels were comparable between *dex30* and wild-type LβT2 cells (Supplemental Figure 6, C–E).

Interestingly, the expression of LH subunit β (*Lhb*) mRNA, LH content, and spontaneous LH secretion was significantly reduced in *dex30* LβT2 cells compared with wild-types (Figure 5, F–H). To investigate stimulated LH secretion, we performed sequential incubations with no stimuli, 50 nM GnRH, and 60 mM KCl. *Dex30* LβT2 cells secreted significantly less LH than the wild-types in all tested conditions when normalized to total protein (Figure 5I). However, the percentage LH secretion per LH content (Figure 5J) and stimulation indices (Supplemental Figure 6, F and G) were not affected, indicating that the secretory machinery remained functional in *dex30* LβT2 cells. To investigate whether *dex30* affects the responsiveness to GnRH stimulation, we primed *dex30* and wild-type LβT2 cells with pulsatile GnRH stimulations as described earlier (61). In line with the previous observations (61), the subsequent increase of *Lhb* mRNA expression was approximately 2.4-fold, with no differences between the genotypes (Supplemental Figure 6H), suggesting that *dex30* LβT2 cells are responsive to GnRH.

ERK1/2 phosphorylation is implicated in both basal and GnRH-stimulated *Lhb* expression (62). As MADD regulates ERK1/2 phosphorylation (16–18), we hypothesized that *dex30* may affect ERK1/2 phosphorylation in LβT2 cells. We quantified total and phosphorylated ERK1/2 by Western blot in wild-type and *dex30* LβT2 cells but did not detect significant differences in basal or GnRH-induced ERK1/2 phosphorylation (Supplemental Figure 6, I–K). These results suggest that *dex30* leads to reduced *Lhb* expression independently of ERK1/2 phosphorylation and does not compromise GnRH-induced ERK1/2 signaling. As the death domain of MADD interacts with TNFR1 (16–18), we also assessed ERK1/2 phosphorylation in LβT2 cells after 15 minutes of 50 ng/mL TNF-α stimulation, but we observed a very low overall responsivity to this stimulus in both wild-type and *dex30* cells (data not shown).

### Stability and activation of RAB3 proteins are not affected by dex30

*MADD* deficiency may reduce the stability of its target Rabs (4, 13). Therefore, we assessed the levels of RAB3A-D proteins by Western blot in *dex30* and wild-type LβT2 cells but observed no differences (Supplemental Figure 7, A and B). To test if *dex30* affects the GEF activity of MADD, we applied our CRISPR/Cas9 strategy in human embryonic kidney cell line HEK293 and generated 2 clones with homozygous *dex30* (Supplemental Figure 7C). We transiently expressed human RAB3A in *dex30* and wild-type HEK293 cells and quantified GTP-bound RAB3A by immunoprecipitation with conformation-specific antibody but observed no significant differences between *dex30* cells and wild-types (Supplemental Figure 7, D and E).

### Interactome of MADD reveals targets involved in multiple signaling pathways

**Figure 6:**
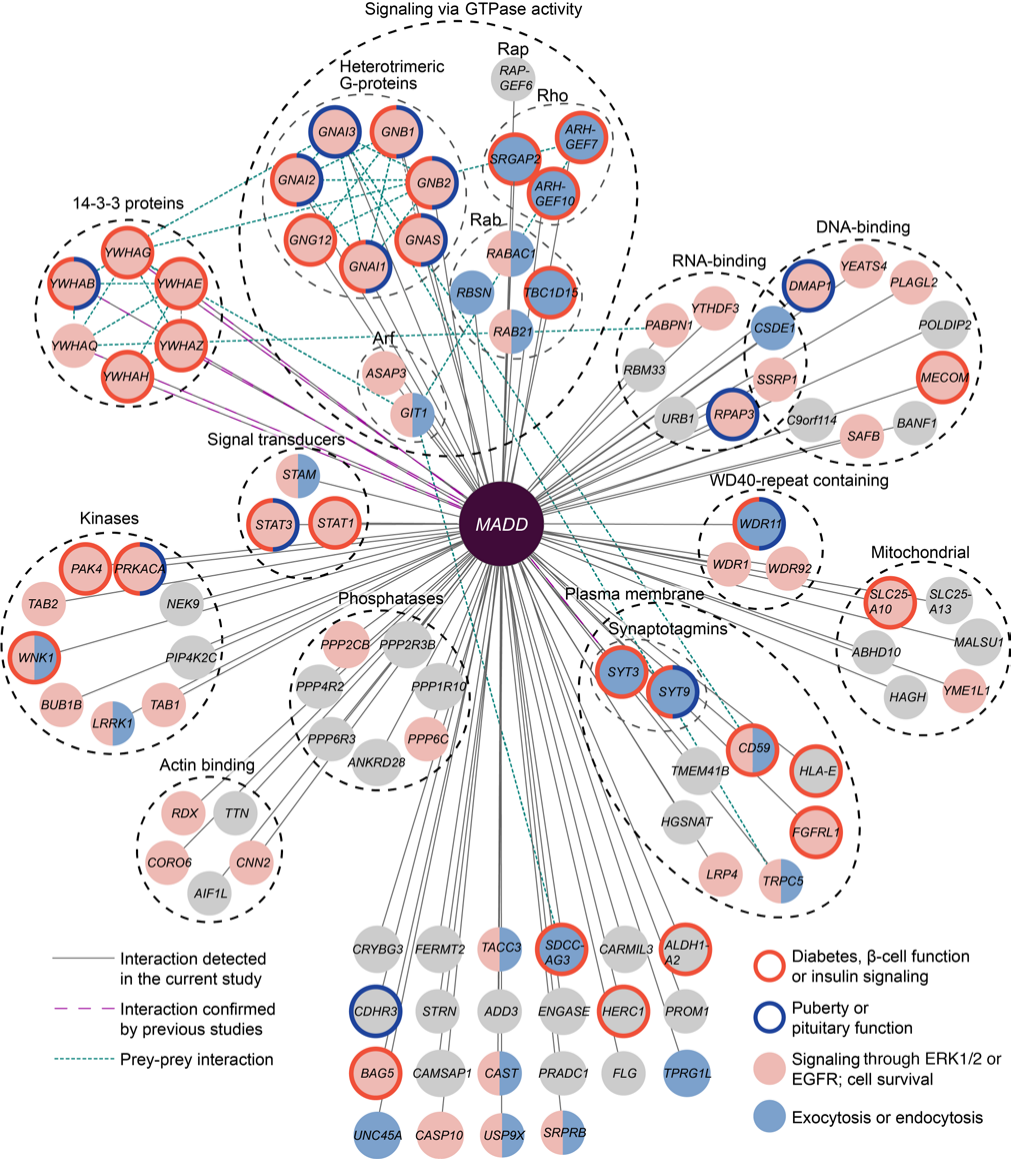
The interactome of MADD. AP-MS and BioID assays revealed 102 high-confidence interactors of WT and *dex30* MADD. Interactors implicated in endocrine phenotypes or pathways linked with MADD are highlighted with node and border colors. Interactors are grouped according to protein family, molecular function, or cellular compartment.

To further elucidate the effects of *dex30*, we detected the stable and transient protein-protein interactions (PPIs) of wild-type and *dex30* MADD in Flp-In T-REx 293 cells using affinity purification mass spectrometry (AP-MS) and proximity-based labeling mass spectrometry (BioID) assays (63). We detected in total 102 high-confidence interaction partners, of which 10 (9.8%) were detected by both methods (Figure 6 and Supplemental Table 3). Interactions between MADD and members of the 14-3-3 protein family and synaptotagmin 3 have been previously observed (64–68). Functionally, the largest group were the 17 interactors involved in signaling through GTPase activity, including 7 heterotrimeric G protein subunits and 10 regulators of Rho, Rab, Arf, and Rap subfamilies of small GTPases (Figure 6). In general, the interactors of MADD functioned in various signaling pathways, as they included 9 kinases, 7 serine/threonine-protein phosphatase subunits, 2 STATs, and 15 DNA and/or RNA binding proteins (Figure 6 and Supplemental Table 3).

Gene Ontology (GO) analysis revealed that the most significantly overrepresented biological processes in the interactome of *MADD* were positive regulation of protein insertion into mitochondrial membrane involved in apoptotic signaling pathway (GO:1900740), signal transduction (GO:0007165), and cellular protein localization (GO:0034613) (Supplemental Table 4). Furthermore, Reactome and Panther pathway analysis detected several overrepresented pathways, including signal transduction (R-HSA-162582), signaling by Rho GTPases (R-HSA-194315), EGF receptor signaling pathway (P00018), and FGF signaling pathway (P00021). Interestingly, diabetes-related pathways regulation of insulin secretion (R-HSA-422356), and integration of energy metabolism (R-HSA-163685), as well as puberty-related gonadotropin-releasing hormone receptor pathway (P06664), were enriched. Up to 50 enriched pathways are presented in Supplemental Table 5. Furthermore, we performed systematic PubMed searches and found that 35 of the interactors (34.3%) could be linked with diabetes/β cell function/insulin signaling and/or puberty/pituitary function and that 67 (65.7%) have been implicated in cellular functions previously linked with MADD (signaling through ERK1/2 or EGFR; exocytosis, endocytosis, or cell survival) (Figure 6 and Supplemental Table 6).

### Dex30 results in alterations in the interactome of MADD

**Figure 7:**
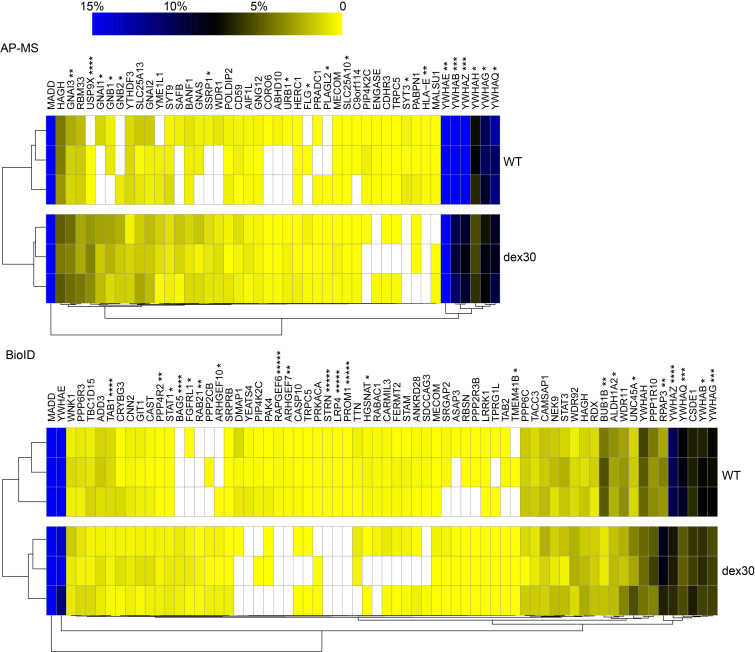
*Dex30* leads to changes in the interactome of MADD. Hierarchical clustering of the quantitated stable (AP-MS) and transient (BioID) high-confidence interactions of WT and *dex30* MADD from 3 replicates. The color gradient (0%–15%) illustrates the normalized abundances of the interactors relative to MADD bait protein. Interactions with significant differences between WT and *dex30* MADD are marked with asterisk (*****P < 0.00001, ****P < 0.0001, ***P < 0,001, **P < 0.01, *P < 0.05 analyzed by Student’s *t* test).

In total, we detected 36 proteins that interacted differently with *dex30* and wild-type MADD (Figure 7 and Supplemental Table 7). Interestingly, 52.8% of these interactors could be linked with diabetes/β cell function/insulin signaling and/or puberty/pituitary function (Supplemental Table 7). Compared with wild-types, the relative abundances were significantly reduced for 17 interactors and increased for 19 interactors in protein complexes isolated from *dex30* MADD-expressing cells, suggesting both loss-and gain-of-function effects. We detected the largest fold-change between wild-type and *dex30* MADD for the ubiquitin-specific peptidase 9 X-linked, whose relative abundance in protein complexes isolated from *dex30* MADD-expressing cells was approximately 24-fold higher compared with wild-type MADD-expressing cells. Notably, the AP-MS approach revealed that 6 members of 14-3-3 adaptor protein family (14-3-3β, 14-3-3ε, 14-3-3γ, 14-3-3η, 14-3-3θ, and 14-3-3ζ encoded by *YWHAB*, *YWHAE*, *YWHAG*, *YWHAH*, *YWHAQ*, and *YWHAZ*, respectively) interacted significantly less with *dex30* than wild-type *MADD* (Figure 7). This finding was replicated by the BioID approach for 14-3-3β, 14-3-3γ, 14-3-3θ, and 14-3-3ζ. On the other hand, 2 heterotrimeric G protein inhibitory α subunits (Gi; GNAI1 and GNAI3) and 2 β subunits (GNB1 and GNB2) interacted more with *dex30* compared with wild-type MADD. Both 14-3-3 proteins and subunits of heterotrimeric G proteins were highly represented in our pathway analysis (Supplemental Table 5), suggesting that *dex30* causes changes in multiple signaling pathways.

## Discussion

We identified an exon 30–skipping variant in *MADD* in 2 patients with developmental delay, diabetes, and pituitary hormone deficiency. In vitro disease-modeling experiments with *dex30* cells recapitulated the diabetes and CHH in the probands and suggested that *dex30* does not affect the GEF activity of MADD, but induces changes in the PPIs, likely affecting multiple signaling pathways.

The 2 patients with homozygous *MADD* exon 30–skipping variant were diagnosed with diabetes. Interestingly, polymorphisms at *MADD* locus have been previously associated with glycemic traits in genome-wide studies. The A allele at SNP rs7944584 and the G allele at SNP rs10501320 in *MADD* locus have been associated with higher fasting blood glucose levels (25, 27, 29) and rs7944584-A and 8 additional nonsynonymous *MADD* variants with type 2 diabetes (28). Results obtained with *dex30* cells implicate multiple mechanisms that may contribute to the development of diabetes. First, *dex30* caused reduced differentiation efficiency of stem cell–derived pancreatic progenitors at stages 4 and 5, leading to reduced number of β cells. *MADD* deficiency affects endocytosis of EGF (37), and the results of our PPI assays revealed that MADD interacts with 23 proteins implicated in EGFR signaling and that 52% of these interactions are altered by *dex30*. Given that EGFR signaling regulates pancreatic progenitor cell fate and organogenesis (69–71), and that EGF is one of the growth factors used in SC-islet differentiation during stages 3 and 4 (50), problems in EGFR signaling could explain the differentiation defect observed in *dex30* SC-islets, and similar effects may occur in vivo.

Second, *dex30* caused increased proinsulin-to-insulin ratio in SC-islets. Proinsulin levels have been associated with *MADD* locus in several genome- and exome-wide studies. A strikingly strong association (*P* = 2.07 × 10^–71^) was observed between *MADD* SNP rs7944584-A and higher fasting proinsulin levels (32). Further studies replicated this association (27, 29, 30) and identified additional SNPs rs10501320-G, rs10838687-T (29), rs1449626-A (34), and rs35233100-T (31) associated with higher fasting proinsulin levels. The increased proinsulin-to-insulin ratio observed in *dex30* SC-islets is consistent with results of previous studies and implies that *MADD* regulates proinsulin processing and trafficking, though the exact mechanism remains unresolved.

Third, the index patient had diabetes with normal peripheral insulin levels, suggesting insulin resistance for which the β cells failed to compensate. Our PPI studies revealed that 33 of the proteins interacting with MADD have been implicated in diabetes, β cell function, and insulin signaling and that 52% of these interactions were altered by *dex30*, including with members of the 14-3-3 protein family and STAT1, which are implicated in peripheral insulin signaling and pathogenesis of diabetes in pancreas, muscle, and adipose tissue (72–74). Taken together, our results suggest that skipping of *MADD* exon 30 may lead to diabetes by affecting both β cell function and peripheral insulin signaling. To our knowledge, diabetes has not been previously reported in patients with biallelic variants in *MADD* (37–39). This discrepancy may be age related, as in the probands of the current study, hyperglycemia was first observed at the ages of 14 and 21 years, whereas most of the previously reported patients were considerably younger, with a median age of 1.6 years and 83% being below 8 years of age (37–39). Therefore, we recommend that the patients with deleterious *MADD* variants are followed up until early adulthood for the presence of hyperglycemia and diabetes.

Notably, we did not observe reduced RAB3A activation or reduced RAB3 protein stability in *dex30* cells. RAB3 and RAB27 have been previously implicated in the exocytosis of pituitary hormones (75–77) and insulin (78–80), and it has been suggested that reduced activation of RAB3 and RAB27 may lead to problems in hormone secretion in patients with biallelic *MADD* variants (37). *Rab3a*- and *Rab27a*-deficient mice display reduced insulin secretion with normal or increased insulin content (79, 80), and conditional knockout of *Madd* in mouse β cells causes similar phenotype with disturbed exocytosis and accumulation of insulin granules (35). These results suggest that abolishment of *Madd* expression may affect insulin secretion by reducing activation of RAB3A/RAB27A. Instead, *dex30* results in altered protein-protein interactions that may produce a variety of loss- and gain-of-function effects leading to problems in insulin production while secretory machinery remains functional.

The probands of the current study had CHH and GHD. This is consistent with the previously observed hypopituitarism in the patients with biallelic *MADD* variants. CHH has not been diagnosed in patients with biallelic *MADD* variants, possibly due to their young age (37–39). However, frequently observed small penis or micropenis and cryptorchidism suggest disorders of the hypothalamic-pituitary-gonadal axis (37, 38) (Supplemental Figure 2). Concordantly with the CHH in the probands, we observed reduced LH content and secretion in *dex30* LβT2 gonadotropes. The relative GnRH responsiveness remained intact, suggesting GnRH receptor–independent mechanism of LH deficiency. However, we cannot exclude a hypothalamic component contributing to the CHH of the patients with *MADD* exon 30–skipping variant. Haploinsufficiency of rabconnectin-3a, which is a putative scaffolding protein for MADD and RAB3 GTPase activating protein (81), caused a similar disorder with CHH, diabetes, and developmental delay (82). Functional studies implicated rabconnectin-3a in GnRH neuron specification and maturation (82, 83). Similarly, it is possible that skipping of *MADD* exon 30 may affect maturation of GnRH neurons in vivo, though the ontogeny of *dex30* SC-derived GnRH neurons was not compromised.

Collectively, our results implicate *MADD* in the development and function of endocrine cells and suggest that it regulates multiple signaling pathways.

## Methods

### Sex as a biological variable.

This study included 1 female patient and 1 male patient, and similar findings are reported for both sexes.

### WGS and data filtering.

Genomic DNA was extracted from the peripheral blood of the patients using standard methodology. The WGS was performed using Illumina HiSeq X Ten technology at the BGI (Shenzhen, China). The impact of the identified SNVs was further evaluated with the web tools SIFT (46), PolyPhen2 (47), Human Splicing Finder v3.0 (41), SpliceAI/Pangolin (42), NNSplice (43), HAL Splice Prediction (44), and Spliceator (45).

Exon 30 and the exon-intron borders were PCR-amplified from the genomic DNA with primers 5′-CTGGATGGGGAATTCTTGGC-3′ and 5′-CGGTCCTATGAGTTCCCTGT-3′ with AmpliTaq Gold DNA Polymerase (Thermo Fisher Scientific). PCR products were purified with Illustra ExoProStar treatment (GE Healthcare, now Cytiva) and Sanger-sequenced at FIMM Genomics, University of Helsinki, Finland.

### RNA extraction and RT-PCR from patient’s blood.

RNA was extracted from fresh whole blood of the index patient and a healthy control with the QIAamp RNA Blood Mini Kit (QIAGEN). A total of 1 μg of RNA was converted to cDNA with SuperScript III First-Strand Synthesis System (Thermo Fisher Scientific). The area covering *MADD* transcript (NM_003682.3) was PCR-amplified in 8 overlapping amplicons (Supplemental Table 8). PCR products were purified and Sanger-sequenced.

### Minigene assay.

See Supplemental Methods.

### RT-PCR in human cDNA libraries/cadaveric islet cDNA.

RT-PCRs were performed from 2 μL of Human Brain Hypothalamus Marathon-Ready cDNA (Takara Bio), human pituitary cDNA (Clontech), and cDNA from human primary islets (Nordic Network for Islet Transplantation, Uppsala University, Sweden) with AmpliTaq gold polymerase (Thermo Fisher Scientific). Forward and reverse primers, respectively, were 5′-GCTTGTGGCGTAGAAATGGC-3′ and 5′-TCAGGTCCGGGTTTGATGC-3′ for *MADD* and 5′-ATGTTCGTCATGGGTGTGAA-3′ and 5′-GGTGCTAAGCAGTTGGTGGT-3′ for *GAPDH*.

### EndoC-βH1 cells.

EndoC-βH1 cells (48) were obtained from Univercell Biosolutions and cultured on Matrigel-coated (1%) and fibronectin-coated (2 μg/mL) (MilliporeSigma) plates in low-glucose (1 g/L) DMEM (Invitrogen, Thermo Fisher Scientific) as previously described (48).

### siRNA transfection of EndoC-βH1 cells.

A total of 2 × 10^5^ EndoC-βH1 cells were seeded on 24-well plates. The next day, cells were transfected with 30 nM ON-TARGETplus siRNA SMARTpool for human *MADD* (Horizon Discovery; L-004429-00-0005) or 30 nM ON-TARGETplus Non-targeting pool (Horizon Discovery; D-001810-10-05) using Lipofectamine RNAiMAX (Invitrogen, Thermo Fisher Scientific). Cells were assayed 96 hours posttransfection.

### Generating dex30 EndoC-βH1 cells.

*MADD* exon 30 was deleted by genome editing with CRISPR/Cas9. Two guide RNAs were designed with Benchling online tool (https://www.benchling.com). Guide sequences with protospacer adjacent motif sites marked in bold were: upstream from exon 30: 5′-CAGTCATACATCCCTCTCAG**AGG**-3′ and downstream from exon 30: 5′-AAGGGCTATTGAGAGTCACA**GGG**-3′. Guide RNAs and Cas9 were electroporated to cells as a complexed ribonucleoprotein (RNP) using the Neon Transfection System kit (Invitrogen, Thermo Fisher Scientific). Briefly, 2 million cells were dissociated using TrypLE (Thermo Fisher Scientific), pelleted at 250 relative centrifugal force (RCF) for 5 minutes, and resuspended in R-buffer and mixed with the RNP complex containing fluorescent Alt-R CRISPR-Cas9 tracrRNA, ATTO 550 (Integrated DNA Technologies), then electroporated with 1,100 V, 30 ms, and 2 pulses. ATTO 550^+^ cells were enriched using Sony SH800S Cell Sorter.

### Assessing gene expression in EndoC-βH1 cells.

Total RNA was extracted using MACHEREY-NAGEL RNA isolation kit. cDNA was prepared using the Maxima first-stand cDNA synthesis kit (Thermo Fisher Scientific). PCRs were prepared with Hot FIREPol EvaGreen qPCR mix plus (Solis Biodyne), and Ct values were determined using Corbett Rotor-Gene 6000 (QIAGEN). Relative gene expression of *dex30* versus wild-type cells was calculated by ΔΔCT method, using Cyclophilin-A as a housekeeping gene. All qPCR primers are listed in Supplemental Table 9.

### Measuring insulin secretion and content in EndoC-βH1 cells.

Cells were incubated in 1 mM glucose containing media overnight. The next day cells were equilibrated for 60 minutes in bKREBS (Univercell Biosolutions) without glucose and further stimulated in bKREBS with 1 mM glucose, 20 mM glucose, or 0.5 mM IBMX (MilliporeSigma) for 30 minutes. Supernatant was collected, and cells were washed with PBS and lysed with TETG solution (Univercell Bio-solutions). DNA content was determined using FluoReporter Blue dsDNA Kit (Invitrogen, Thermo Fisher Scientific). Secreted and intracellular insulin/proinsulin/C-peptide were measured using ELISA kits (Mercodia).

### Immunocytochemistry of EndoC-βH1 cells.

Cells were fixed with 4% (wt/vol) paraformaldehyde, permeabilized in 0.5% Triton X-100 (MilliporeSigma), and incubated overnight with primary antibodies (Supplemental Table 10) at 4°C.

### hESCs.

hESC line H9C11 with *GNRH1*-*TdTomato* reporter was generated in-house from H9 hESCs obtained from WiCell Research Institute (49) and maintained on Matrigel-coated dishes (Corning) in mTeSR1 (STEMCELL Technologies).

### Generating hESCs with dex30.

H9C11 cells were cultured with 5 μM ROCK inhibitor (Selleckchem) for 24 hours, dissociated with Accutase (Gibco, Thermo Fisher Scientific), and resuspended in cold 5% FBS in PBS. A total of 2 million cells were pelleted by centrifugation for 3 minutes at 300 RCF at room temperature (RT) and resuspended in 100 μL R-buffer (Invitrogen, Thermo Fisher Scientific). Guide RNAs used for EndoC-βH1 cells were electroporated to cells as RNP complexes including fluorescent ATTO 550 tracrRNA:crRNA and Alt-R S.p. Cas9 Nuclease V3 (Integrated DNA Technologies) with Neon Transfection system (Invitrogen, Thermo Fisher Scientific) with 2 pulses of 1,100 V for 20 ms. Cells were cultured in mTeSR1 and ROCK inhibitor for 24 hours, dissociated with Accutase, and resuspended in FACS buffer (10% FBS, 2 μM EDTA from Invitrogen, 0.625 mM HEPES from MilliporeSigma, and 10 μM ROCK inhibitor in HBSS from Invitrogen, Thermo Fisher Scientific). ATTO 550^+^ cells were single-cell–sorted with Sony SH800z sorter into 96-well plates. ROCK inhibitor was withdrawn after 4 days. Colonies that were 20%–80% confluent were dissociated with Accutase, and half of the suspension was pelleted in a V-bottom, 96-well plate and lysed in PCR Direct lysis buffer (Viagen Biotech) with 1.4 μg/μL of Proteinase K (QIAGEN). Correctly edited clones were identified by touchdown PCR with Phusion Hot-start II DNA polymerase (Thermo Fisher Scientific), with primers forward: 5′-GGCCTCTGAGACCAAAACATC-3′ and reverse: 5′- AAAATCACCTGGGCTTGG-3′, and verified by Sanger sequencing. RNA was isolated with NucleoSpin RNA Plus kit (MACHEREY-NAGEL). A total of 1 μg of RNA was converted to cDNA using iScript cDNA synthesis kit (Bio-Rad). RT-PCR amplification of the region between *MADD* exons 27–35 was performed from 25 ng of cDNA with AmpliTaq gold polymerase (Thermo Fisher Scientific), with primers for amplicon 7 in Supplemental Table 8. PCR products were Sanger-sequenced.

### Differentiation of SC-islets.

hESCs were cultured on Matrigel (Corning) in Essential 8 (Thermo Fisher Scientific), then differentiated to pancreatic SC-islets through a 7-stage differentiation protocol (50) (see also Supplemental Methods). For studying apoptosis, SC-islets were challenged with 1 μmol/L of thapsigargin (MilliporeSigma) for 48 hours.

### Gene expression during SC-islet differentiation.

Total RNA was extracted using the NucleoSpin RNA Plus kit. A total of 1.5 μg RNA was reverse-transcribed using the GoScript Reverse Transcriptase kit (Promega). We used 50 ng of cDNA for quantitative RT-PCR using 5x HOT FIREPol EvaGreen qPCR Mix Plus (Solis Biodyne). Reactions were performed in Rotor-Gene Q (QIAGEN) thermocycler. Gene expression relative to undifferentiated stem cells was calculated by ΔΔCt method, using Cyclophilin G (*PPIG*) as the housekeeping gene (Supplemental Table 9). Exogenous positive control was used as a calibrator.

### Flow cytometry during SC-islet differentiation.

Cells/SC-islets were dissociated with TrypLE for 3–10 minutes at 37°C and resuspended in 5% FBS-containing PBS. Stage 1 cells were stained with CXCR4 surface antibody for 20 minutes. Stage 4 cells and stage 7 SC-islets were fixed and permeabilized using Cytofix/Cytoperm (BD Biosciences) for 20 minutes. Primary antibodies were incubated overnight at 4°C and secondary antibodies for 30 minutes at RT in Perm/Wash buffer (BD Biosciences) + 5% FBS. FACS was performed with FACSCalibur cytometer (BD Biosciences); data were collected with CellQuest Pro v.4.0.2 (BD Biosciences) and analyzed with FlowJo v.10 (BD Biosciences). For antibodies, see Supplemental Table 10.

### Immunohistochemistry with SC-islets.

S7 SC-islets were fixed for 2 hours and embedded in paraffin. Sections of 5 μm were deparaffinized, and antigen retrieval was performed in 0.1 mmol/L citrate buffer in a pressure cooker (Biocare Medical). The slides were blocked with UV-block (Thermo Fisher Scientific) and incubated with primary antibodies (Supplemental Table 10) diluted in 0.1% Tween-20 overnight at 4°C and with secondary antibodies for 1 hour at RT. For detecting apoptosis, in situ Cell Death Detection Kit, Fluorescein (Roche), was used. The slides were imaged with Zeiss AxioImager using Apotome II with the same exposure and export setting for all slides of each immunostaining. Images were processed in Zen2 Blue Edition v.2 (ZEISS) and analyzed using CellProfiler v.4.0.

### Insulin secretion assays.

A total of 150 SC-islets were handpicked to 12-well plates and equilibrated in Krebs-Ringer buffer (KRB) with 2.8 mM glucose for 90 minutes, then subjected to sequential 30-minute incubations of 2.8 mM glucose, 16.8 mM glucose, 16.8 mM glucose + 50 ng/mL Ex4 (Tocris), and 2.8 mM glucose + 30 mM KCl in KRB. SC-islets were lysed in hypotonic conditions with sonication. Dynamic insulin secretion was assessed from another set of 150 SC-islets using a perifusion apparatus (Brandel Suprafusion SF-06) with a flow rate of 0.25 mL/min and sampling every 4 minutes. Insulin and proinsulin were quantified with ELISA kits (Mercodia), and DNA content was quantified using FluoReporter Blue dsDNA Kit (Invitrogen, Thermo Fisher Scientific).

### RNAscope mRNA in situ hybridization and immunohistochemistry in mouse hypothalamus and pituitary.

Brains from 10-week-old mice and pituitaries from 8-week-old mice (Charles River Laboratories, strain code 022) were fixed in 10% formalin (MilliporeSigma) overnight at RT. The tissue was paraffin-embedded following washes and dehydration through graded ethanol series. Samples were sectioned coronally at 4–5 μm. Double mRNA in situ hybridization was carried out with the RNAscope 2.5 HD Duplex Assay (ACD Bio). All probes used (*Madd*, *Gnrh1*, *Lhb*, *Fshb*, *Ghrh*, *dapB*, and *Polr2A*) were provided by the manufacturer. RNAscope-stained sections were scanned with a Nanozoomer-XR digital slide scanner (Hamamatsu). mRNA in situ hybridization combined with immunofluorescence was carried out using the RNAscope 2.5 HD Reagent Kit-RED assay (ACD Bio) and *Madd* probe with the following modification: Protease Plus was diluted 1:15 in PBS and incubated for 40 minutes at 40°C. After the Red detection step, slides were washed in PBS with 0.1% Triton X-100 (PBST) and incubated for 1 hour at RT in Blocking Buffer (0.15% glycine, 2 mg/mL BSA, 0.1% Triton X-100 in PBS) with 10% sheep serum. Primary antibodies (Supplemental Table 10) diluted in blocking buffer with 1% sheep serum were incubated at 4°C for 48 hours. Slides were washed in PBST and incubated with secondary antibodies for 1 hour at RT. Slides were washed before incubation with Hoechst (Invitrogen, Thermo Fisher Scientific) diluted 1:10,000 for 10 minutes at RT. Slides were washed and mounted with VectaMount (Vector Laboratories). Images were taken with a Leica TCS SP5 confocal microscope using an HCX Plan-Apochromat CS 20×/0.7 dry objective (Leica Microsystems).

### Single-cell RNA-Seq analysis.

Published data sets of mouse hypothalamus P45 (54) and of 7-week-old mouse pituitary (60) were reanalyzed following the respective published pipelines and interrogated for *Madd* expression using the Seurat package (84) in RStudio.

### Differentiation of SC-derived GnRH neurons.

hESCs were differentiated to GnRH-expressing neurons according to established protocol (57) with slight modifications. Briefly, differentiation was started (D0) in 90%–100% confluent cultures and carried out on Matrigel-coated plates in N2B27 basal medium. On days 0–9 cells were treated with 2 μM dorsomorphin (Selleckchem) and 10 μM SB431542 (MilliporeSigma). On day 10, cells were passaged with 200 U/mL collagenase IV and mechanical scraping, then replated at a dilution of 1:2 in N2B27 with 5 μM ROCK inhibitor. On days 11–19 cells were treated with 100 ng/mL FGF8 (PeproTech). On day 20 cells were passaged with PBS-EDTA and replated at a dilution of 1:8 in N2B27 + 100 ng/mL FGF8. On days 21–27 cells were treated with 20 μM DAPT (Selleckchem).

### Assessing tdTomato expression.

tdTomato signal was assessed from images acquired with ZOE fluorescent Cell Imager (Bio-Rad) on day 27 of differentiation. Red-colored cells from 6 images were counted manually using ImageJ (NIH).

### Immunocytochemistry with SC-derived GnRH neurons.

On day 20 of differentiation, cells were passaged with EDTA and seeded on Matrigel-coated glass coverslips. On day 27 cells were fixed with 4% paraformaldehyde for 15 minutes at RT and permeabilized in PBS containing 0.5% Triton X-100 (MilliporeSigma) for 7 minutes and blocked with BlockAid (Invitrogen, Thermo Fisher Scientific) for 30 minutes. Slides were incubated with primary antibodies (Supplemental Table 10) diluted in 0.1% Tween-PBS overnight at 4°C and with secondary antibodies for 1 hour at RT. Nuclei were counterstained with DAPI (MilliporeSigma). Slides were mounted with ProLong Diamond Antifade Mountant (Thermo Fisher Scientific), and images were captured with Zeiss AxioImager.Z1 upright epifluorescence microscope with 40×/NA 1.10 and 63×/NA 1.20 HC PL APO CS2 objectives (Biomedicum Imaging Unit) and processed in Zen2 Blue Edition v.2 (Zeiss).

### Assessing GnRH secretion.

Day 23 and 25 cultures (on a 35 mm dish) were washed once with DMEM, and 1.5 mL of N2B27 + DAPT was added. After 48 hours media were collected, and GnRH was measured by a fluorescent enzyme immunoassay (Phoenix Pharmaceuticals Inc).

### LβT2 cells.

LβT2 cells (61) were purchased from MilliporeSigma and maintained in high-glucose DMEM (MilliporeSigma) with 10% FBS and penicillin-streptomycin. Cells were passaged twice a week with Trypsin-EDTA at the ratio of 1:2–1:4. Cell morphology images were captured with Leica DM IL LED Inverted Microscope using Leica HI PLAN I 10×/NA 0.22 PH 1 objective, and viable cells were counted with TC20 automated cell counter (Bio-Rad) in the presence of 0.2% trypan blue (Gibco, Thermo Fisher Scientific).

### Generating LβT2 cells with Madd exon 30 deletion.

LβT2 cells were genome-edited like hESCs with a few exceptions. Two different guide RNA pairs were used. Sequences for pair 1 were upstream from exon 30: 5′-GGAGTCTATGAAGGGGGCACTGG-3′, downstream from exon 30: 5′-AACAAGGTGTGACCATAGCCAGG-3′, and for pair 2: upstream from exon 30: 5′-GGGGTGGGGAGTCTATGAAGGGG-3′, downstream from exon 30: 5′-TACCATAAAATGGCGTGCCTGGG-3′. LβT2 cells were harvested with Trypsin-EDTA and resuspended in cold PBS with 5% FBS. RNPs were mixed with 1 million LβT2 cells in 100 μL of R-buffer. Electroporation was performed with Neon Transfection system (Invitrogen, Thermo Fisher Scientific) with 2 pulses of 1,400 V for 25 ms. Cells were seeded on Matrigel-coated, 35 mm dishes with culture media with 30% FBS. After 48 hours cells were dissociated with Trypsin-EDTA and resuspended in FACS buffer (1% FBS, 5 μM EDTA, and 0.625 mM HEPES buffer from MilliporeSigma in HBSS from Life Technologies). ATTO 550^+^ cells were single-cell–sorted with BD Influx sorter into Matrigel-coated, 96-well plates with culture medium with 30% FBS. FBS concentration was reduced to 10% after 2 weeks. Clones that were 20%–80% confluent were screened for *Madd* exon 30 deletion by touchdown PCR with forward primer 5′-TGGCACAGGCCTTTAACTTC-3′ and reverse primer 5′-GCCTCAAACTCTGCCTTCAG-3′ as described for hESCs. PCR products were Sanger-sequenced. Promising clones were expanded. Deletion of *Madd* exon 30 was confirmed by RT-PCR with forward primer: 5′-TCCATGTGGGACCAGCTAGAG-3′ and reverse primer: 5′-CTCAGGCCCAGGTTTGATGC-3′. PCR products were Sanger-sequenced.

### Assessing gene expression in LβT2 cells.

For assessing gene expression in the naive state, cells were collected during normal passaging. For assessing gene expression after GnRH training, 2 million LβT2 cells were seeded on Matrigel-coated 12-well plates. Pulsatile GnRH training was started the following day by stimulating cells with 50 nM GnRH (MilliporeSigma) for 15 minutes followed by 2 washes with DMEM and 75 minutes of rest without GnRH. Stimulation was repeated 4 times a day for 3 consequent days. On the fourth day cells were harvested by trypsin-EDTA. RNA was isolated with NucleoSpin RNA kit. A total of 1 μg of RNA was reverse-transcribed using iScript cDNA synthesis kit (Bio-Rad). qPCR reactions were performed with 25 ng of first-strand cDNA, HOT 5x FIREPol EvaGreen qPCR Mix Plus, and 0.5 μM forward and reverse primers (Supplemental Table 9) in a LightCycler 480 (Roche). Expression levels in *dex30* cells relative to wild-types was calculated by ΔΔCt method, using TATA-box binding protein (*Tbp*) as a housekeeping gene.

### Quantification of LH.

A total of 3 million LβT2 cells were seeded on Matrigel-coated, 6-well plates and cultured until 70%–80% confluent. For assessing spontaneous LH secretion, cells were washed 2 times with culture media and supplied with 2 mL fresh medium. After 24 hours the media were collected. Cells were washed twice with ice-cold PBS and lysed by 10 minutes of incubation in ice-cold hypotonic lysis buffer (20 mM Tris-HCl at pH 7.6, 10 mM NaCl, 1 mM KCl, and 1.5 mM MgCl) supplemented with Protease inhibitor cocktail (Bio-Rad), followed by mechanical disruption by passing through a 29G needle 10 times. Lysates were centrifugated (12,000*g* for 10 minutes at 4°C) and supernatant was collected. Total protein was quantitated by bicinchoninic acid assay (BCA, Thermo Fisher Scientific). For stimulated LH secretion, 70%–80% confluent cells were washed twice with culture medium and incubated subsequently for 15 minutes with culture media, 50 nM GnRH (MilliporeSigma, L7134), and 60 mM KCl. Between stimuli cells were washed 2 times. Cells were lysed as described above. LH in the culture medium and lysates was quantitated by an immunofluorometric assay (85) at Reproductive Biology Unit, Turku Center for Disease Modeling, University of Turku, Finland.

### Immunoblot analysis.

A total of 1 million LβT2 cells were seeded on 12-well plates and cultured until 70%–80% confluent. Cells were washed twice with ice-cold PBS and lysed for 10 minutes in ice-cold RIPA buffer (Thermo Fisher Scientific) supplemented with Halt Protease inhibitor cocktail, EDTA (Thermo Fisher Scientific), and Halt Phosphatase inhibitor cocktail when needed. Lysates were centrifuged at 12,000*g* for 10 minutes at 4°C and supernatant was collected. Total protein was quantitated by BCA. We added 4× Laemmli sample buffer (Bio-Rad) with β-mercaptoethanol (Bio-Rad), and lysates were boiled at 95°C for 5 minutes. Proteins were separated on SDS-PAGE (5 μg of total protein per slot) and transferred to a PVDF membrane. Membranes were blocked with 5% nonfat milk or BSA (used for phosphorylated proteins) in TBS-Tween followed by incubation with primary antibodies overnight at 4°C and with HRP-linked secondary antibodies at RT for 1 hour. Chemiluminescence signals were captured with ChemiDoc Touch imager (Bio-Rad). Band intensities were quantified with Image Lab v6.0 software (Bio-Rad).

### ERK1/2 phosphorylation.

A total of 1 million LβT2 cells were seeded on 12-well plates and cultured until 70% confluent. Cells were starved overnight in 0.1% FBS. The next day, cells were treated for 15 minutes with 50 nM GnRH (MilliporeSigma) or 50 ng/mL TNF-α (PeproTech) in starvation media or left untreated. Total and phosphorylated ERK1/2 were quantitated by immunoblotting as described above.

### Generating dex30 HEK293 cells.

HEK293 cells were purchased from American Type Culture Collection and maintained like LβT2 cells, except that a split ratio of 1:10 was used. The same guide RNA sequences were used as for EndoC-βH1 cells and hESCs. Linear DNA templates for expression of guide RNAs were prepared by PCR amplification (see Supplemental Methods). A total of 500,000 HEK293 cells were seeded on a 35 mm dish. The next day, 300 ng of guide RNA expression cassettes for each guide and 3 μg of a plasmid encoding wild-type Cas9, GFP, and puromycin resistance gene (a gift from Diego Balboa, Biomedicum Stem Cell Centre, Helsinki, Finland; Addgene plasmid 89995) were transfected to cells with Lipofectamine 3000 (Invitrogen, Thermo Fisher Scientific). After 2 days 2.5 μg/mL puromycin (MilliporeSigma) was added. After 2 days of selection, cells were harvested by trypsin-EDTA, diluted to a concentration of 10 cells/mL, and replated on a 96-well plate (1 cell/well). 70%–80% confluent clones were screened for the absence of *MADD* exon 30 as described for hESCs.

### RAB3A activation assay.

A total of 140,000 HEK293 cells/well were seeded in a 24-well plate. The next day, cells were transfected with 100 ng of pEGFP–C1A-RAB3A (see Supplemental Methods) and 400 ng of empty vector with Lipofectamine 3000 (Invitrogen, Thermo Fisher Scientific). After 12 hours, the cells were subjected to RAB3A activation assay (NewEast Biosciences), based on pulldown of activated GTP-RAB3A with a conformation-specific antibody. Activated and total RAB3A were quantitated by immunoblot as described above.

### Generation of stable cell lines for PPI studies.

Constructs with *MADD* transcript ENST00000395336.7 with or without deletion of exon 30 in pcDNA3.1^+^ vector were generated at GenScript Biotech (the Netherlands). Entry clones compatible with the gateway system (Thermo Fisher Scientific) were generated according to the manufacturer’s instructions. MAC-tagged wild-type and *dex30* *MADD* constructs were generated using Gateway LR Clonase II Enzyme Mix according to the manufacturer’s instructions (Thermo Fisher Scientific).

The Flp-In T-REx 293 cell line was purchased from Invitrogen, Thermo Fisher Scientific, and grown according to the manufacturer’s instructions. Cells were cotransfected with MAC-tagged *MADD* constructs and pOG44 vector (Invitrogen, Thermo Fisher Scientific) using the Fugene6 reagent (Promega) for 48 hours. Positive isogenic clones were selected with 100 μg/mL hygromycin for 2 weeks.

The stable cell line was cultured on 150 mm plates until 80% confluent. Expression of MAC-tagged MADD was induced by 1 μg/mL tetracycline for 24 hours. For BioID labeling, 50 μM of biotin was added simultaneously with tetracycline. Cells from 5 dishes were pooled and pelleted by centrifugation at 1,200*g* for 5 minutes at 4°C. Three replicate pellets were prepared for downstream analyses.

### Purification and mass spectrometry.

Cells with only tetracycline treatment were used for affinity purification. Cells treated with biotin and tetracycline were used for the BioID approach. Cells were lysed and processed as previously described (63). Purified protein complexes were processed and digested to peptides for mass spectrometry analysis by Orbitrap Elite hybrid mass spectrometer utilizing Xcalibur version 2.0.7 SP1 (Thermo Fisher Scientific), connected to an EASY-nLC II reverse-phase HPLC system through an electrospray ionization sprayer (Thermo Fisher Scientific). Mass spectrometry analysis was in a data-dependent acquisition mode using Fourier-transform mass spectrometry full scan (*m/z* 300–1,700) resolution of 60,000 and collision-induced dissociation scan of the top 20 most abundant ions.

### Data processing.

For protein identification, Thermo.RAW files were searched against selected human UniProtKB/SwissProt database with Sequest search engine. All reported data were based on high-confidence peptides assigned in Proteome Discoverer (Thermo Fisher Scientific) with a 5% FDR by Percolator. Contaminant Repository for Affinity Purification (86) database and in-house GFP sample database were used as controls for identification of high-confidence interactions (87). Cytoscape 3.4.0 was used to construct the protein interaction network.

### Statistics.

Quantitative data were analyzed with GraphPad Prism 9 software and plotted as the mean ± standard error of the mean. Statistical significance was tested with Student’s *t* test or multiple *t* tests (all 2 tailed) with Holm-Šídák method for multiple comparisons. *P* values less than 0.05 were considered statistically significant.

### Study approval.

This study has been approved by the Ethics Committee of Helsinki University Hospital, Helsinki, Finland, and carried out according to the Declaration of Helsinki. The guardians of the patients gave their written informed consent. Mouse studies were approved by King’s College London Research Ethics Committee, London, United Kingdom.

### Data availability.

The quantitative data presented in the figures are available in the Supporting Data Values file. Mass spectrometry data were deposited to MassIVE (https://massive.ucsd.edu/) with the web accession MSV000093584.

## Author contributions

KP performed CRISPR editing and experimentation with hESC-derived GnRH neurons and LβT2 cells and RAB3A activation assays. JSV performed differentiations of SC-islets. PA conducted clinical investigations. XL and MV assayed PPIs. HI and VC performed experiments with EndoC-βH1 cells. AS and YK performed in situ hybridizations and single-cell RNA-Seq data analysis. YW performed part of the experiments with LβT2 cell clone 2. KV and API assisted in RAB3A activation assays, and KV was involved in data management. JK and JT performed the genetic analyses. KP and TR conceptualized the study and with SV, CLA, and TO designed the methodology. MV, SV, CLA, TO, and TR supervised the project. KP and JSV wrote the first manuscript draft. All the authors contributed to editing and accepted the final draft.

## Supplementary Material

Supplemental data

Unedited blot and gel images

Supplemental table 3

Supplemental table 5

Supplemental table 6

Supporting data values

## Version 1 05/22/2024

Electronic publication

## References

[1] Efimova EV (2004). IG20, in contrast to DENN-SV, (MADD splice variants) suppresses tumor cell survival, and enhances their susceptibility to apoptosis and cancer drugs. Oncogene.

[2] Wada M (1997). Isolation and characterization of a GDP/GTP exchange protein specific for the Rab3 subfamily small G proteins. J Biol Chem.

[3] Figueiredo AC (2008). Rab3GEP is the non-redundant guanine nucleotide exchange factor for Rab27a in melanocytes. J Biol Chem.

[4] Kat M (2021). GDP/GTP exchange factor MADD drives activation and recruitment of secretory Rab GTPases to Weibel-Palade bodies. Blood Adv.

[5] Zavala-Barrera C (2021). The calcium sensing receptor (CaSR) promotes Rab27B expression and activity to control secretion in breast cancer cells. Biochim Biophys Acta Mol Cell Res.

[6] Yamaguchi K (2002). A GDP/GTP exchange protein for the Rab3 small G protein family up-regulates a postdocking step of synaptic exocytosis in central synapses. Proc Natl Acad Sci U S A.

[7] Imai A (2013). MADD/DENN/Rab3GEP functions as a guanine nucleotide exchange factor for Rab27 during granule exocytosis of rat parotid acinar cells. Arch Biochem Biophys.

[8] Schutze K (2023). MAP kinase activating death domain deficiency is a novel cause of impaired lymphocyte cytotoxicity. Blood Adv.

[9] Zhong W (2023). Upregulation of exosome secretion from tumor-associated macrophages plays a key role in the suppression of anti-tumor immunity. Cell Rep.

[10] Tanaka M (2001). Role of Rab3 GDP/GTP exchange protein in synaptic vesicle trafficking at the mouse neuromuscular junction. Mol Biol Cell.

[11] Del-Rio-Robles JE (2023). CaSR links endocytic and secretory pathways via MADD, a Rab11A effector that activates Rab27B. Cell Signal.

[12] Niwa S (2008). KIF1Bbeta- and KIF1A-mediated axonal transport of presynaptic regulator Rab3 occurs in a GTP-dependent manner through DENN/MADD. Nat Cell Biol.

[13] Sanza P (2019). Nucleotide exchange factor Rab3GEP requires DENN and non-DENN elements for activation and targeting of Rab27a. J Cell Sci.

[14] Yoshimura S (2010). Family-wide characterization of the DENN domain Rab GDP-GTP exchange factors. J Cell Biol.

[15] Coppola T (2002). The death domain of Rab3 guanine nucleotide exchange protein in GDP/GTP exchange activity in living cells. Biochem J.

[16] Schievella AR (1997). MADD, a novel death domain protein that interacts with the type 1 tumor necrosis factor receptor and activates mitogen-activated protein kinase. J Biol Chem.

[17] Murakami-Mori K (1999). Implication of TNF receptor-I-mediated extracellular signal-regulated kinases 1 and 2 (ERK1/2) activation in growth of AIDS-associated Kaposi’s sarcoma cells: a possible role of a novel death domain protein MADD in TNF-alpha-induced ERK1/2 activation in Kaposi’s sarcoma cells. J Immunol.

[18] Kurada B (2009). MADD, a splice variant of IG20, is indispensable for MAPK activation and protection against apoptosis upon tumor necrosis factor-alpha treatment. J Biol Chem.

[19] Otmani K (2023). Acute myeloid leukemia-derived exosomes deliver miR-24-3p to hinder the T-cell immune response through DENN/MADD targeting in the NF-κB signaling pathways. Cell Commun Signal.

[20] Mulherkar N (2006). MADD/DENN splice variant of the IG20 gene is necessary and sufficient for cancer cell survival. Oncogene.

[21] Del Villar K, Miller CA (2004). Down-regulation of DENN/MADD, a TNF receptor binding protein, correlates with neuronal cell death in Alzheimer’s disease brain and hippocampal neurons. Proc Natl Acad Sci U S A.

[22] Lim KM (2004). Antisense abrogation of DENN expression induces apoptosis of leukemia cells in vitro, causes tumor regression in vivo and alters the transcription of genes involved in apoptosis and the cell cycle. Int J Cancer.

[23] Al-Zoubi AM (2001). Contrasting effects of IG20 and its splice isoforms, MADD and DENN-SV, on tumor necrosis factor alpha-induced apoptosis and activation of caspase-8 and -3. J Biol Chem.

[24] Li LC (2008). Regulation of apoptosis and caspase-8 expression in neuroblastoma cells by isoforms of the IG20 gene. Cancer Res.

[25] Dupuis J (2010). New genetic loci implicated in fasting glucose homeostasis and their impact on type 2 diabetes risk. Nat Genet.

[26] Flannick J (2019). Exome sequencing of 20,791 cases of type 2 diabetes and 24,440 controls. Nature.

[27] Florez JC (2012). Effects of genetic variants previously associated with fasting glucose and insulin in the diabetes prevention program. PLoS One.

[28] Hu C (2010). Variants from GIPR, TCF7L2, DGKB, MADD, CRY2, GLIS3, PROX1, SLC30A8 and IGF1 are associated with glucose metabolism in the Chinese. PLoS One.

[29] Strawbridge RJ (2011). Genome-wide association identifies nine common variants associated with fasting proinsulin levels and provides new insights into the pathophysiology of type 2 diabetes. Diabetes.

[30] Wagner R (2011). Glucose-raising genetic variants in MADD and ADCY5 impair conversion of proinsulin to insulin. PLoS One.

[31] Huyghe JR (2013). Exome array analysis identifies new loci and low-frequency variants influencing insulin processing and secretion. Nat Genet.

[32] Ingelsson E (2010). Detailed physiologic characterization reveals diverse mechanisms for novel genetic Loci regulating glucose and insulin metabolism in humans. Diabetes.

[33] Jensen AC (2011). Associations of common genetic variants with age-related changes in fasting and postload glucose: evidence from 18 years of follow-up of the Whitehall II cohort. Diabetes.

[34] Broadaway KA (2023). Loci for insulin processing and secretion provide insight into type 2 diabetes risk. Am J Hum Genet.

[35] Li LC (2014). IG20/MADD plays a critical role in glucose-induced insulin secretion. Diabetes.

[36] Marcheva B (2020). A role for alternative splicing in circadian control of exocytosis and glucose homeostasis. Genes Dev.

[37] Schneeberger PE (2020). Biallelic MADD variants cause a phenotypic spectrum ranging from developmental delay to a multisystem disorder. Brain.

[38] Abu-Libdeh B (2021). Homozygous variant in MADD, encoding a Rab guanine nucleotide exchange factor, results in pleiotropic effects and a multisystemic disorder. Eur J Hum Genet.

[39] Darouich S, Darouich S (2023). Calloso-adreno-scrotal agenesis associated with biallelic MAPK-activating death domain protein (MADD) variant: further phenotypic delineation of MADD deficiency. Am J Med Genet A.

[40] Genomes Project C (2015). A global reference for human genetic variation. Nature.

[41] Desmet FO (2009). Human Splicing Finder: an online bioinformatics tool to predict splicing signals. Nucleic Acids Res.

[42] Jaganathan K (2019). Predicting splicing from primary sequence with deep learning. Cell.

[43] Reese MG (1997). Improved splice site detection in Genie. J Comput Biol.

[44] Rosenberg AB (2015). Learning the sequence determinants of alternative splicing from millions of random sequences. Cell.

[45] Scalzitti N (2021). Spliceator: multi-species splice site prediction using convolutional neural networks. BMC Bioinformatics.

[46] Ng PC, Henikoff S (2001). Predicting deleterious amino acid substitutions. Genome Res.

[47] Adzhubei IA (2010). A method and server for predicting damaging missense mutations. Nat Methods.

[48] Ravassard P (2011). A genetically engineered human pancreatic β cell line exhibiting glucose-inducible insulin secretion. J Clin Invest.

[49] Lund C (2020). Characterization of the human GnRH neuron developmental transcriptome using a *GNRH1*-TdTomato reporter line in human pluripotent stem cells. Dis Model Mech.

[50] Balboa D (2022). Functional, metabolic and transcriptional maturation of human pancreatic islets derived from stem cells. Nat Biotechnol.

[51] Vasiljevic J (2020). The making of insulin in health and disease. Diabetologia.

[52] Ramzy A (2020). Revisiting proinsulin processing: evidence that human β-cells process proinsulin with prohormone convertase (PC) 1/3 but not PC2. Diabetes.

[53] Kaprara A, Huhtaniemi IT (2018). The hypothalamus-pituitary-gonad axis: tales of mice and men. Metabolism.

[54] Kim DW (2020). The cellular and molecular landscape of hypothalamic patterning and differentiation from embryonic to late postnatal development. Nat Commun.

[55] Wang Y (2022). Deciphering the transcriptional landscape of human pluripotent stem cell-derived GnRH neurons: the role of Wnt signaling in patterning the neural fate. Stem Cells.

[56] Vastagh C (2015). The spatiotemporal segregation of GAD forms defines distinct GABA signaling functions in the developing mouse olfactory system and provides novel insights into the origin and migration of GnRH neurons. Dev Neurobiol.

[57] Lund C (2016). Development of gonadotropin-releasing hormone-secreting neurons from human pluripotent stem cells. Stem Cell Reports.

[58] Yellapragada V (2019). MKRN3 interacts with several proteins implicated in puberty timing but does not influence GNRH1 expression. Front Endocrinol (Lausanne).

[59] Zhang Z (2022). Single nucleus transcriptome and chromatin accessibility of postmortem human pituitaries reveal diverse stem cell regulatory mechanisms. Cell Rep.

[60] Cheung LYM (2018). Single-cell RNA sequencing reveals novel markers of male pituitary stem cells and hormone-producing cell types. Endocrinology.

[61] Turgeon JL (1996). Steroid and pulsatile gonadotropin-releasing hormone (GnRH) regulation of luteinizing hormone and GnRH receptor in a novel gonadotrope cell line. Mol Endocrinol.

[62] Harris D (2002). Activation of MAPK cascades by GnRH: ERK and Jun N-terminal kinase are involved in basal and GnRH-stimulated activity of the glycoprotein hormone LHbeta-subunit promoter. Endocrinology.

[63] Liu X (2018). An AP-MS- and BioID-compatible MAC-tag enables comprehensive mapping of protein interactions and subcellular localizations. Nat Commun.

[64] Angrand PO (2006). Transgenic mouse proteomics identifies new 14-3-3-associated proteins involved in cytoskeletal rearrangements and cell signaling. Mol Cell Proteomics.

[65] Collins BC (2013). Quantifying protein interaction dynamics by SWATH mass spectrometry: application to the 14-3-3 system. Nat Methods.

[66] Huttlin EL (2017). Architecture of the human interactome defines protein communities and disease networks. Nature.

[67] Jayarama S (2014). MADD is a downstream target of PTEN in triggering apoptosis. J Cell Biochem.

[68] Segal D (2023). A central chaperone-like role for 14-3-3 proteins in human cells. Mol Cell.

[69] Lof-Ohlin ZM (2017). EGFR signalling controls cellular fate and pancreatic organogenesis by regulating apicobasal polarity. Nat Cell Biol.

[70] Nostro MC (2015). Efficient generation of NKX6-1+ pancreatic progenitors from multiple human pluripotent stem cell lines. Stem Cell Reports.

[71] Miettinen PJ (2000). Impaired migration and delayed differentiation of pancreatic islet cells in mice lacking EGF-receptors. Development.

[72] Pennington KL (2018). The dynamic and stress-adaptive signaling hub of 14-3-3: emerging mechanisms of regulation and context-dependent protein-protein interactions. Oncogene.

[73] Rial SA (2023). Is 14-3-3 the combination to unlock new pathways to improve metabolic homeostasis and β-cell function?. Diabetes.

[74] Gurzov EN (2016). The JAK/STAT pathway in obesity and diabetes. FEBS J.

[75] Tasaka K (1998). Rab3B is essential for GnRH-induced gonadotrophin release from anterior pituitary cells. J Endocrinol.

[76] Gomi H (2007). Rab27b is expressed in a wide range of exocytic cells and involved in the delivery of secretory granules near the plasma membrane. Mol Biol Cell.

[77] Zhao S (2002). Involvement of Rab27b in the regulated secretion of pituitary hormones. Endocrinology.

[78] Cazares VA (2014). Distinct actions of Rab3 and Rab27 GTPases on late stages of exocytosis of insulin. Traffic.

[79] Yaekura K (2003). Insulin secretory deficiency and glucose intolerance in Rab3A null mice. J Biol Chem.

[80] Kasai K (2005). Rab27a mediates the tight docking of insulin granules onto the plasma membrane during glucose stimulation. J Clin Invest.

[81] Nagano F (2002). Rabconnectin-3, a novel protein that binds both GDP/GTP exchange protein and GTPase-activating protein for Rab3 small G protein family. J Biol Chem.

[82] Tata B (2014). Haploinsufficiency of Dmxl2, encoding a synaptic protein, causes infertility associated with a loss of GnRH neurons in mouse. PLoS Biol.

[83] Tata BK (2017). Rabconnectin-3α is required for the morphological maturation of GnRH neurons and kisspeptin responsiveness. Sci Rep.

[84] Stuart T (2019). Comprehensive integration of single-cell data. Cell.

[85] Haavisto AM (1993). A supersensitive immunofluorometric assay for rat luteinizing hormone. Endocrinology.

[86] Mellacheruvu D (2013). The CRAPome: a contaminant repository for affinity purification-mass spectrometry data. Nat Methods.

[87] Liu X (2020). Combined proximity labeling and affinity purification-mass spectrometry workflow for mapping and visualizing protein interaction networks. Nat Protoc.

