# Supplementary material for: A splice site variant in MADD affects hormone expression in pancreatic β cells and pituitary gonadotropes
Source: JCI Insight. 2024 May 22;9(10):e167598. doi: 10.1172/jci.insight.167598 (PMC11141940; doi:10.1172/jci.insight.167598)
Supplement: Supplemental data [file jciinsight-9-167598-s174.pdf]

A splice-site variant in *MADD* affects hormone expression in pancreatic β-cells and pituitary gonadotropes

Supplemental material

Table of Contents

Clinical description of the patients 2
Supplemental Figures 4
Supplemental Tables........ 15
Supplemental Methods........ 22
Supplemental References 28

Clinical description of the patients

Patient 1.

The index patient (individual V3 in Fig. 1A) was the third child born to first cousins once removed. The pregnancy and birth were uncomplicated, and the weight at birth was approximately 3.5 kg. The neonatal phase was normal, but the patient developed seizures at the age of 6-7 months and received phenobarbital until the age of 9 years. She had a developmental delay, and she started walking and speaking single words at the age of 3-3.5 years. A computerized tomography scan at the age of 9 years was interpreted as normal. At the age of 12 years, she understood simple verbal instructions and spoke ~20 words. Her motor function was normal. She had slightly elevated palate, low-positioned ears, and somewhat low hairline. No body measurements before the age of 12 years were available, but between 12-14 years she grew 0.7 SD below her target height at -1.4 SD. At the age of 14 years elevated HbA1c level of 52 mmol/mol was observed, and an oral glucose tolerance test (OGTT) was performed. Her fasting plasma glucose level was 7.6 mmol/l and 2 hours after glucose challenge it remained 14.1 mmol/l, reaching the threshold of diabetes. OGTT was repeated at the age of 15 years with assessment of insulin and C-peptide levels which were considered normal: (fasting insulin 5.4 mU/l, 2h post glucose: 117.0 mU/l; fasting C-peptide 0.49 nmol, 2h post glucose: 3.86 nmol). Her fasting and 2h plasma glucose remained high, 7.9 mmol/l and 18.1 mmol/l, respectively. Levels of type-1 diabetes autoantibodies were within normal limits (islet cell autoantibodies 0 Juvenile Diabetes Foundation (JDF) units, glutamic acid decarboxylase antibodies 0.10 Relative Units (RU), insulin autoantibodies 10%). These results suggested that the patient had insulin resistance, which her beta cells failed to compensate. She was diagnosed with diabetes mellitus and treated with metformin. Between 15-19 years of age her HbA1c levels remained high (46-53 mmol/mol).

At the age of 14 years, the patient was pre-pubertal with no breast development or pubic hair, and a hormonal workup was conducted. Her serum estradiol level was prepubertal (0.008 nmol/l). Her basal LH level was notably low (0.1 IU/L) with maximal increase to 1.7 IU/l after stimulation with GnRH. These findings were suggestive of gonadotropin deficiency, and the patient started estrogen therapy. Her basal FSH level was 1.6 IU/l with maximal increase to 8.4 IU/l after GnRH stimulation. Patient's serum IGF-1 level was low (9.3 nmol/l). Her basal GH level was <0.05 µg/l with maximal increase to 0.94 µg/l after arginine stimulation, indicating GHD. However, she did not receive GH therapy. Her thyroid and adrenal functions were normal. MRI of the brain and the olfactory tract did not reveal anomalies.

At the age of 19 years the patient's puberty was incomplete despite estrogen therapy (M3P3 according to the Tanner scale). GnRH test revealed persistent low LH response (from <0.1 IU/l to 2.0 IU/l after GnRH stimulation). These findings were consistent with CHH. Patient's FSH level increased from 2.0 IU/l to 9.2 IU/l after GnRH stimulation. The estrogen therapy induced some acceleration of growth, and by the age of 19 years the patient had reached her target height. However, repeated arginine test revealed subnormal GH response (from 0.66 µg/l to 3.01 ug/l after arginine stimulation), confirming GHD.

Patient 2.

An elder brother of patient 1, (individual V2 in Fig. 1A) had severe developmental delay and epilepsy. Brain MRIs were performed at the ages of 7 and 21 years with normal findings. Elevated fasting plasma glucose level of 6.5 mmol/l and HbA1c level of 46 mmol/mol was first observed at the age of 21 years. At the age of 28 years the patient developed polyuria. Substantially elevated fasting plasma glucose level of 14.7 mmol/l and HbA1c level of 73 mmol/mol were observed, and metformin was introduced.

At the age of 21 years the patient had pre-pubertal penis and testes and he lacked pubic hair. Hormonal workup revealed prepubertal levels of testosterone (0.3 nmol/l) and LH (<0.5 IU/l), indicating absent puberty due to CHH. Patient's IGF-1 level was suggestive of GHD (10.3 nmol/l). His thyroid function was normal.

Supplemental Figures

Supplemental Figure 1

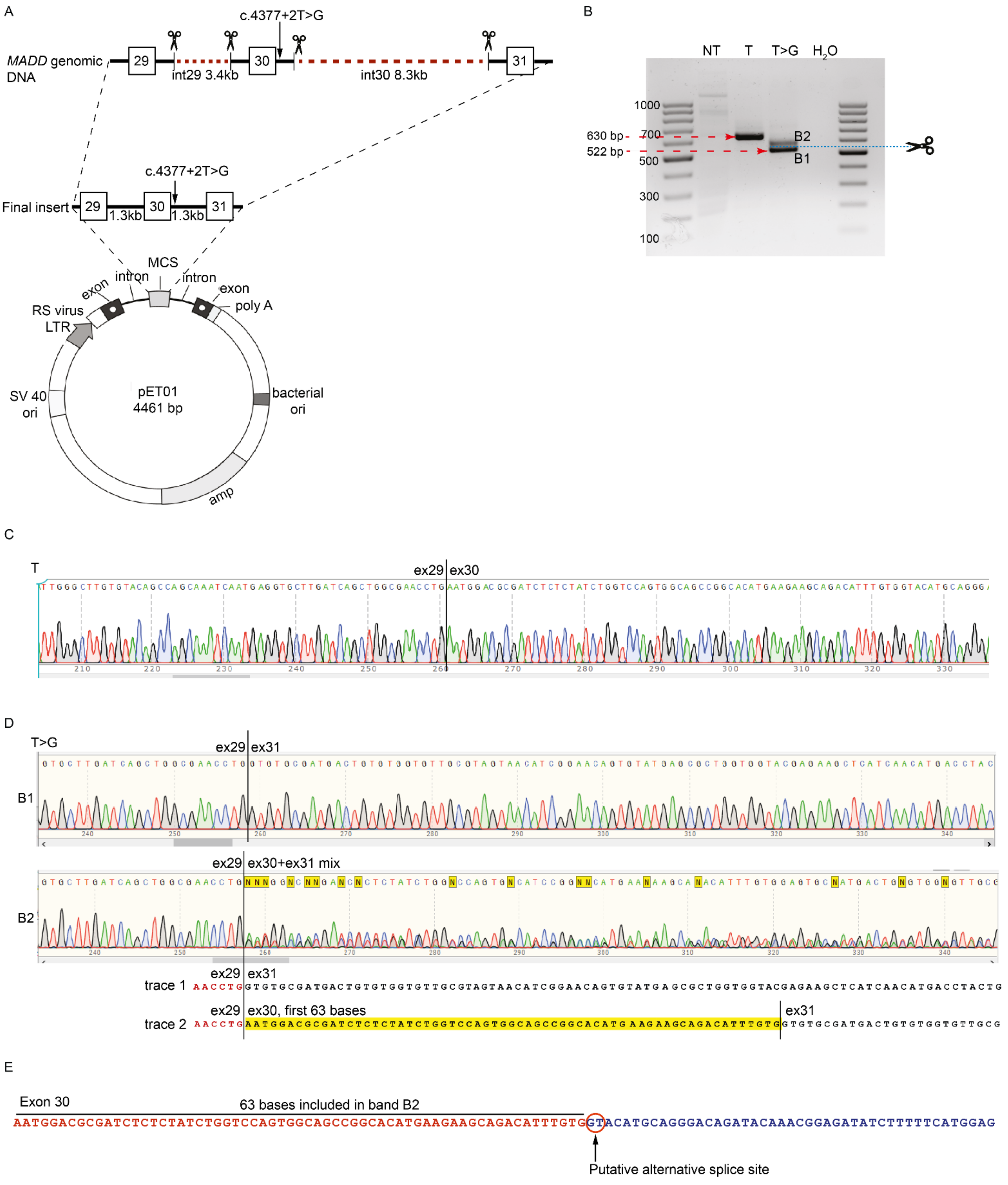

Figure S1. A minigene assay confirms the effect of *MADD* c.4377+2T>G variant on splicing.
(A) A Schematic of the pET01-vector-based construct used in the minigene assay. Insert containing *MADD* exons 29-31 and part of the surrounding intronic sequences with c.4377+2T or c.4377+2T>G variant were inserted in the multiple cloning site (MCS) surrounded by introns and exons. Due to size restrictions, 2040 bp of intron 29 and 7065 bp of the intron 30 had to be trimmed off (marked with a dark red dashed line). **(B)** c.4377+2T (T) and c.4377+2T>G (T>G) constructs were transiently expressed in HEK293 cells, and the inclusion of exon 30 was assessed by RT-PCR with primers specific to pET01-vector exons. An agarose gel electrophoresis shows that the T-construct produces one band with size of 630 bp corresponding to inclusion of all three *MADD* exons, whereas construct with T>G variant produces two bands, one with size of 522 bp corresponding to complete skipping of exon 30 (B1) and one of intermediate size (B2). B1 and B2 bands were separately purified from the gel. **(C-D)** The PCR product produced by the T-construct and the gel-purified B1 and B2 bands produced by T>G construct were Sanger sequenced. **(C)** Cells transfected with the T-construct express mRNA containing *MADD* exon 30. **(D)** B1 band from cells transfected with T>G construct corresponds to mRNA where exon 30 is completely skipped. B2 band chromatogram reveals two traces, one showing complete skipping of exon 30 (possibly due to incomplete separation of B1 and B2 bands), whereas the second trace consists of 63 first bases of exon 30 followed by exon 31. **(E)** The 63 first bases of exon 30 are followed by a GT-site, which the cells appear to have used as an alternative splice donor site in the minigene assay. However, as several kilobases of intronic sequences were deleted from the constructs, possibly removing regulatory sequences that affect recognition of splice sites, and as complete skipping of exon 30 was observed in RT-PCR with patient-derived RNA (Figure 1D-E), it is not likely that the mRNA with partial inclusion of exon 30 is highly expressed in vivo.

Supplemental Figure 2

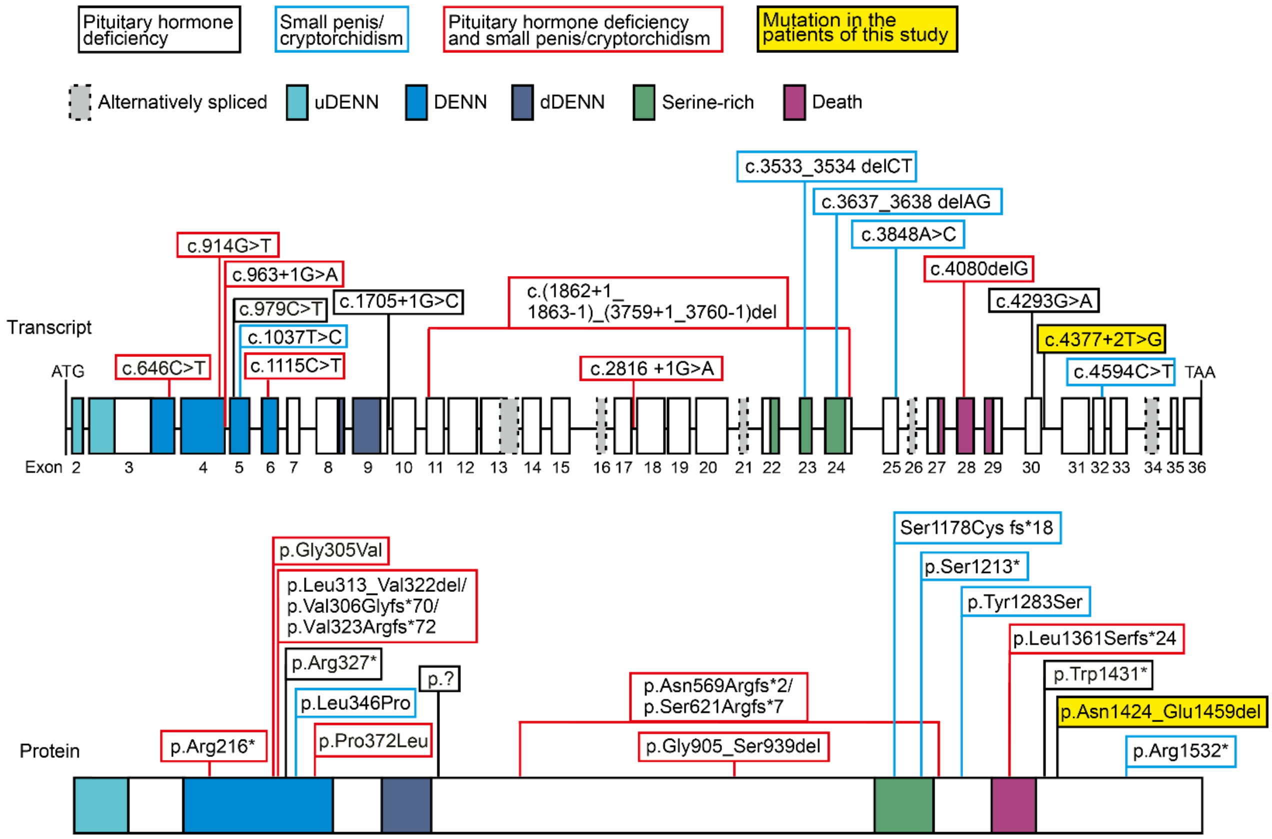

Figure S2. Biallelic mutations in *MADD* causing hypopituitarism and small penis/cryptorchidism
A schematic of *MADD* transcript and protein with the current and previously reported biallelic mutations in patients with hypopituitarism and/or small penis and cryptorchidism. Alternatively spliced exons and subunits of the DENN-domain (uDENN, DENN and dDENN), serine-rich-domain and Death-domain are indicated. Not in scale. Modified from Figure 1 of Supplemental reference (1).

Supplemental Figure 3

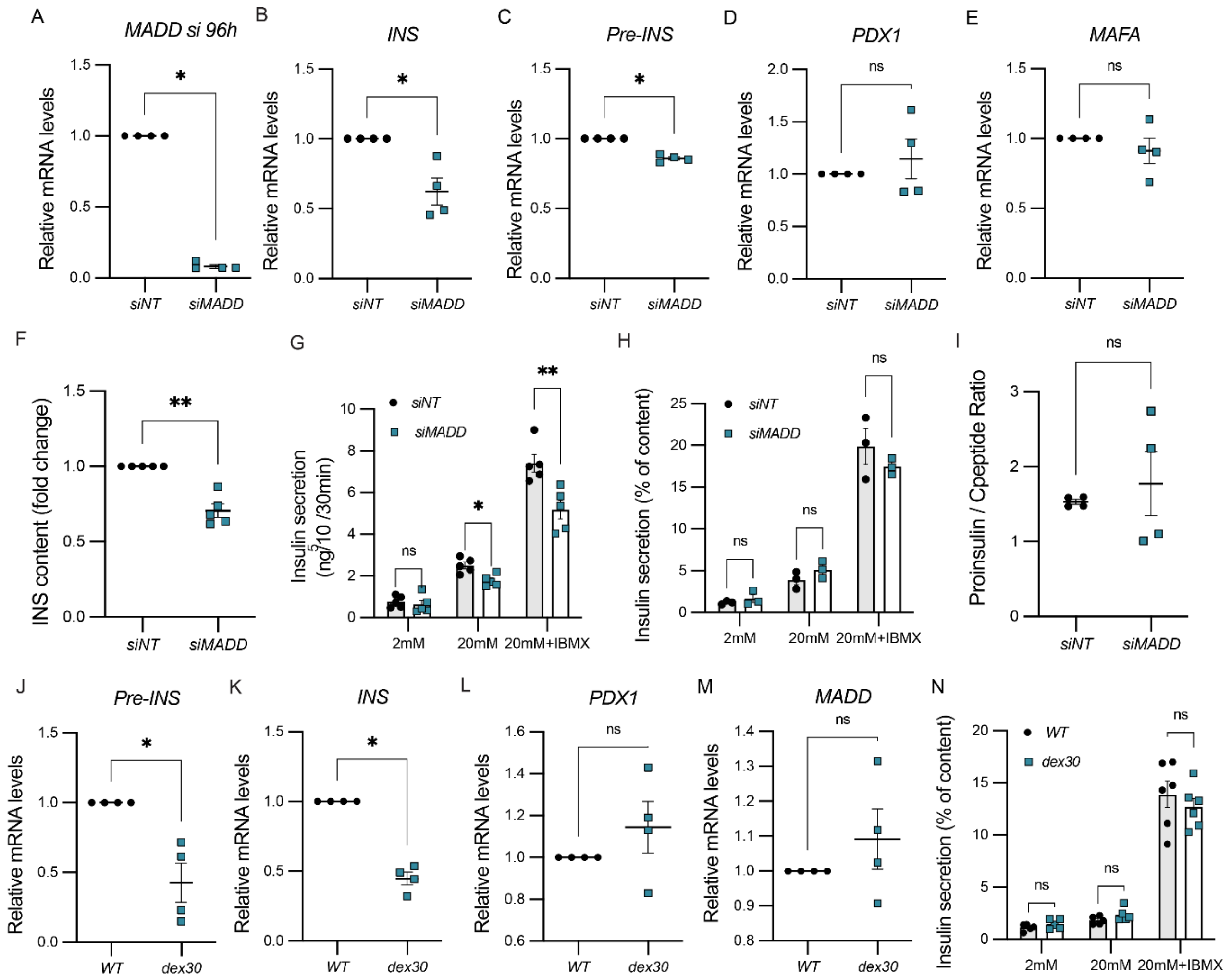

Figure S3. Silencing of *MADD* expression in EndoC-βH1 cells causes decreased insulin content. (A-E) Relative mRNA expression after 96 h treatment with non-targeting (siNT) or *MADD*-specific (si*MADD*) siRNAs in EndoC-βH1 cells (n=4). **(A)** *MADD*, **(B)** *INS*, **(C)** *Pre-INS*, **(D)** *PDX1* and **(E)** *MAFA*. **(F)** Insulin content in si*MADD* treated EndoC-βH1 cells expressed as fold-change to siNT-treated cells (n=5). **(G-H)** Insulin secretion from siNT and si*MADD*-treated EndoC-βH1 cells in 2mM glucose, 20 mM glucose and 20 mM glucose with IBMX. **(G)** as per 10^5 cells (n=5) **(H)** as % of insulin content, (n=3). **(I)** Proinsulin-to-C-peptide ratio in siNT and si*MADD*-treated EndoC-βH1 cells (n=4). **(J-M)** Relative mRNA expression of **(J)** *Pre-INS*, **(K)** *INS*, **(L)** *PDX1* and **(M)** *MADD* in wild type and *dex30* EndoC-βH1 cells (n=4). **(N)** Static insulin secretion from WT and *dex30* EndoC βH1 cells as % of insulin content in 2mM glucose, 20 mM glucose and 20 mM glucose with IBMX (n=6). **p<0.01, *p<0.05 analyzed by Student's *t*-test **(A-F, I-M)** or multiple *t*-tests **(G-H, N).**

Supplemental Figure 4

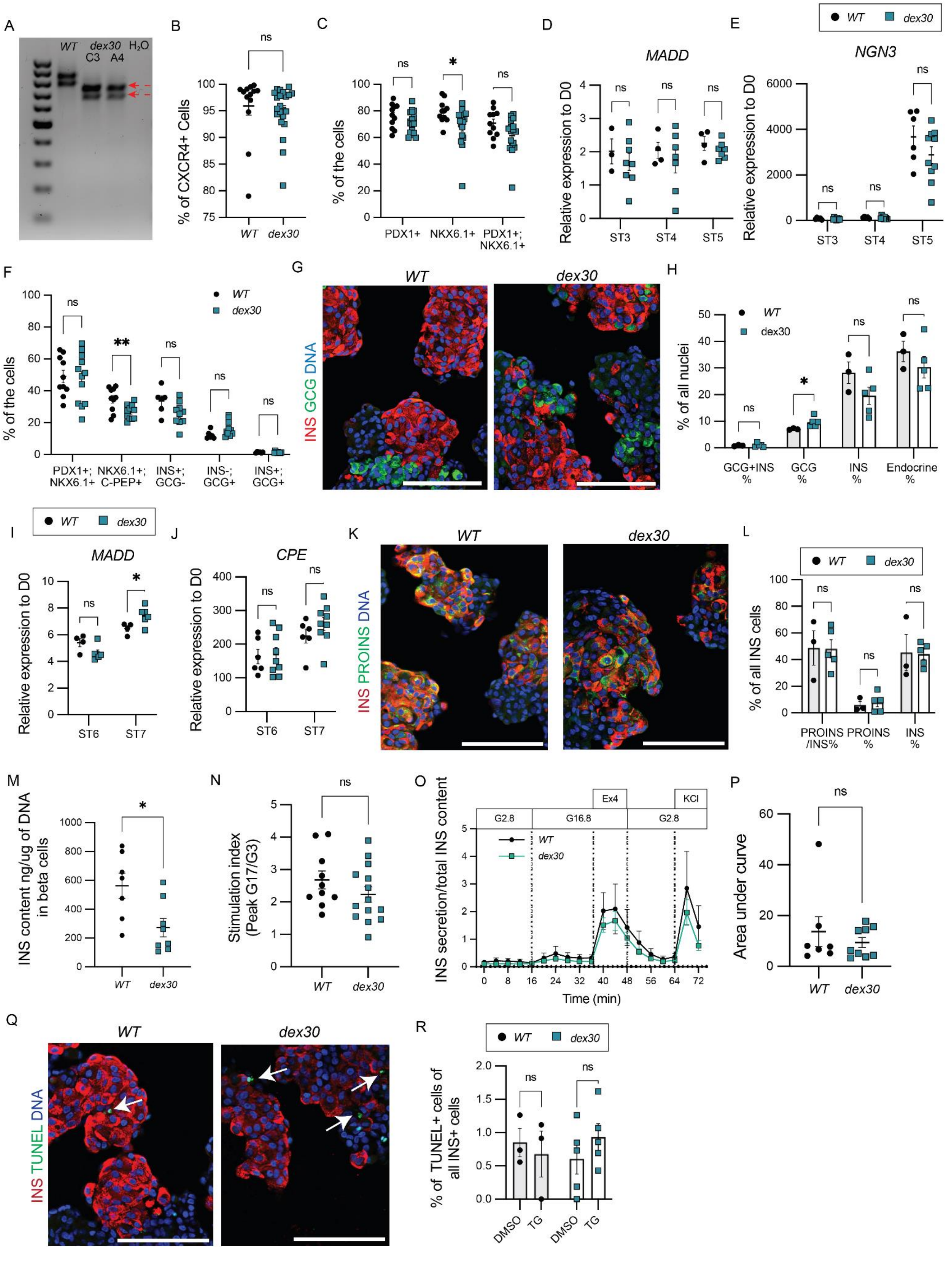

Figure S4. Differentiation of WT and *dex30* hESCs to SC-islets. (A) RT-PCR with primers targeting around *MADD* exon 30 showing truncated transcript in two homozygous hESC clones C3 and A4. The two bands indicate the expression of two *MADD* splice isoforms (with and without alternatively spliced exon 34). **(B)** Flow cytometry with CXCR4 surface marker at definitive endoderm stage (ST1) (n=13 in WT, n=22 in *dex30*), **(C)** Flow cytometry with PDX1 and NKX6.1 antibodies at pancreatic progenitor stage (ST4) (n=11 in WT, n=17 in *dex30*). **(D-E)** mRNA expression levels of **(D)** *MADD*, **(E)** *NGN3* during pancreatic progenitor stages ST3, ST4 and ST5 relative to non-differentiated cells (n=3-6 in WT, n=7-10 in *dex30*). **(F)** Flow cytometry with PDX1-, NX6.1-, C-peptide (CPEP)-, insulin (INS)- and glucagon (GCG)-specific antibodies in WT and *dex30* SC-islets at ST7 w3 (n=6-10 in WTs, n=10-12 in *dex30*). **(G)** Immunohistochemistry with insulin and glucagon-specific antibodies at ST7 SC-islets Scale bars 100 μm. **(H)** Quantification of insulin$^+$glucagon$^+$ double positive (GCGINS%), glucagon$^+$ only (GCG%), insulin$^+$ only (INS%) or all these together (endocrine) as a % of all nuclei (n=3 for WT and n=5 for *dex30*). **(I-J)** mRNA expression levels of **(I)** *MADD*, **(J)** *CPE* at stages 6-7, relative to non-differentiated cells (n=4-6 for WT and n=6-9 for *dex30*). **(K)** Immunohistochemistry with insulin (red) and proinsulin (green)-specific antibodies in WT and *dex30* SC-islets. Scale bars 100 mm. **(L)** Quantification of proinsulin$^+$insulin$^+$ double positive (ProINSINS%), proinsulin$^+$ only (PROINS%) and insulin$^+$ only (INS%) as % of all insulin positive cells (n=3 for WT, n=5 for *dex30*). **(M)** Insulin content ng/ug of DNA in β-cells (n=7 for WT, n=8 for *dex30*). **(N)** Stimulation index (peak dynamic insulin secretion at G16.8/G2.8) in WT and *dex30* SC-islets (n=10 for WT, n=14 for *dex30*). **(O)** Dynamic insulin secretion in Stage 7 SC-islets as % of total insulin content in 2.8mM glucose, 16.8mM glucose, 16.8mM glucose+50mM Exendin 4 (Ex4) and 2.8 mM glucose+30mM KCL (n=7 for WT, n=9 for *dex30*). **(P)** Area under curve quantification of secretion curves in O. **(Q)** Detecting apoptosis in Thapsigargin-treated WT and *dex30* INS$^+$ cells at ST7 with terminal deoxynucleotidyl transferase-mediated dUTP nick end labeling (TUNEL) assay. Scale bars 100 μm. **(R)** Quantification of TUNEL-positive cells (n=3 for WT, n=5 for *dex30*). * $p<0.05$, ** $p<0.01$ analysed by Student's *t*-test (**B, M-N, P**) or multiple *t*-tests (**C-F, H-J, L, R**).

Supplemental Figure 5

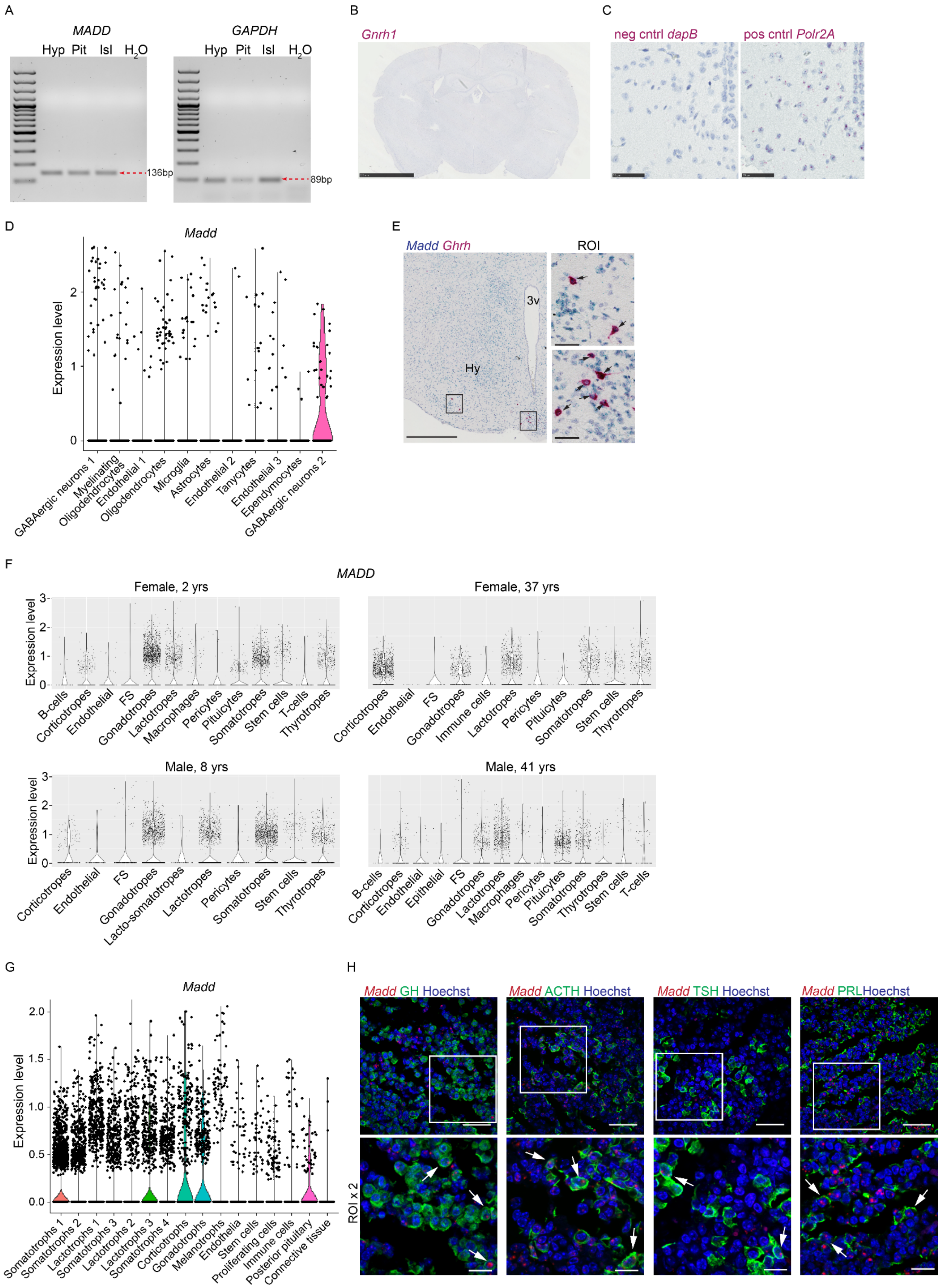

Figure S5. ***MADD*** **transcripts are expressed in hypothalamus and pituitary. (A)** *MADD* expression in human hypothalamic and pituitary cDNA libraries and cadaveric islet cDNA determined by RT-PCR. Hyp: hypothalamic cDNA, Pit: pituitary cDNA, Isl: islet cDNA. Expected sizes: *MADD*: 136 bp, *GAPDH*: 89 bp. The lanes with *MADD* and *GAPDH* RT-PCR products were run on the same gel but were non-contiguous. Lanes from the same gel are shown in Figure 2A. **(B)** RNAscope mRNA in situ hybridization using probe against *Gnrh1* (red) in adult mouse brain, coronal section shows absence of signal in caudal section. Scale bar 2.5 mm. **(C)** RNAscope mRNA in situ hybridizations using probes against bacterial gene *dapB* (red) and housekeeping gene *Polr2A* (red) as negative and positive controls, respectively. Scale bars 50 µm. **(D)** A violin plot showing expression of *Madd* transcripts in cell clusters identified from single-cell RNA-seq data from adult mouse hypothalamus. **(E)** A representative RNAscope mRNA in situ hybridization using probes against *Madd* (blue) and *Ghrh* (red) in adult mouse hypothalamus, coronal section. Arrows indicate examples of double-positive cells. 3v, 3rd ventricle, Hy, hypothalamus. Scale bar full size image 500 µm, ROIs 50 µm. **(F-G)** Violin plots showing expression of *MADD* transcripts in cell clusters identified from single-cell RNA-seq data from **(F)** pediatric and adult human pituitaries and **(G)** adult mouse pituitary. FS=Folliculostellate cells. **(H)** RNAscope RNA in situ hybridization of *Madd* (red) combined with immunostainings of different pituitary hormones (green) in adult mouse anterior pituitary. GH: growth hormone, ACTH: adrenocorticotropic hormone, TSH: thyroid-stimulating hormone, PRL: prolactin. Arrows indicate examples of double-positive cells. Scale bars: 50 µm; ROI 15 µm. RNAScope mRNA in situ hybridizations for *Gnrh1*+*Madd* were repeated >10 times, and for other hormones+*Madd* for 5-8 times.

Supplemental Figure 6

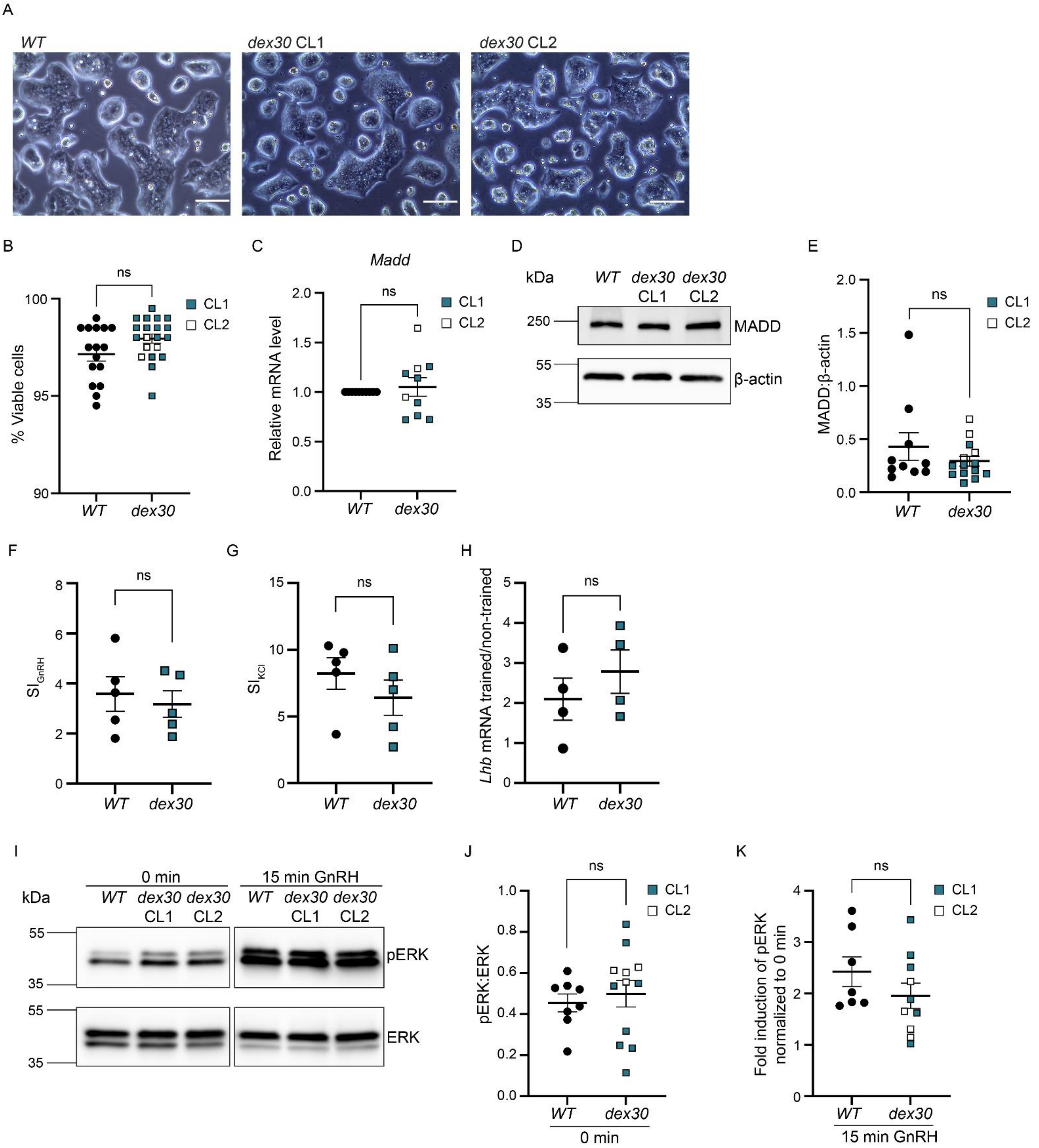

Figure S6. ***Dex30*** **LβT2 cells' morphology, viability and levels of** ***Madd*** **mRNA and protein are comparable to WT, and they retain functional secretion machinery and responsivity to GnRH. (A)** Light microscopy showing morphology of wild type LβT2 cells and two *dex30* clones. Scale bars 10 µm. **(B)** Viability of wild type and *dex30* LβT2 cells determined by Tryphan blue staining (n=16 for WT, n=20 for *dex30*). **(C)** Relative mRNA expression of *Madd* in wild type and *dex30* LβT2 cells (n=10). **(D)** A representative immunoblot showing MADD protein levels in wild type and *dex30* LβT2 cells. **(E)**

Quantification of MADD immunoblot band intensities normalized to β-actin in wild type and *dex30* LβT2 cells (n=10 for WT, n=14 for *dex30*). **(F-G)** Stimulation indices (ng LH secreted by stimuli/ng LH secreted spontaneously) of WT and *dex30* LβT2 cells stimulated with **(F)** 50 nM GnRH or **(G)** 60 mM KCl (n=5). **(H)** Relative increase of *Lhb* mRNA expression in WT LβT2 cells and *dex30* clone 1 after 3-days of training with pulsatile GnRH-stimulations (n=4). **(I)** A representative immunoblot showing phosphorylated and total ERK1/2 in WT and *dex30* LβT2 cells before and after 15 min stimulation with 50 nM GnRH. **(J)** Quantification of phospho-ERK1/2 band intensities before GnRH stimulation, normalized to total ERK1/2 (n=8 for WT, n=12 for *dex30*). **(K)** Fold induction of ERK1/2 phosphorylation after 15 min GnRH stimulation, normalized to total ERK1/2 (n=7 for WT, n=10 for *dex30*). ns $p \geq 0.05$, analyzed by Student's *t*-test.

Supplemental Figure 7

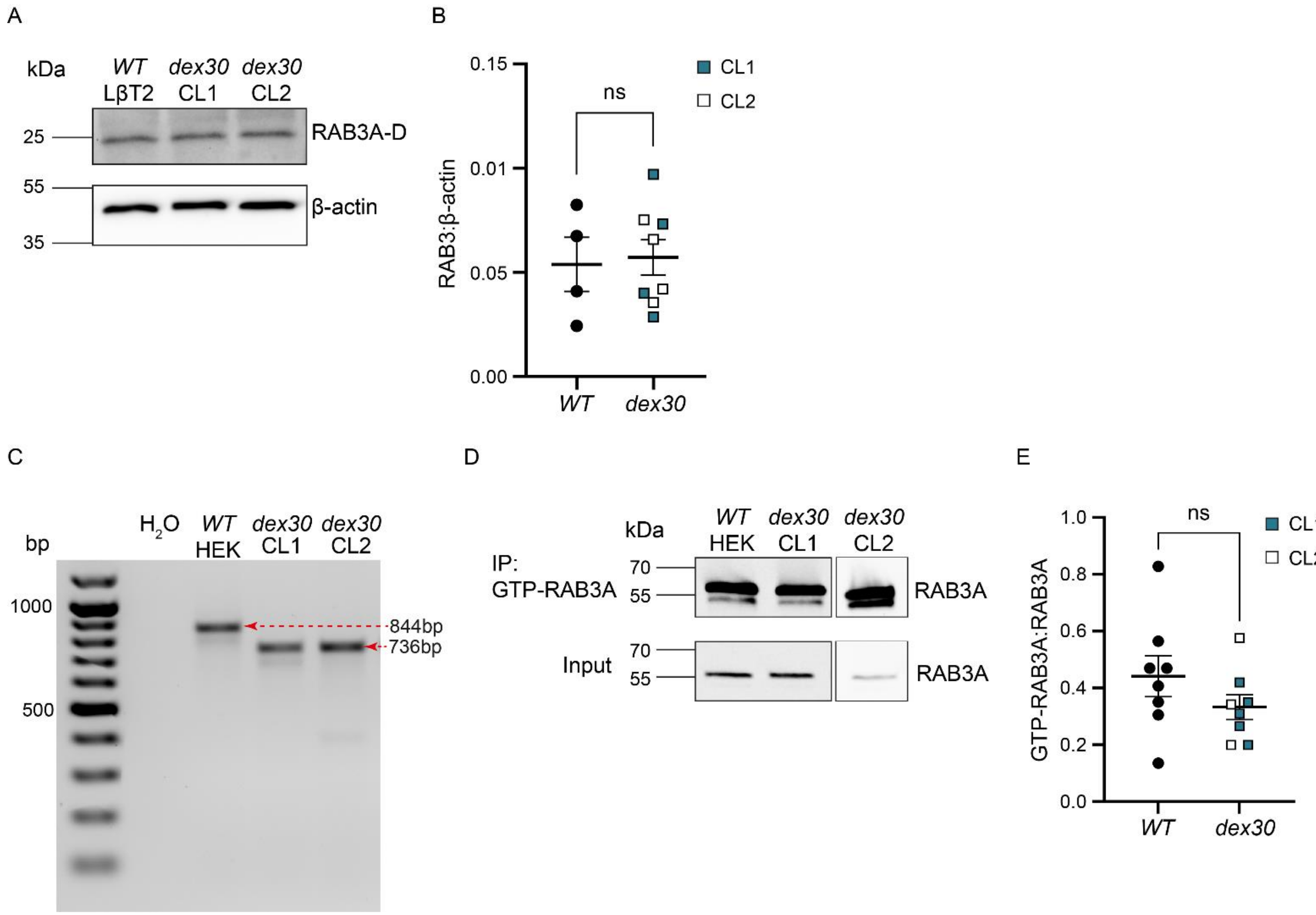

Figure S7. ***Dex30*** **does not affect stability or activation of RAB3 small GTPases. (A)** A representative immunoblot showing RAB3A-D protein expression in WT and *dex30* LβT2 cells. **(B)** Quantification of RAB3 immunoblot band intensities normalized to β-actin in wild type and *dex30* LβT2 cells (n=4). **(C)** A cDNA PCR with primers targeting around *MADD* exon 30 showing truncated transcript in two homozygous HEK293 clones. **(D)** A representative immunoblot showing activated GTP-bound (IP) and total (Input) RAB3A-EGFP transiently expressed in wild type and *dex30* HEK293-cells. **(E)** Quantification of band intensities of GTP-RAB3A in WT and *dex30* HEK293 cells, normalized to total RAB3A (n=8). ns $p \geq 0.05$, analysed by Student's t-test.

Supplemental Tables

Supplemental Table 1. Single nucleotide variants homozygous in the affected patients and heterozygous in their parents, with minor allele frequency (MAF) <1% in the 1000 Genomes database.

Gene name	Variant type	Transcript	Nucleotide change	Protein Change	Frequency (gnomAD)	Predicted impacts		
						BGI	SIFT	PolyPhen2
RASAL2	Silent	NM_170692.2	c.876T>A	p.(Ser292Ser)	0.0007548	Low	NA	NA
RASAL2	Silent	NM_170692.2	c.3420C>G	p.(Arg1140Arg)	0.0006719	Low	NA	NA
SLC1A2	Silent	NM_004171.3	c.1368C>T	p.(Ala456Ala)	0.001409	Low	NA	NA
ACCSL	Missense	NM_001031854.2	c.1630C>T	p.(Arg544Cys)	0.0009298	Moderate	deleterious	possibly damaging
TSPAN18	Missense	NM_130783.4	c.232C>T	p.(Arg78Cys)	0.00003548	Moderate	deleterious (low confidence)	probably damaging
MADD	Splice-site	NM_003682.4	c.4378T>G	p.(Asn1424_Glu1459del)	NA	High	NA	NA
RTN3	Missense	NM_001265589.1	c.2057C>T	p.(Thr686Ile)	0.000003988	Moderate	tolerated	benign
ESRRA	Silent	NM_001282450.1	c.1035C>T	p.(Ala345Ala)	0.00005391	Low	NA	NA
DPF2	Silent	NM_006268.4	c.924A>G	p.(Gln308Gln)	0.00009554	Low	NA	NA
TIGD3	Missense	NM_145719.2	c.475C>A	p.(Gln159Lys)	0.03107	Moderate	tolerated	benign
MAP3K11	Missense	NM_002419.3	c.2380C>T	p.(Pro794Ser)	NA	Moderate	deleterious (low confidence)	benign
LRP5	Silent	NM_002335.3	c.2445C>T	p.(Asp815Asp)	0.0005352	Low	NA	NA
IGHMBP2	Silent	NM_002180.2	c.726C>G	p.(Ala242Ala)	0.002477	Low	NA	NA
LTO1	Silent	NM_153451.2	c.135T>C	p.(His45His)	0.0006328	Low	NA	NA

Supplemental Table 2. In silico predictions of the effects of NM_003682.4 c.4377+2T>G variant on splicing

In silico tool	c.4377+2T (wild type) score	c.4377+2T>G variant score	Change	Settings
HSF matrices	89.9	63.06	-29.86%	consensus value (CV) threshold 65, variation threshold +/-10%
HSF MaxEntScan	9.65	2	-79.27%	CV threshold 3, variation threshold +/-30%
Splice AI delta[A]	NA	1.00	-1.00	Default
Pangolin delta[A]	NA	0.88	-0.88	Default
NNSplice	0.96	0.00	-0.96	Donor score cutoff 0.40
HAL Splice Prediction	99.0	0.00	-99.0	Wild type PSI 99.0
Spliceator	0.99	0.00	-0.99	Reliability: 98% Model: 200

[A]Splice AI and Pangolin delta scores describe the probability of splice site loss.

Supplemental Table 3. All high confidence interactors of wild type and *dex30* MADD.

See separate Excel file.

Supplemental Table 4. Significantly enriched GO biological processes in the interactome of *MADD*.

Cutt-offs: Fold enrichment > 2; False Discovery Rate (FDR) <0.05

GO biological process	Fold enrichment	FDR
GO:1900740 Positive regulation of protein insertion into mitochondrial membrane involved in apoptotic signaling pathway	116.8	6.15E-07
GO:0007165 Signal transduction	3.0	0.009486765
GO:0034613 Cellular protein localization	16.0	0.009486765
GO:0006605 Protein targeting	21.2	0.017668109
GO:0010737 Protein kinase A signaling	38.9	0.022142903
GO:0007188 Adenylate cyclase-modulating G-protein coupled receptor signaling pathway	16.5	0.031231133
GO:0043547 Positive regulation of GTPase activity	7.2	0.044522384
GO:0051726 Regulation of cell cycle	5.8	0.044522384

Supplemental Table 5. Up to 50 significantly over-represented pathways in the interactome of *MADD*

See separate Excel file.

Supplemental Table 6. Annotations of high confidence interacting partners of *MADD*

See separate Excel file.

Supplemental Table 7. List of high confidence interacting partners with significantly different relative abundances in protein complexes isolated from *dex30* and WT MADD expressing-cells

Members of the same protein family or subunits of the same protein complex are highlighted with the same color.

Gene	Full protein name	Detection method	p-value	Fold change *dex30*/WT
ALDH1A2	Retinal dehydrogenase 2	BioID	0.042812	0.731
ARHGEF7	Rho guanine nucleotide exchange factor 7	BioID	0.005868	0.000
BUB1B	Mitotic checkpoint serine/threonine-protein kinase BUB1 beta	BioID	0.002587	0.272
HGSNAT	Heparan-alpha-glucosaminide N-acetyltransferase	BioID	0.027691	0.345
HLA-E	HLA class I histocompatibility antigen, alpha chain E	AP-MS	0.007982	0.000
LRP4	Low-density lipoprotein receptor-related protein 4	BioID	8.72E-06	0.000
PROM1	Prominin-1	BioID	8.72E-06	0.000
RAPGEF6	Rap guanine nucleotide exchange factor 6	BioID	8.72E-06	0.000
STRN	Striatin	BioID	8.72E-06	0.000
SYT3	Synaptotagmin-3	AP-MS	0.042208	0.000
TAB1	TGF-beta-activated kinase 1 and MAP3K7-binding protein 1	BioID	3.17E-05	0.382
YWHAB	14-3-3 protein beta/alpha	AP-MS	0.000182	0.626
YWHAB	14-3-3 protein beta/alpha	BioID	0.038002	0.836
YWHAE	14-3-3 protein epsilon	AP-MS	0.002773	0.734
YWHAG	14-3-3 protein gamma	BioID	0.000413	0.758
YWHAG	14-3-3 protein gamma	AP-MS	0.012514	0.770
YWHAH	14-3-3 protein eta	AP-MS	0.033286	0.810
YWHAQ	14-3-3 protein theta	BioID	0.000615	0.624
YWHAQ	14-3-3 protein theta	AP-MS	0.017998	0.786
YWHAZ	14-3-3 protein zeta/delta	BioID	8.15E-05	0.674
YWHAZ	14-3-3 protein zeta/delta	AP-MS	0.000592	0.555

ARHGEF10	Rho guanine nucleotide exchange factor 10	BioID	0.036606	7.249
BAG5	BAG family molecular chaperone regulator 5	BioID	8.71E-05	→∞
FGFRL1	Fibroblast growth factor receptor-like 1	BioID	0.020032	8.457
FLG	Filaggrin	AP-MS	0.044523	5.328
GNAI1	Guanine nucleotide-binding protein G(i) subunit alpha-1	AP-MS	0.038618	5.328
GNAI3	Guanine nucleotide-binding protein G(k) subunit alpha	AP-MS	0.006312	2.291
GNB1	Guanine nucleotide-binding protein G(I)/G(S)/G(T) subunit beta-1	AP-MS	0.037612	4.186
GNB2	Guanine nucleotide-binding protein G(I)/G(S)/G(T) subunit beta-2	AP-MS	0.011858	6.660
PLAGL2	Zinc finger protein PLAGL2	AP-MS	0.030004	3.996
PPP4R2	Serine/threonine-protein phosphatase 4 regulatory subunit 2	BioID	0.001643	2.819
RAB21	Ras-related protein Rab-21	BioID	0.008996	→∞
RPAP3	RNA polymerase II-associated protein 3	BioID	0.002352	2.366
SLC25A10	Mitochondrial dicarboxylate carrier	AP-MS	0.01309	1.332
SSRP1	FACT complex subunit SSRP1	AP-MS	0.043881	4.440
STAT1	Signal transducer and activator of transcription 1-alpha/beta	BioID	0.011076	3.624
TMEM41B	Transmembrane protein 41B	BioID	0.047342	3.624
UNC45A	Protein unc-45 homolog A	BioID	0.033693	2.574
URB1	Nucleolar pre-ribosomal-associated protein 1	AP-MS	0.040351	6.660
USP9X	Probable ubiquitin carboxyl-terminal hydrolase FAF-X	AP-MS	4.8E-05	23.976

Supplemental Table 8. PCR primers used for amplification and sequencing of *MADD* transcripts

Amplicon number	Primers	Amplicon size
1	Fw: GCCCTGATGCTTCTCTGAGA<br>Rv: CGATGTCTACTCGCTTCTGC	848 bp
2	Fw: CTCCACCTTCCGAGAGTGTT<br>Rv: GATCTCCTGGCCCTCATGAA	805 bp
3	Fw: GCTAGTGGATCTGGACAGCA<br>Rv: AGCTCGTCAATCTCCACCTC	843 bp
4	Fw: TCCTCCCTTGGTGACTTTGT<br>Rv: TAACGCCCTCCTGTTTCCTT	636-825 bp[A]
5	Fw: CCCTTCCCCAGTCTGAAAGT<br>Rv: CCTCTGTCTCACCAAGGTCA	712-826 bp[A]
6	Fw: CACCAAGTGCCACAGGAAAG<br>Rv: GGCCAACAAGCGATCTTCAT	704-833 bp[A]
7	Fw: TGGAGAGAGAAGGGATGGGT<br>Rv: TGGGCCATTGGTGTCTTGTA	663-853 bp[A]
8	Fw: CTGAAGACTGGTGAGGGTGG<br>Rv: CACACGAGCATCATCCACTC	330-409 bp[A]

[A]Alternative splicing results in variable amplicon sizes

Supplemental Table 9. List of qPCR primers used

Gene	RefSeq/Source	Primers	Amplicon size
PPIG	NM_004792	Fw: TCTTGTCAATGGCCAACAGAG<br>Rv: GCCCATCTAAATGAGGAGTTG	84 bp
PDX1	NM_000209.3	Fw: AAGTCTACCAAAGCTCACGCG<br>Rv: CGTAGGCGCCGCCTGC	52 bp
NKX2-2	NM_002509.4	Fw: GAACCCCTTCTACGACAGCA<br>Rv: ACCGTGCAGGGAGTACTGAA	82 bp
NKX6-1	NM_006168	Fw: TATTCGTTGGGGATGACAGAG<br>Rv: TGGCCATCTCGGCAGCGTG	91 bp
SOX9	NM_000346	Fw: ATCAAGACGGAGCAGCTGAG<br>Rv: GGCTGTAGTGTGGGAGGTTG	100 bp
INS	NM_000207	Fw: CAGAAGCGTGGCATTGTGGA<br>Rv: GCTGCGTCTAGTTGCAGTAG	82 bp
PCSK1	KiCqStart®	Fw: 8810586217-10/0<br>Rv: 8810586217-10/1	-
PCSK2	KiCqStart®	Fw: 8810586217-20/0<br>Rv: 8810586217-20/1	-
MADD	NM_00137665 1.1	Fw: TCGAGGCGTACAAAGGGACAC<br>Rv: TACTGGGGAGCGCAGCAATC	133 bp
NGN3	NM_020999	Fw: GACGACGCGAAGCTCACCAA<br>Rv: TACAAGCTGTGGTCCGCTAT	98 bp
CPE	KiCqStart®	Fw: 8810819465-30/0<br>Rv: 8810819465-30/1	-
MAFA	NM_201589	Fw: GCCAGGTGGAGCAGCTGAA	77 bp

		Rv: CTTCTCGTATTTCTCCTTGTAC	
PreINS	NG_007114.1	Fw: GTGAACCAACACCTGTGCGG<br>Rv: AGGGGCAGCAATGGGCAGTT	139 bp
Lhb	NM_008497.2	Fw: CAGTCTGCATCACCTTCACCAC<br>Rv: CACACTGGCTGAGGCACAGG	100 bp
Madd	NM_00117771<br>9.1	Fw: GCTTATGGCGGAGAAATGGC<br>Rv: TCAGGCCCAGGTTTGATGC	136 bp
Tbp	NM_013684.3	Fw: TAAGAGAGCCACGGACAACTGC<br>Rv: AGTCTGGATTGTTCTTCACTCTTGG	85 bp

Supplemental Table 10. Antibodies used for flow cytometry (FC), immunocytochemistry (ICC), immunohistochemistry (IHC), and Immunoblotting (IB)

**Antibody**	**Supplier**	**Use and dilution**
Mouse anti-CD184 (CXCR4) PE Conjugated	BD Biosciences Cat# 555974	FC (1:10)
Mouse IgG2a, kappa Isotype Control, PE Conjugated	BD Biosciences Cat# 563023	FC (1:10)
Mouse anti-PDX1 PE Conjugated	BD Biosciences Cat# 562161	FC (1:80)
Mouse anti-NKX6-1 Alexa Fluor 647 Conjugated	BD Biosciences Cat# 563338	FC (1:80)
Mouse anti-NKX6-1, PE Conjugated	BD Biosciences Cat# 555574	FC (1:80)
Rabbit anti-Insulin (C27C9) Antibody Alexa Fluor 647 Conjugated	Cell Signaling Technology Cat# 9008	FC (1:80)
Mouse anti-C-Peptide Alexa Fluor 647 Conjugated	BD Biosciences Cat# 565831	FC (1:80)
Rabbit IgG Isotype Control Alexa Fluor 647 Conjugate	Cell SignalingTechnology Cat# 3452S	FC (1:80)
Mouse IgG1, kappa Isotype Control, PE Conjugated	BD Biosciences Cat# 555749	FC (1:80)
Guinea pig anti-INS	Dako Cat# A0564	IHC (1:500)
Mouse anti-PROINS	DSHB Cat# GS-9A8	IHC (1:300)
Mouse anti-GCG	Sigma-Aldrich Cat# G2654	FC (1:80), IHC (1:500)
Alexa FluoR 488 Donkey anti-Mouse IgG secondary ab	ThermoFisher Scientific Cat# A-21202	IHC (1:500)
Alexa FluoR 594 Goat anti-Guinea Pig IgG secondary ab	ThermoFisher Scientific Cat# A-11076	IHC (1:500)
Alexa FluoR 488 Donkey anti-Mouse IgG secondary ab	ThermoFisher Scientific Cat# A-21203	IHC (1:500)
Rabbit anti-LH	National Hormone and Pituitary Program (NHPP) Cat# AFPC697071P	IHC (1:1000)
Rabbit anti-FSH	NHPP Cat# AFPHFSHb	IHC (1:1000)
Rabbit anti-GH	NHPP Cat# AFP-5641801	IHC (1:1000)

Mouse anti-ACTH	Fitzgerald Cat# 10C-CR1096M1	IHC (1:1000)
Rabbit anti-TSH	NHPP Cat# AFP-1274789	IHC (1:1000)
Rabbit anti-PRL	NHPP Cat# AFP-4251091	IHC (1:1000)
Alexa Fluor 488 Goat anti-Rabbit IgG secondary ab	Invitrogen Cat# A-11008	IHC (1:500)
Alexa Fluor 488 Goat Anti-Mouse IgG secondary ab	Abcam Cat# ab150113	IHC (1:500)
Rabbit anti-GnRH	Immunostar Cat# 20075	ICC (1:1000)
Mouse anti-Tuj1	Sigma Cat# T8578	ICC (1:1000)
Alexa Fluor 488 Donkey anti-Rabbit IgG secondary ab	Invitrogen Cat# A21206	ICC (1:500)
Alexa Fluor 594 Donkey anti-Mouse IgG secondary ab	Invitrogen Cat# A21203	ICC (1:500)
Rabbit anti-MADD/ DENN clone EPR4919	Abcam Cat# ab134117	IB (1:1000)
Mouse anti-RAB3(A-D)	Synaptic Systems Cat# 107 011 Clone 42.1	IB (1:1000)
Mouse anti-β-Actin	Santa Cruz Cat# sc-47778	IB (1:1000)
Rabbit anti- p44/42 MAPK ERK1/2	Cell Signaling Technology Cat# 9102	IB (1:1000)
Rabbit anti-phospho-p44/42 MAPK ERK1/2 (Thr202/ Tyr204)	Cell Signaling Technology Cat# 9101	IB (1:1000)
Goat anti-Rabbit IgG-HRP conjugate	Bio-Rad Cat# 1706515	IB (1:3000)
Goat anti-Mouse IgG-HRP conjugate	Bio-Rad Cat# 1706516	IB (1:3000)
Mouse anti-RAB3-GTP	NewEast Biosciences Cat# 26920	IP (1:1000)
Rabbit anti-RAB3A	NewEast Biosciences Cat# 21041	IB (1:1000)

Supplemental Methods

Minigene assay

Strategy for generating inserts containing *MADD* exons 29, 30 and 31 and parts of the surrounding intronic sequences is presented below.

Genomic DNA sequence GRCh38:11:47311701-47323867

Intronic sequence included in the insert
Exons 29, 30 and 31
Intronic sequence excluded from the insert
AG/GT Acceptor site/Donor site
c.4377+2 T>G

CCTTGTTAAGAGTCATGTGTTGGCTTTTC**AGGTAAATAAGAATGACATCCGCAAGAAGGTGAGGCGCCTAATGG**
GAAAGTCGCACATTGGGCTTGTGTACAGCCAGCAAATCAATGAGGTGCTTGATCAGCTGGCGAACCTGGTAAGC
ACGTCTGGCCACCCCTTAGGCTTCCCCATGGGTCATTTCTTGGTTTGTGTCACTTGCAGTCCAGTTCACCCCGTT
TTTGAAAATGGAGCAGTTGTCTTTGACTGTAAATGAGGCATTAGTCCCTGTGTTCTGTGATGGGATTCTCTGTAT
AAAACCATCGTAGTGCATCAGTTTTGATGTGTGGTCTGTGAACTGCTGAGGGTTCAGAAGGTGAAAACGATTTCA
AATGATTCTAAAATGTTATTTGCTGTTTTCACGGGGTTACATTTGCACTGATGGTACCAACGCAGTGATGGGTAA
AACTGCTGGCACTGTAACCCAAGTGAAGACAATGGCCCCAAACTATGCCAGCAGCCATTGCATTCCTTGCCTTCA
TATACTCTCAGTTTTTCGTTTTTGTTTTGTTTGTTTGTTTTTTGAGACTGACTCTCACTCTGTCGCCCAGGCTGG
AGTGCAGTAGCGCAATCTTGGCTCACTGCAACCTCCACCTCCTGGGTTCAAGCGATTCTCATGCCTCAGCCTCCC
AAGTAGCTGGGATTACAGGCATGTGCCCGCCCACCCAGCTAATTTTTGTATATACATATATATTTTTTGAGACAG
AGTTTCACTCTCTTGTTGCCCAGACTGGAGTGCAATGGCGCAATTTTGGCTCACTGCAACCTCTGCCTCCCGAGT
TCAAGCAGTTTTCCTGCTTCAGCCTCCCGAGTAGCTGAAGCTCCTGAGGTTACAGGCATGCACCACCACGCCCAG
CTAATTTTTGTATTTTCAGTAGAGATGGGCATTCCCCATGTTGGCCAGGCTGATCTTGAACTCCTGACCTCAAGT
GATCTGCCTGCCTTAGCTCCCAAAGTTCTGGGATTACGGGTGTGAACCACCGCACCTGGCTTACACTCTTAGTTT
TTTAAAAATGCCAGTTGTGCTTGTAAATGTCCTTGAAGAAGCAGTGAAGACTGTTACTCTTATTAGATTGATTAA
TTATAAATGTTTTTAATATTCTTTGTGACAAAATGGGAAGTACACATAAAGCATCTCTGTTGCAAACCCAGTATG
ATGGTTGTTTTGAAGAAAAGCAATTTGCAGCCTTGATTTGTTCACCTTGAGTTGCAAGTGAACTTAATCGCTTTG
TTCATTGAACATCATTTTTACTGGAAAGGACAATTAATAAACCATGGTTATTCAGAGTACCCAACTGGCAGACAT
TTTCCTTCAGTTAATCAAGTGAGCCTGTTGTTTCAGGGAAAACAACTGACAGTATTTGTTGCCAATGATAAGATT
CAGCTTTTAAGTGAAAATAAGAATTTTGGCAGAGTTTTGAACTTGAAGCTTCCCAATATTTAAAGACTTTTCTAA
TGAGATTGGTGGTGATATTAATGAATGTGATTTTTTAAACATTGATTTAATATTACAAAATATGTCAACATTTGG
AAGAGCTGTGTAACTCAGTGAAACACTATTTTCTTTCTTTTTTTTTTTTTGGAGACGGAGTCTCGCTCTGTGGCC
CAGGCTGGAGTGCAGTGGCAGGATGCTGGCTCACTGCTACCTCCGCCTCCTGGGCTCCTGGGTTCAAGCAATTCT
CCTGCCTCAGCCTCCTGAGCAGCTGGGAGTACAGGCGTATGCTGCCACACCTGGCTAGTTTTTTTTTTTTTTTGT
ATTTTAATAGAGACGGGGTTTCACCATCTTGCCCAGGCTGTGGTCTTGAACTCCTGAGTTTAGTCAATCCGCCCA
CCTTGGCCTTCCAAAGTTCTAGGATTACAGGCACCCAGCCAACAGTATTTTCTAAATACCAATGTATGATGTTGT
AAATTCAAGTATGGATAAAAGATCTACTCAAAATGCAAGGCAGAGATATGGATCTTAATTTTTAAATAACATTTT
TTTTCAGTCTGGGGAACATAGACCCCATTTCTTTTTGTTTGTTTGTTTGTTTTTGAGACGGAGTCTCACTCTGTC
ACCCGGGCTGGAGTGCAGCGGCGCGATCTCGGCTCACTACAACCTCCGCCTCCCAGGTTCAAGCTATTCTCCTGC
CTCAGCCTCATGAGTAGCTGGGATTACAGGTGCCCGCCACAACGCCCGGCTAATTTTTATGTTTTTAGTAGAGAT
GGGGTTTCACCACGCTGGTCAGGCTGGTCTCCAACTCCTGACCTTTTGATCCGCCCACCTCGGCCTCCCAAAGTG
CTGGGATTACAGGTGTGAGCCACCGCGCCCAGCCAAGGGAGACCCCATTTCTACAAAAAATAAAAAAATTAGCTA
GGCATGGTGGCATGCACCTGTAGTCCCAGCTACTTGGGAGGCTGAGGTGGGAGGATCACTTGAGCTCAGGAGGTT
GAGGCTGCCGTGAGCACCACTGCACTCCAGCCTGGGTGACAGAATGAGACCCTGTCTCAAAAAAAAAAAAAAAGT
GTATGGAGAATAGGGTCTCACTTTGTTGCCCAGGCTGGTCTCCAACTCCTGCCTGGGCTCAAACGGTACTCCTGA
CTTGGCCTCCCAAAGTGTTGGGATTATAGGAATGAGCCACCATGTCTGGCCTAAATGGATTTTAATGTAACAATG
TAAAATGTTCATTGATGTGCTTTCAGATTCCACACTGCAACTAACTTTCAAAAGCTGCTTTTGCTGGGTGTTGTG
GTTTATGCCTGTAATTCCAGGTACTTGGGAGGCTGAGGCAGGAGTGTCACTTAAGCCCAGGAGTTCAGAGCTGTG
GTGGGCTATGGTCATGCCACTGCACTCCAGCCTGGATGACAGAGCGAAACCTTGTCTCTAAAAAAAAAGAAGCTT
CTGCTTCCCAAGTTCTGGTGTAGTGTTAAAGAAGAATACGTCTATTTATCTGAAAAGGCTATGAAAATCCTCTTC
CATTTTCCAAATACGTATCTCTGAGGTCAAGTTTTTTTCATATACCTCAACCAAAGCAACATACTGCAACAGACT
CAATGCAGAGGCAGATAGGAGAATGCAACTATTTGATTCTAAGCCAAACATTAAAGAGATTTGCAAAAATGTGAA
ACAGTGCCTTTTTTTTGAAAATATAGTTATTTTATTAAAAATGTTTATATTACTATGTAATGGGTTATATTAATC

ATTAATTGTTTTTATAATTAATCATTTGAGAAATTTCTGTTTAAATTTCTAGTATGTAAATATCTATAAATATAA
CTCACATAAACAAAAGCTCTTTGGAATCTTCAATAAATTTTAAGAGATATAGGGCCTCTGAGACCAAAACATCTG
AAACCACTTCCCTGGTGACCAGTGGCCAGCAGATGAGACTGTGCTGAGGAAGCCGATATGAATTTGATTGCTGGA
TGGGGAATTCTTGGCCCAGAGCCCTCTGAGAGGGATGTATGACTGTCCCTAAAAAATCTCTCTTTCATC**AGAAT**
GGACGCGATCTCTCTATCTGGTCCAGTGGCAGCCGGCACATGAAGAAGCAGACATTTGTGGTACATGCAGGGACA
GATACAAACGGAGATATCTTTTTCATGGAGGTAGGTGCTGGTTCATGCTGGGGCCCAAAGGGCTATTGAGAGT
CACAGGGAACTCATAGGACCGATGCCAGGATATTTTTCTCATTTATCTCATTCATCTTCATGATCTATTTATCAT
TAAATTATCAGATTGGTTTTTTGTTTTTTGCGTGTGTGTTTTTTTGAGATAGTGTCTCGCTCTGTCACCCAGGC
TGGAGTGTAGTGGCTTGATCTCGGCTCACTGCAACCTCCGCCTCCCGGGTTCAAGCAGTTCTCCTGCCACAGCCT
CCCAAGTAGCTGGGATTACAGGCGCCAGCCACCAAGCCCAGGTGATTTTCTATTTTTAATAGAGATGGGGTTTG
ATCATGTTGGCCAGGCTGGTCTCAAACTCCTGACCTCAAGTAATCCACCCACCTTGGCCTTCCAAAATGCTGGTA
TTATAGGCATGAGCCACCGCACCCGGCCAAATTATCAGATTGTTATTCAAATAATTAAATTTTATATAGTAGATG
ATACCCATTTCTTTTTTTTTTTTTTTTTGAGACAGAGTATCGCTCTGTCACCCAGGCTGGAGTGCAGTGGTGTGA
TCTTGGCTCACTGCAGCCTCCACCTCCCAGGTTCAAGCAATTCTCCTGCCTCAGCTTCCTGAGTAGCTAGGATTA
CAGGCATGCGCCACCATGCCCGGCCAATTTTTTGTATTTTAGTAGAGGTGGGGTTTCACCATGTTCGTTGGCCAG
GCTGGTCTTGAACTCCTGACCTCAAGTGATCCACCTGCCTCGACCTCCCAAAGTGCTGGGATTACAGGTGTGAGC
CACTGCGCCCGGCCCCATTTCTTTTATACACGTGCGCGCGCACACACACGCGCACACACATATACATAAAGATAT
CTATATATAGATGATATAGTCACATTGTTCATAAATCAGAATAACAGAAAATAGTACACATTGGGAGGTCTTGCA
CTCATTCTTGTCCCCTTCCACTGAATTTCCCACTCCCCTCACCCCTTCATCCTATAGACAACCACTTTTATTGGT
TTCCTTTGTAAATTCCAGTGGATTTTTTTTTTCTTTTTCTTTTTTTTTTTTTTTTTTTTGAGACGGAGTCTCAC
TCTGTCGCCCAGGCTGGAGTGCAATGGCTTGATCTCAGCTCACTGCAACCTCCACCTCCTGGGTTCAAGCGATTC
TCCTGCCTCAGGCTCCCAAGTAGCTGGGATTACAGGCGCCCACCACCGCACCCAGCTAATTTTTTGTATTTTTAG
TAGGGATGGGGTTTCACCATGTTGGTCAGGCTGGTCTCCAACTCCTGACCTCACTGATCCACCCGCCTCGGCCTC
CCAAAGTGCTGAGATCATAGGCGTGAGCCGGTGCACCCGGCCTTCCAGTGGACTTTTATGCAAACAATAAAATAT
GAACACAAATTCTTATTTTCCTCCTTTCTTTTTTTTCTTTGAGACAGAGTGTTGCTCTTTTGCTCAGGCTGGAGT
GCAGTGGTCCAAACTCAGCTCACTGCAACCTCTGCCCCTTGGGTTCAAGTGATTCTCCTGCCTCAGCTGCCTGAG
TAGCTGGGATTACAGTCGCCCACCACCATGCCCAGATAATTTTTCTATTTTTAGTAGAGACGGGGCTTCACCATA
TTGGCCAGGCTGGTCTCAAACTCCTGACCTCAAGTGATCTGCCCACCTTAGCCTCACAAAGTGCTGGGATTATAG
GCGTGAGCCACCCCGCCTGGCCTATTTTTCTCCTTTCTTTCAGAAAGATAGCACATCTTATCCATTGCTTTATCC
CTTGCTTTTTTCATTTAACATTGTCCTGGTCCTCTTCTGTATCAGTACCAAGAAAGCTTTCCCATTGGTTTTATG
GCTACATGTATTCCACTGTGTGACTGTGCCTTAATTTATTTAACCAGTCAGTACATATGGATCCTTGGGTTGCTT
CCAAATTTGCTGTTACTGATAGTCATACTGTGAATGATGCTGTGCCTGCTTCATTTCATACATGTGCAAGTGTGT
CTAAGGATTCCCAGAAATGAGATTGCTGGGTCAGAGGGACAATGCATTTGTAATTTTGGTTGATATAATTCTTAA
GTGTCTCATAACACGTAATACCCCCTTTGCCATTAATTTTGTTGAAGAAATTGGATCGTTTGCCCTGTGGAGTCT
CCTACATTCTAGATTTTTTGCTGATTGTGTCTCCTTTGTATTAGCGTGTTTCCTGTCACATATTTCTCATAGACT
GGTAGTTCAGTTTAGAGGTTTGATCACTTTCAGATTCACTCTTTTGGGAAGAATACTTAATGAGTGGTGCTATAC
GCTTCCTATTGCCTCATATGGTTGTCCCTCTCTTACACATCAGTGGGCTCAGATGTGGGCAACCTGATCTGCCCA
CCAGAAAGTTCCTCATTTGTCTTCTGATTTTGCCTATGGTATTTTTTGACACACATAAATTTTTTATTTGTATAT
AACTTTTTTTTTTTTTTGAGACAGAGTCTCTCTCTATCGCCCAGGCTGGATGCAGTGGCATGATCTCGGCTCACT
GCAACCTATACCTCCCGGGTTCAAGCAGTTCTCCTGCCTCAGCCTCCCAAGTAGCTGGGTTTACAGGTGCCTGCC
ACCACACCCGGCTAATTTTTGTGTTTTTAGTAGAGATGGGGTTTTACCATGTTGGCCAGGGTGGTCTCGCACTCC
TGACCTCAAGTGATCCACCCACCTCGGCCTCCCAAAGTGCTGGGATTACAGGTGTGAGCCATCACGCCTGGCTAC
TTTTATATAATTAAATTAATCAATAGTTTCTTTTACGGCTTCTGGATTTTGAGGCATGGTTAGAAAAAGCCTTAC
TCTCCAGGATTATAAAGGAAATTGGCCGTGTTTTCTTCATTTCTGTGTTTTTAATTCTGAAACAGACCTAAGTAA
TTTTAAGGACCAAATACAACTCCTATATATAAAGTAGTAAAATTTTGCCTATGTTATCTGTTCCCCTGTGGTTTA
GCTGAATTTGAATATCACATTCACAGTTTTTTTGAACAGGTGGTCTACCAAACGCAAGAAGGGCTTACCCTGAAG
CAGAAACATACATGTCAGTGTAGCCAAGAAGGAGTCATTTTCATGTCACCCTTTAAAGTGTTAGAATTTGGCTAG
TTGCCTCTCAGAGCCTAGGGCTGACTTAGGACATCTGCACCACAGCAGAAGTGGCACATGACTCTTGCCCTTTTC
TTTTCCTTTTAAAAAAAATATCTCCAGCAGATTCCTTGAACTACTCTTACTCTCACCACCGTACAAAGGGAGCCT
CATGCTAAGTCTCTGGAGTGTGGGACTGGACCCATTACCCAAATAGATGCCTCTTCCTCGAAGTGGTGCCATGCC
AGGGAAGAGTGCACCATGGTCACTGTGCCATCACCTGGCTCCTGTGAGGCTATTTGATTTAGCCTTTCTCAGAGC
ATAGCCTGCTTTTTGCATGATCAACTGGACAGCAGTTTGGGAATTTTTTTTTTTTTTTTTTTTTTTTTTTTTTTT
TTTTGAGACAGAGTCTCACTCTGTTGCCCAGGCTGAAGTGCAGTGGTACAATCTTGGTTCACTGCAACCTCCACT
TCCCAGGTTCAAGCGATTCTCCTGCCTCAGCCTCCTGAGTAGCTGGGACTACAGGCACATGCCATCATGTCTGGC
TAATTTTTGTATTTGTAGTAGAGACGGGGTTTCACCATTTTGGCCAAGCTGGTCTCGAACTCCTGACCTCAAGTG
ATCTGCCCACCTCAGCCTCCCAAAGTGCTGGGATTACAGGTGTGAGCTGCCACACCTGGCCAAGGAAAATATATA
TATATATATAATTTTTTTTTTTTTTTTGGAGGTGGAGTCTTGCTCTGTCACCCAGGCTGGAGTGCAGTGGCACAA
TCTTGGCTCACTGCAACATCCACCCACCGACTTCAAGAGATTCTCCTACCTCAGACTCCCGAGTAGCTGGGACTA
CAGGCACGCATCACCACGCTTGGCTAATTTTTGGTATTTTTAGCAGAGATGGGGTTTGCCATGTTGGCCAGGAT
GGTCTTGAACTCCTGACCTTAAGTGATCCGTCTGCCTTGGCCTCCCAAAATGCTAGGATTACAGGTGTGAGCCAC

CATACCCAGCCAAAGATTTTTAAAAATTGTCTTTGTTGCCCTTTTTTTAGTTACAGAAGTAAAACAGTATATGCT
GTAAATGTTTCAAACCGTGCCAAAGTTAAAAAATAATTAAAAGTTTTACTCACTGCCTTTCAATCCTTTGTCCCA
GAGGTAACCAGTAATGAAGAGTTTTATGTGACTCCTTTCAGATTATTGAAAATGCATATTTGTGCAAATATAATT
TATTTATATGGAATTTTGTTTTTGCACTTACGAAAGTGGTATCAAATTACAAATATTGGGCTGGGCATGGTGGCT
CACATGAGCCTGTAATCCCAGCACATTGGGAGGCCGAGGCAGGTGGATAACTTGAGGTCAGGAGTTCCAGACAAG
CCTAGCCAACGTGGTGACACCCTGTTTCTACTAAAAATACAAAAATTAGCCGGGCGTGTTGGGCAGGCGCCTGCA
ATCCCAGCTACATGGGAGGCTGAGGCAGGAGAATCGCTTGAACCCGGAGGCGGAGGTTGCAGTGAGCCGAGATCA
TGCCACTGCTCTCCAGCCTGGGTGACAAAGCGAGACTCCATCGCAAAAAAAAAAAAAAAAAAAAAATTACAAATA
TTGTTAAATAATTTGCTTTTTTCACTTAATGATATACCTTTCCGTGTCTTTTTTTTTTTTTCTTTAATGGATGCT
TAGCCATTAGATGTCGTATGGTGTACCATAATTGGTTTAACTAGTGCCTCTCACTATTTTTTAAAAAAATATTAC
TCATGCTGGGTGTGGTGGCTCACGCCTGTAATCCCAGCACTTTGGGAGGCCAAGGTGGGTGGATCACGAGGTCAA
GAGTTCAAGATCAGCCTGGCCAAGATGGTGAAACCCTGCCTCTACTAAAAATACAAAAAATTAGCCAGGCGAGGT
GGTGGGTGCTTGTAATCCCAGTTACTTGGGAGGCTGAGGCAGAGAATTGTTTGAACCTGGAGGCGGAGGTTGCA
GTGAGCCGAGATTGCACCACTTCACTCCAGCCTGGGCGACAGAGCAAGACTCCGTCTCAAAAAAAAAATTTAAAA
AAATGAAATATATATTTATATATTACTCATTAGGCCAAGCATGTTGGCTCACGCCTGTAATCCCAGTACTTTGGG
AGGCTGAGGCAGGAGGATTGCTTGAGCCCAGAGTTTGAGACCAGCCTGGGCAACATAGGGAGACCCTGTCTCTAT
AAAAAATTTAAAAATTAGTCATACATGTGGTGATGTATGCCTGTGGTACCAGCTACTGGGGAGGCTGAGGTGGGA
GGTTCGCTTGAGCCCAGGAGGTCAAAACCAGCCTGGGCAACATAGGGAAACCCCGTCTCCACAAAAAATTTTAAA
ATAAGCTAGGCATGCTAGTGCATGCCTATGGTCCTAGCTACTGGGGAGGCTAAGTTGGGAGGATCCTTGAGCCTG
GGAGGTCAAGGCTTCAGTGAGCTCTGATTACACCACTTCAGTCCATCCTGGGTGACAGAGCAAAACCCTGTCTCA
AAAAATAATAATAATAATAATAATTGCTCATTGAATATCCTTGAACATATATTTTTGCACCCTCACATGAGAATT
TCTATAGGATATAGTCCTAGACATGAAAATTTCAAGTCAAGCTTATATACGTTTAAAATTTCGGTAGATTTTGCC
AAATTGCCATCCCAAAAACTATACCAGTTTACACCTCCAACAGCCACTTAGACGACTATAACAGTATATTACAAG
CATTTCTCTAGACTAGAATGCTCTAGCTTAGAAATCATGTGTTCTCCTAAATGTGTTCTGCCTGCCTTTACATGA
TGTTCTTAAAGGATTATGAAAGGTGGAGTGGTATTGTCCATTTTCCCCCTGGAGATAACCACAGAGTCAGACTGG
GCCTAGAACTCAAGTTTCCCATTCCTACTCTGCCACTTTGACTGATTCTTCAAAGGCTTTAGAACTCCAGTAAAG
TCTTATACAGTAGCTTAAACCTGATAGTCTTGTGTGTTCCTTACTCACTCACCTAATATTTGTGGAGCACGTATT
ATATGCTAGGTACTTTGTTGGGTGTTGGGATTATAGCAGTGAATAAGACAGACGTGATTTCTATTGCCCTTAGCA
TTGACAAGCTATTGGGGAAATCAGATAATTAAAGAGTATTATAAAATATGGTGAGCATTATGAAAGAAGCTCAGG
GGGGGCAGAATAAATACCAGAGGGCCTAGGAGCTGTGGACAACTCCTTTGAGAAGTTGACCTTTGAGCTTAGACT
TGAAGAAAGAGTAAGAGTTAATGGTTGGGGAGTGGAAGAGCAGTCAGGGATATTTGAGCTAGAACCAATGGGACT
CAGTGATGGAGTGTAGTTAGTTAGAGGGAAGAGGAAGGAGTCGGGCATCAATTCTTCATTTCAGGCTGGGGCAGC
TGGGTGGATGGTGGTGCCATTTCCTCAAATAGCAGACTTAAAAGGAGGATCTGCCCAGGAGGAGATGTTGAGTTC
AGATTTGGACACAATGAGTTTGAGGTGCTACATGTGAAGCATCCAGACACTGAGAAGCTCAGGAGCTCAGGAAGA
GAGGTCTAAGATGGAGAAAGGTAAGAACCATCAACATGGTAATAATCATTAGTATCATAGTTCTGATATCTAGTA
GCTAATTAGGGTACTTTCTGTGTGCCTGGCTTTGTGTGCACCTTAAATACTTTATGCACATTAACTCATTTAGTC
TTCATAGTAACTGTAGGAAGTTATTACTGTTACTATCCCCATCTTACCTGTGAGGAATTGTGGACACAGATGAAC
TTGCTCAGGGTGGCACAGCTCAGTAATGGCAGAGCTGGAATCTAAACTCAGGCTGACTTTAGACCTGTGCCCTTC
ACCATTGTGCTGTAATGTCTCCCGTAACATTTGAAGCCTTAAGAACGGATAACTGGCTGCGCATGGTGGCTCACG
TCTGTAATCCCAGCACTTTGGGAGGCCGAGGCGGGCGGATCATGAAGTCAGGAGATCAAGACTATCCTTGCTAAC
ACGGTGAAACCCCGTCTCTACTTAAAAAAATATATAAAAAATTAGCCGGGCATGGTGGTGGGTGCCTGTAGTCCC
AGCTACTCGAGAGGCTGAGGCAGGAGAATGGCGTGAACCTGGGAGGCGGAGCTTGCAGTGAGACGGGATTGCGCC
ACTGCACTCCAGCCTGGGCGACAGAGCGAGATCCGTCTCAAAAAGAAGAACAGATAACTTCACCTGAAAGAGAAT
GTTGAACAAGAAGGTGTCAAAGATATTTGCTTTTTAATTTATATCATCCTGTATTGTATCATTTTTATGTAATAA
GCATTGTTCATGTGTCACTTGTGTAATTAATTTTTAAAAATATTTTTATAGAAAAAGGGTAGACCCAGGATTAGA
AAGTGCATCAGCTCCATTTTTCTCCTTGGTTAAGTCTGTCATGATCTCCTACCTTTGTGTTTGTCAACAAAGATG
ATTCCTGAGCTTGTAAGATGGAGGCATGAGATCTCCTCCAAATACTTTAAGTCCGTAGAGCACATAAGAGGTAAT
AAATAACTGGGCCGGGCGCGGTGGCTCACGCCTGTAATCCCAGCACTTTGGGAGGCCAAGACGGGTGTATCACTT
GAGGTCAGGAGTTCGAGACCAGCCTGGCCAACATGGTGAAACCCTGTCTCTACTAAAAATACAAAAATTAGCTGA
GCATGGTGATGTGTGCCTGTAATCTCAGCTACTCGGGAGGCTGAGGCAGGAGAATCATTTAACCCACAAGGCAG
AGGTTGCAGTGAGTCGAGATTGCACCACTGCACTCCAGCCTGGGCAACAGAGTGGGACTCCATCTCAAAAAAAAA
AAAAAAAAATTATAGCCAATGTGATGGTGTGCACCTGTAGTCCCTGCTACTTTGGGGGCTGAGGCAGGAAGAACC
TGGCTGGTCGAGGCTGCAGTGAGCTGAGATGGCGCCACTGCAGTCAAGCCTGGGGTACAAAGTGAGACCCTGTCT
CAAAAAAAAAAAAAAGAGGTAATAAATAACTGATTGATTTGTGCCTCCTCCCTTATACATCGGTTCCTTCTAATG
ACTCCATCTCTTCCAACAATCCTTGTAGCAGGCAGCATGTCAGTAGTTCTGTTTTGTGTCTTCACCTAGGGAAAC
CATGAGCGTAAGGCAGAAAACCTGACCATGGGGATGCCCCAGGACCACCTTTCCCTGTAGGAAACAGTCTAGGGA
GAGGCTGTCAGAGACAGGAACCACTGAGCCGCTTTGTGCACTTCTCC**AG****GTGTGCGATGACTGTGTGGTGTTGC**
GTAGTAACATCGGAACAGTGTATGAGCGCTGGTGGTACGAGAAGCTCATCAACATGACCTACTGTCCCAAGACGA
AGGTGTTGTGCTTGTGGCGTAGAAATGGCTCTGAGACCCAGCTCAACAAGTTCTATACTAAAAAG**GT**ACGCAGG
ATCTGTGTTTGGGTTGGGGCTAG

Final T-construct (wild type) insert sequence:

CCTTGTTAAGAGTCATGTGTTGGCTTTTC**AGGTAAATAAGAATGACATCCGCAAGAAGGTGAGGCGCCTAATGG**
GAAAGTCGCACATTGGGCTTGTGTACAGCCAGCAAATCAATGAGGTGCTTGATCAGCTGGCGAACCTGGTAAGC
ACGTCTGGCCACCCCTTAGGCTTCCCCATGGGTCATTTCTTGGTTTGTGTCACTTGCAGTCCAGTTCACCCCGTT
TTTGAAAATGGAGCAGTTGTCTTTGACTGTAAATGAGGCATTAGTCCCTGTGTTCTGTGATGGGATTCTCTGTAT
AAAACCATCGTAGTGCATCAGTTTTGATGTGTGGTCTGTGAACTGCTGAGGGTTCAGAAGGTGAAAACGATTTCA
AATGATTCTAAAATGTTATTTGCTGTTTTCACGGGGTTACATTTGCACTGATGGTACCAACGCAGTGATGGGTAA
AACTGCTGGCACTGTAACCCAAGTGAAGACAATGGCCCCAAACTATGCCAGCAGCCATTGCATTCCTTGCCTTCA
TATACTCTCAGTTTTTCGTTTTTGTTTTGTTTGTTTGTTTTTTGAGACTGACTCTCACTCTGTCGCCCAGGCTGG
AGTGCAGTAGCGCAATCTTGGCTCACTGCAACCTCCACCTCCTGGGTTCAAGCGATTCTCATGCCTCAGCCTCCC
AAGTAGCTGGGATTACAGGCATGTGCCCGCCCACCCAGCTAATTTTTGTATATACATATATATTTTTTGAGACAG
AGTTTCACTCTCTTGTTGCCCAGACTGGAGTGCAATGGCGCAATTTTGGCTCACTGCAACCTCTGCCTCCCGAGT
TCAAGCAGTTTTCCTGCTGGATGACAGAGCGAAACCTTGTCTCTAAAAAAAAAGAAGCTTCTGCTTCCCAAGTTC
TGGTGTAGTGTTAAAGAAGAATACGTCTATTTATCTGAAAAGGCTATGAAAATCCTCTTCCATTTTCCAAATACG
TATCTCTGAGGTCAAGTTTTTTTCATATACCTCAACCAAAGCAACATACTGCAACAGACTCAATGCAGAGGCAGA
TAGGAGAATGCAACTATTTGATTCTAAGCCAAACATTAAAGAGATTTGCAAAAATGTGAAACAGTGCCTTTTTTT
TGAAAATATAGTTATTTTATTAAAAATGTTTATATTACTATGTAATGGGTTATATTAATCATTAATTGTTTTTAT
AATTAATCATTTGAGAAATTTCTGTTTAAATTTCTAGTATGTAAATATCTATAAATATAACTCACATAAACAAAA
GCTCTTTGGAATCTTCAATAAATTTTAAGAGATATAGGGCCTCTGAGACCAAAACATCTGAAACCACTTCCCTGG
TGACCAGTGGCCAGCAGATGAGACTGTGCTGAGGAAGCCGATATGAATTTGATTGCTGGATGGGGAATTCTTGGC
CCAGAGCCCTCTGAGAGGGATGTATGACTGTCCCTAAAAAATCTCTCTTTCATC**AGAATGGACGCGATCTCTCT**
ATCTGGTCCAGTGGCAGCCGGCACATGAAGAAGCAGACATTTGTGGTACATGCAGGGACAGATACAAACGGAGAT
ATCTTTTTCATGGAGGTAGGTGCTGGTTCATGCTGGGGGCCCAAAGGGCTATTGAGAGTCACAGGGAACTCATA
GGACCGATGCCAGGATATTTTTCTCATTTATCTCATTCATCTTCATGATCTATTTATCATTAAATTATCAGATTG
GTTTTTTTGTTTTTTGCGTGTGTGTTTTTTTGAGATAGTGTCTCGCTCTGTCACCCAGGCTGGAGTGTAGTGGCT
TGATCTCGGCTCACTGCAACCTCCGCCTCCCGGGTTCAAGCAGTTCTCCTGCCACAGCCTCCCAAGTAGCTGGGA
TTACAGGCGCCAGCCACCAAGCCCAGGTGATTTTTCTATTTTTAATAGAGATGGGGTTTGATCATGTTGGCCAGG
CTGGTCTCAAACTCCTGACCTCAAGTAATCCACCCACCTTGGCCTTCCAAAATGCTGGTATTATAGGCATGAGCC
ACCGCACCCGGCCAAATTATCAGATTGTTATTCAAATAATTAAATTTTATATAGTAGATGATACCCATTTCTTTT
TTTTTTTTTTTTGAGACAGAGTATCGCTCTGTCACCCAGGCTGGAGTGCAGTGGTGTGATCTTGGCTCACTGCA
GCCTCCACCTCCCAGGTTCAAGCAATTCTCCTGCCTCAGCTTCCTGAGTAGCTAGGTTCGAGACCAGCCTGGCCA
ACATGGTGAAACCCTGTCTCTACTAAAAATACAAAAATTAGCTGAGCATGGTGATGTGTGCCTGTAATCTCAGCT
ACTCGGGAGGCTGAGGCAGGAGAATCATTTAACCCACAAGGCAGAGGTTGCAGTGAGTCGAGATTGCACCACTG
CACTCCAGCCTGGGCAACAGAGTGGGACTCCATCTCAAAAAAAAAAAAAAAAAATTATAGCCAATGTGATGGTGT
GCACCTGTAGTCCCTGCTACTTTGGGGGCTGAGGCAGGAAGAACCTGGCTGGTCGAGGCTGCAGTGAGCTGAGAT
GGCGCCACTGCAGTCAAGCCTGGGGTACAAAGTGAGACCCTGTCTCAAAAAAAAAAAAAAGAGGTAATAAATAAC
TGATTGATTTGTGCCTCCTCCCTTATACATCGGTTCCTTCTAATGACTCCATCTCTTCCAACAATCCTTGTAGCA
GGCAGCATGTCAGTAGTTCTGTTTTGTGTCTTCACCTAGGGAAACCATGAGCGTAAGGCAGAAAACCTGACCATG
GGGATGCCCCAGGACCACCTTTCCCTGTAGGAAACAGTCTAGGGAGAGGCTGTCAGAGACAGGAACCACTGAGCC
GCTTTGTGCACTTCTCC**AGGTGTGCGATGACTGTGTGGTGTTGCGTAGTAACATCGGAACAGTGTATGAGCGCT**
GGTGGTACGAGAAGCTCATCAACATGACCTACTGTCCCAAGACGAAGGTGTTGTGCTTGTGGCGTAGAAATGGCT
CTGAGACCCAGCTCAACAAGTTCTATACTAAAAAGGTACGCAGGATCTGTGTTTGGGTTGGGGCTAG

Final T>G-construct insert sequence:

CCTTGTTAAGAGTCATGTGTTGGCTTTTC**AGGTAAATAAGAATGACATCCGCAAGAAGGTGAGGCGCCTAATGG**
GAAAGTCGCACATTGGGCTTGTGTACAGCCAGCAAATCAATGAGGTGCTTGATCAGCTGGCGAACCTGGTAAGC
ACGTCTGGCCACCCCTTAGGCTTCCCCATGGGTCATTTCTTGGTTTGTGTCACTTGCAGTCCAGTTCACCCCGTT
TTTGAAAATGGAGCAGTTGTCTTTGACTGTAAATGAGGCATTAGTCCCTGTGTTCTGTGATGGGATTCTCTGTAT
AAAACCATCGTAGTGCATCAGTTTTGATGTGTGGTCTGTGAACTGCTGAGGGTTCAGAAGGTGAAAACGATTTCA
AATGATTCTAAAATGTTATTTGCTGTTTTCACGGGGTTACATTTGCACTGATGGTACCAACGCAGTGATGGGTAA
AACTGCTGGCACTGTAACCCAAGTGAAGACAATGGCCCCAAACTATGCCAGCAGCCATTGCATTCCTTGCCTTCA

TATACTCTCAGTTTTTCGTTTTTGTTTTGTTTGTTTGTTTTTTGAGACTGACTCTCACTCTGTCGCCCAGGCTGG
AGTGCAGTAGCGCAATCTTGGCTCACTGCAACCTCCACCTCCTGGGTTCAAGCGATTCTCATGCCTCAGCCTCCC
AAGTAGCTGGGATTACAGGCATGTGCCCGCCCACCCAGCTAATTTTTGTATATACATATATATTTTTTGAGACAG
AGTTTCACTCTCTTGTTGCCCAGACTGGAGTGCAATGGCGCAATTTTGGCTCACTGCAACCTCTGCCTCCCGAGT
TCAAGCAGTTTTCCTGCTGGATGACAGAGCGAAACCTTGTCTCTAAAAAAAAAGAAGCTTCTGCTTCCCAAGTTC
TGGTGTAGTGTTAAAGAAGAATACGTCTATTTATCTGAAAAGGCTATGAAAATCCTCTTCCATTTTCCAAATACG
TATCTCTGAGGTCAAGTTTTTTTCATATACCTCAACCAAAGCAACATACTGCAACAGACTCAATGCAGAGGCAGA
TAGGAGAATGCAACTATTTGATTCTAAGCCAAACATTAAAGAGATTTGCAAAAATGTGAAACAGTGCCTTTTTTT
TGAAAATATAGTTATTTTATTAAAAATGTTTATATTACTATGTAATGGGTTATATTAATCATTAATTGTTTTTAT
AATTAATCATTTGAGAAATTTCTGTTTAAATTTCTAGTATGTAAATATCTATAAATATAACTCACATAAACAAAA
GCTCTTTGGAATCTTCAATAAATTTTAAGAGATATAGGGCCTCTGAGACCAAAACATCTGAAACCACTTCCCTGG
TGACCAGTGGCCAGCAGATGAGACTGTGCTGAGGAAGCCGATATGAATTTGATTGCTGGATGGGGAATTCTTGGC
CCAGAGCCCTCTGAGAGGGATGTATGACTGTCCCTAAAAAATCTCTCTTTCATC**AGAATGGACGCGATCTCTCT**
ATCTGGTCCAGTGGCAGCCGGCACATGAAGAAGCAGACATTTGTGGTACATGCAGGGACAGATACAAACGGAGAT
ATCTTTTTCATGGAGGGAGGTGCTGGTTCATGCTGGGGGCCCAAAGGGCTATTGAGAGTCACAGGGAACTCATA
GGACCGATGCCAGGATATTTTTCTCATTTATCTCATTCATCTTCATGATCTATTTATCATTAAATTATCAGATTG
GTTTTTTTGTTTTTTGCGTGTGTGTTTTTTTGAGATAGTGTCTCGCTCTGTCACCCAGGCTGGAGTGTAGTGGCT
TGATCTCGGCTCACTGCAACCTCCGCCTCCCGGGTTCAAGCAGTTCTCCTGCCACAGCCTCCCAAGTAGCTGGGA
TTACAGGCGCCAGCCACCAAGCCCAGGTGATTTTTCTATTTTTAATAGAGATGGGGTTTGATCATGTTGGCCAGG
CTGGTCTCAAACTCCTGACCTCAAGTAATCCACCCACCTTGGCCTTCCAAAATGCTGGTATTATAGGCATGAGCC
ACCGCACCCGGCCAAATTATCAGATTGTTATTCAAATAATTAAATTTTATATAGTAGATGATACCCATTTCTTTT
TTTTTTTTTTTTTGAGACAGAGTATCGCTCTGTCACCCAGGCTGGAGTGCAGTGGTGTGATCTTGGCTCACTGCA
GCCTCCACCTCCCAGGTTCAAGCAATTCTCCTGCCTCAGCTTCCTGAGTAGCTAGGTTCGAGACCAGCCTGGCCA
ACATGGTGAAACCCTGTCTCTACTAAAAATACAAAAATTAGCTGAGCATGGTGATGTGTGCCTGTAATCTCAGCT
ACTCGGGAGGCTGAGGCAGGAGAATCATTTTAACCCACAAGGCAGAGGTTGCAGTGAGTCGAGATTGCACCACTG
CACTCCAGCCTGGGCAACAGAGTGGGACTCCATCTCAAAAAAAAAAAAAAAAAAATTATAGCCAATGTGATGGTGT
GCACCTGTAGTCCCTGCTACTTTGGGGGCTGAGGCAGGAAGAACCTGGCTGGTCGAGGCTGCAGTGAGCTGAGAT
GGCGCCACTGCAGTCAAGCCTGGGGTACAAAGTGAGACCCTGTCTCAAAAAAAAAAAAAAAGAGGTAATAAATAAC
TGATTGATTTGTGCCTCCTCCCTTATACATCGGTTCCTTCTAATGACTCCATCTCTTCCAACAATCCTTGTAGCA
GGCAGCATGTCAGTAGTTCTGTTTTGTGTCTTCACCTAGGGAAACCATGAGCGTAAGGCAGAAAACCTGACCATG
GGGATGCCCCAGGACCACCTTTCCCTGTAGGAAACAGTCTAGGGAGAGGCTGTCAGAGACAGGAACCACTGAGCC
GCTTTGTGCACTTCTCC**AGGTGTGCGATGACTGTGTGGTGTTGCGTAGTAACATCGGAACAGTGTATGAGCGCT**
GGTGGTACGAGAAGCTCATCAACATGACCTACTGTCCCAAGACGAAGGTGTTGTGCTTGTGGCGTAGAAATGGCT
CTGAGACCCAGCTCAACAAGTTCTATACTAAAAAGGTACGCAGGATCTGTGTTTGGGTTGGGGCTAG

Insert sequences were synthetized and cloned to multiple cloning site of pET01 vector (MoBiTec GmbH) at GenScript Biotech BV (Netherlands). 1.5E5 HEK293 cells were seeded in 24-well plates. The next day, cells were transfected with 350 ng of T or T>G pET01 constructs or GFP-control using Lipofectamine 3000 (Invitrogen) according to manufacturer's instructions. After 48h cells were harvested in PBS and total RNA was extracted with NucleoSpin RNA Plus kit (Macherey-Nagel). 3-4 μg of RNA was converted to cDNA by using SuperScript™ III First-Strand Synthesis System (ThermoFisher Scientific) and pET01-specific oligo 5'-GATCCACGATGC-3' according to manufacturer's instructions. The presence of exon 30 was assessed by PCR with pET01-specific primers Fw: 5'-GATGGATCCGCTTCCTGCCCC-3' and Rv: 5'-CTCCCGGGCCACCTCCAGTGCC-3' using Phusion Hot-start II DNA polymerase (Thermo Scientific) followed by Sanger Sequencing. The two bands oberved in PCR product from cells transfected with the T>G construct (Figure S1B) were separately gel purified using NucleoSpin Gel and PCR Clean-up, Mini kit (Macherey-Nagel).

Differentiation of SC-islets

Details of the protocol are described in STAR protocols of ref (2).

RAB3A Activation assay

1. PCR to generate and amplify gRNA guides for generating *dex30* HEK293 cells

Linear DNA templates for expression of guide RNAs (gRNAs) were prepared as previously described (3). Shortly, gRNA templates with 19 bp overhangs on 5′ and 3′ ends were concatenated to U6 promoter and terminator sequences (tracr) with matching overhangs using PCR with Phusion polymerase (ThermoFisher Scientific). Concatenation PCR components, overlapping sequences marked in capitals:

1. gRNA, where n's represent the specific guide sequence:
   5'-GTGGAAAGGACGAAACACCgnnnnnnnnnnnnnnnnnnnGTTTTAGAGCTAGAAATAG -3'
2. U6 promoter:
   5'-GTAAAACGACGGCCAGTgagggcctatttcccatgattccttcatatttgcatatacgatacaaggctgttagagagataattggaattaatttgactgtaaacacaaagatattagtacaaaatacgtgacgtagaaagtaataatttcttgggtagtttgcagttttaaaattatgttttaaaatggactatcatatgcttaccgtaacttgaaagtatttcgatttcttggctttatatatcttGTGGAAAGGACGAAACACC-3'
3. Terminator:
   5'-GTTTTAGAGCTAGAAATAGcaagttaaaataaggctagtccgttatcaacttgaaaaagtggcaccgagtcggtgcTTTTTTTCATGGTCATAGCTGTTTCCT
4. Primer Fw: actgaattcggatcctcgagcgtctcaccctGTAAAACGACGGCCAGT
5. Primer Rv: catgcggccgcgtcgacagatctcgtctcacatgAGGAAACAGCTATGACCATG

5 ng of U6 promoter and U6 terminator were concatenated with 2 µM of gRNA template in a PCR reaction with Phusion polymerase (Thermo Fisher Scientific) in the presence of 1x High Fidelity buffer, 50 pmol of forward and reverse primers and 200 µM dNTP in a volume of 100 µl. The PCR cycling conditions were as follows: 35 cycles of 98°C for 10 s, 52°C for 30 s and 72°C for 12 s. The PCR amplified guides were subjected to agarose gel electrophoresis and purified from the gel using NucleoSpin Gel and PCR Clean-up, Mini kit (Macherey-Nagel).

2. RAB3A expression vector

The pEGFP–C1A-RAB3A construct (4) was a kind gift from Dr. Johan Peränen, Institute of Biotechnology, University of Helsinki, Helsinki, Finland.

Supplemental References

1. Schneeberger PE, Kortum F, Korenke GC, Alawi M, Santer R, Woidy M, et al. Biallelic MADD variants cause a phenotypic spectrum ranging from developmental delay to a multisystem disorder. *Brain.* 2020;143(8):2437-53.
2. Barsby T, Ibrahim H, Lithovius V, Montaser H, Balboa D, Vähäkangas E, et al. Differentiating functional human islet-like aggregates from pluripotent stem cells. *STAR Protoc.* 2022;3(4):101711.
3. Balboa D, Weltner J, Eurola S, Trokovic R, Wartiovaara K, and Otonkoski T. Conditionally Stabilized dCas9 Activator for Controlling Gene Expression in Human Cell Reprogramming and Differentiation. *Stem Cell Reports.* 2015;5(3):448-59.
4. Lonka-Nevalaita L, Lume M, Leppanen S, Jokitalo E, Peranen J, and Saarma M. Characterization of the intracellular localization, processing, and secretion of two glial cell line-derived neurotrophic factor splice isoforms. *J Neurosci.* 2010;30(34):11403-13.
