# Supplementary material for: A splice site variant in MADD affects hormone expression in pancreatic β cells and pituitary gonadotropes
Source: JCI Insight. 2024 May 22;9(10):e167598. doi: 10.1172/jci.insight.167598 (PMC11141940; doi:10.1172/jci.insight.167598)

Full unedited gel for Figure 1D, RT-PCR with primers binding around *MADD* exon 30

Lanes corresponding to the cropped image

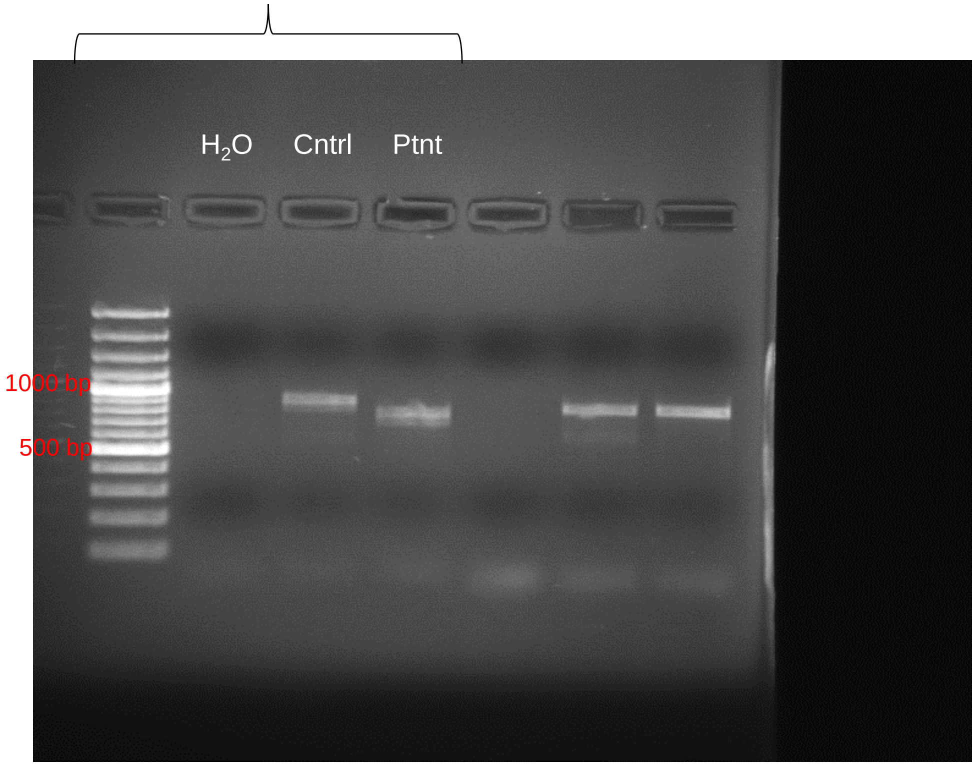

Full unedited gel for Figure 2A, RT-PCR with primers specific to *MADD* and *GAPDH*

Lanes corresponding to the cropped and flipped image

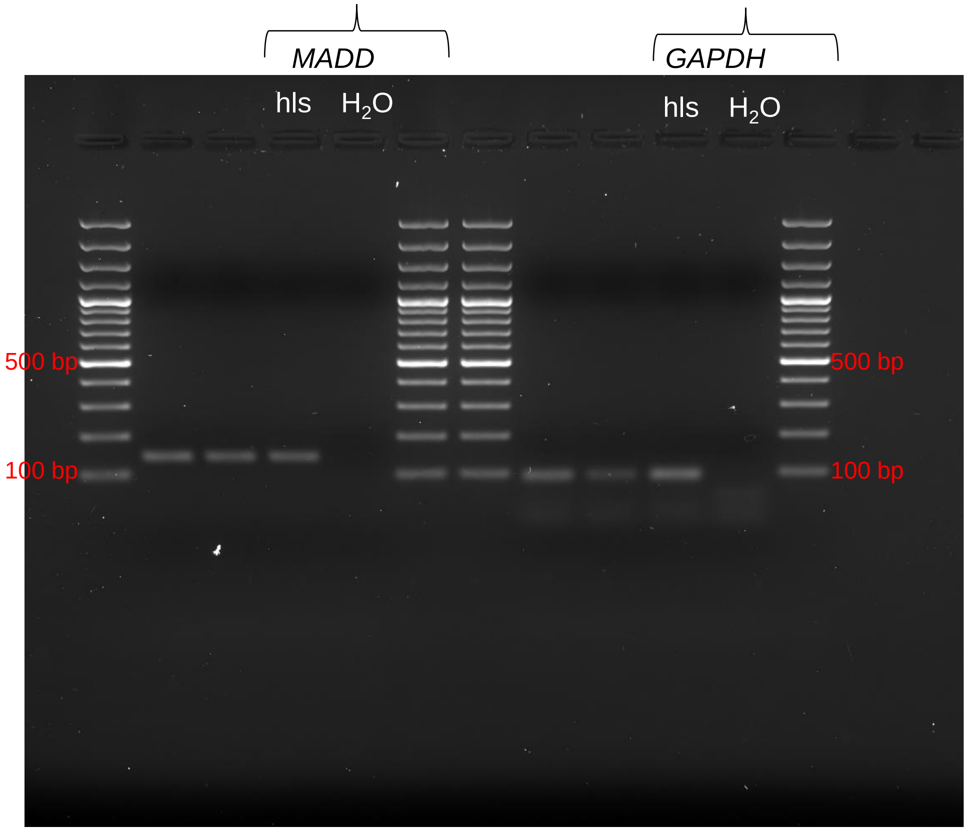

Lanes from the same gel were used for Figure S5A!

Full unedited gel for Figure 2C, PCR with primers binding to genomic DNA around *MADD* exon 30

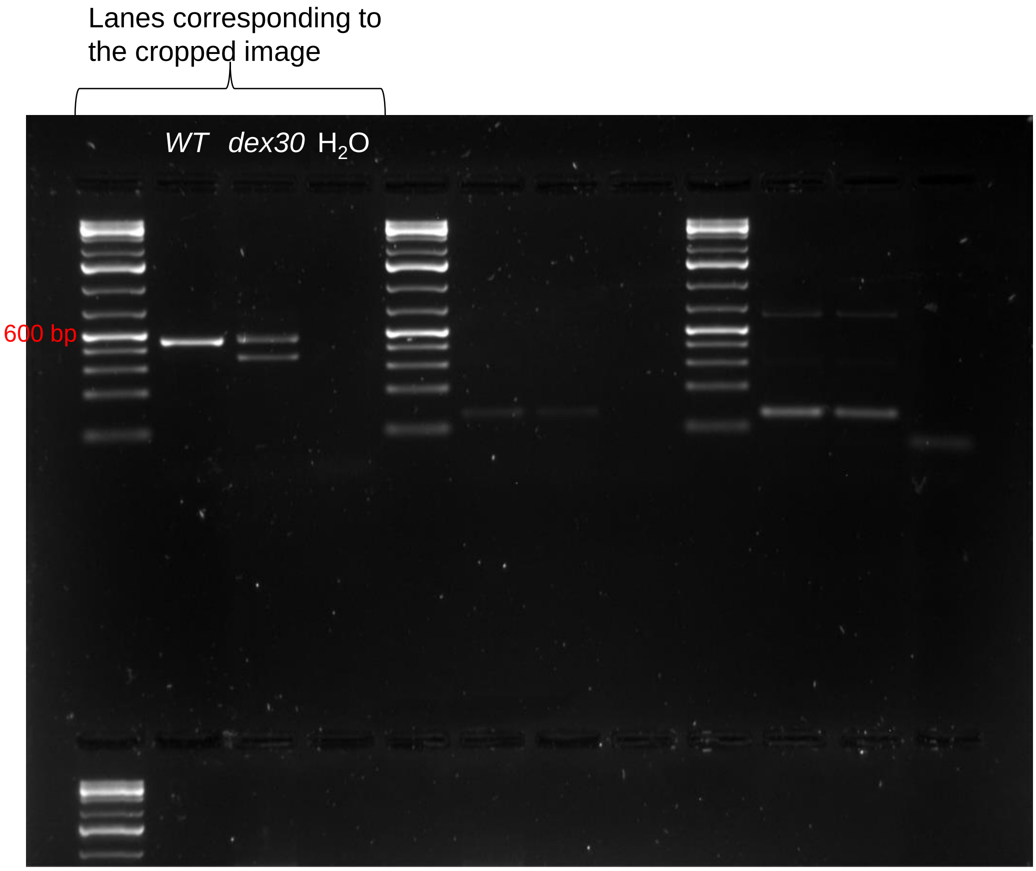

Full unedited gel for Figure 5E, RT-PCR with primers binding around *Madd* exon 30

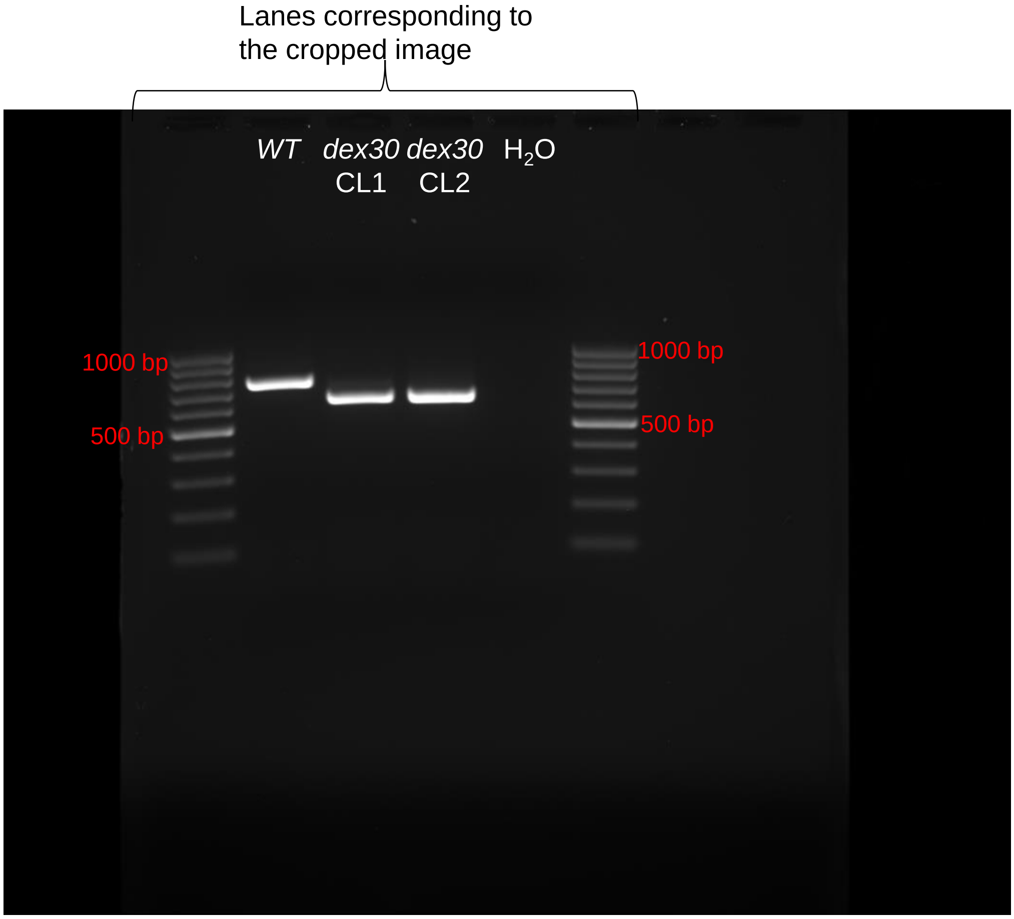

Full unedited gel for Figure S1B, minigene assay PCR

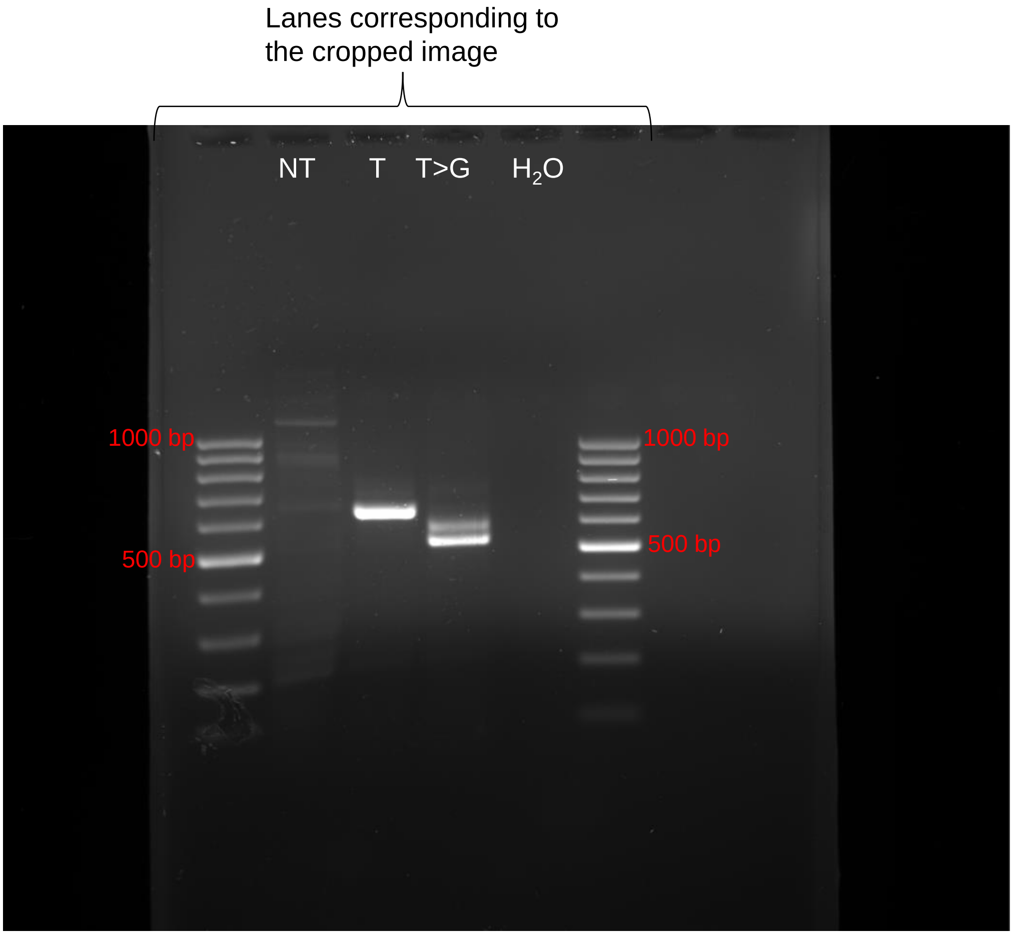

Full unedited gel for Figure S4A, RT-PCR with primers binding around *MADD* exon 30 (see also next page (4))

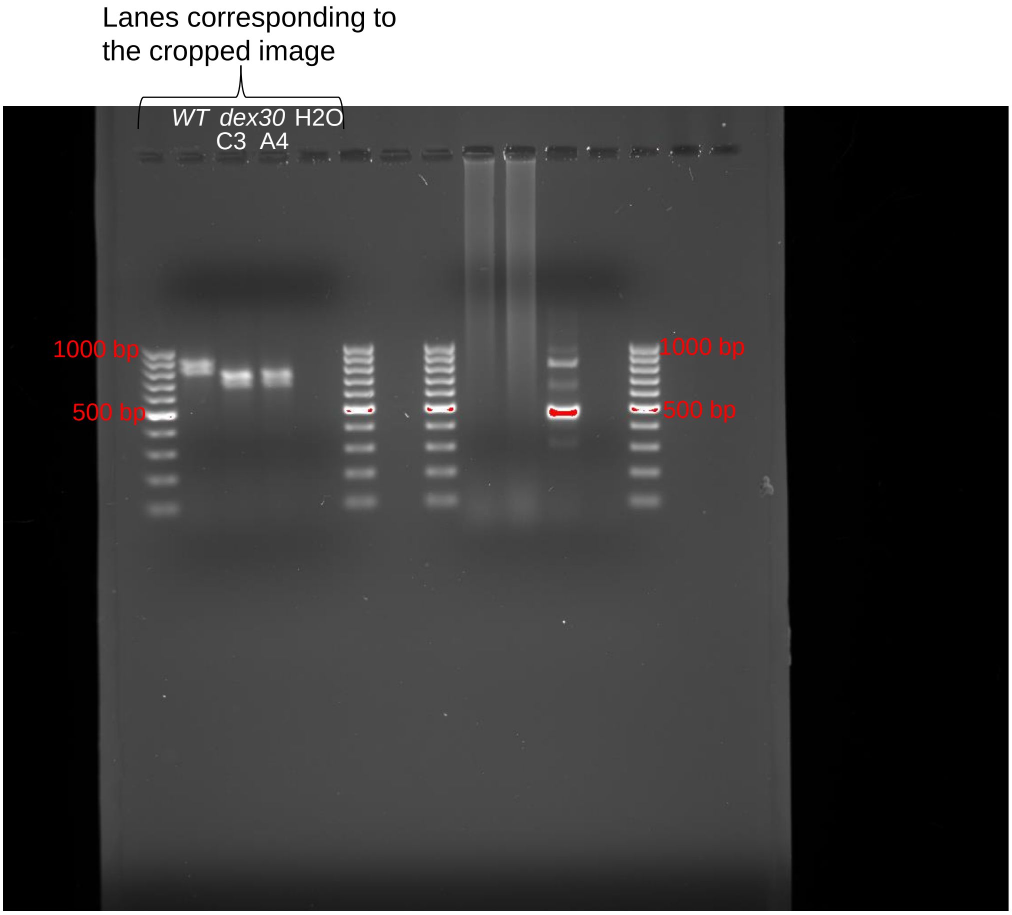

For the final Figure S4A the gel was run a little longer and this image with different frame was taken to improve resolution and focus.

Lanes corresponding to the cropped image

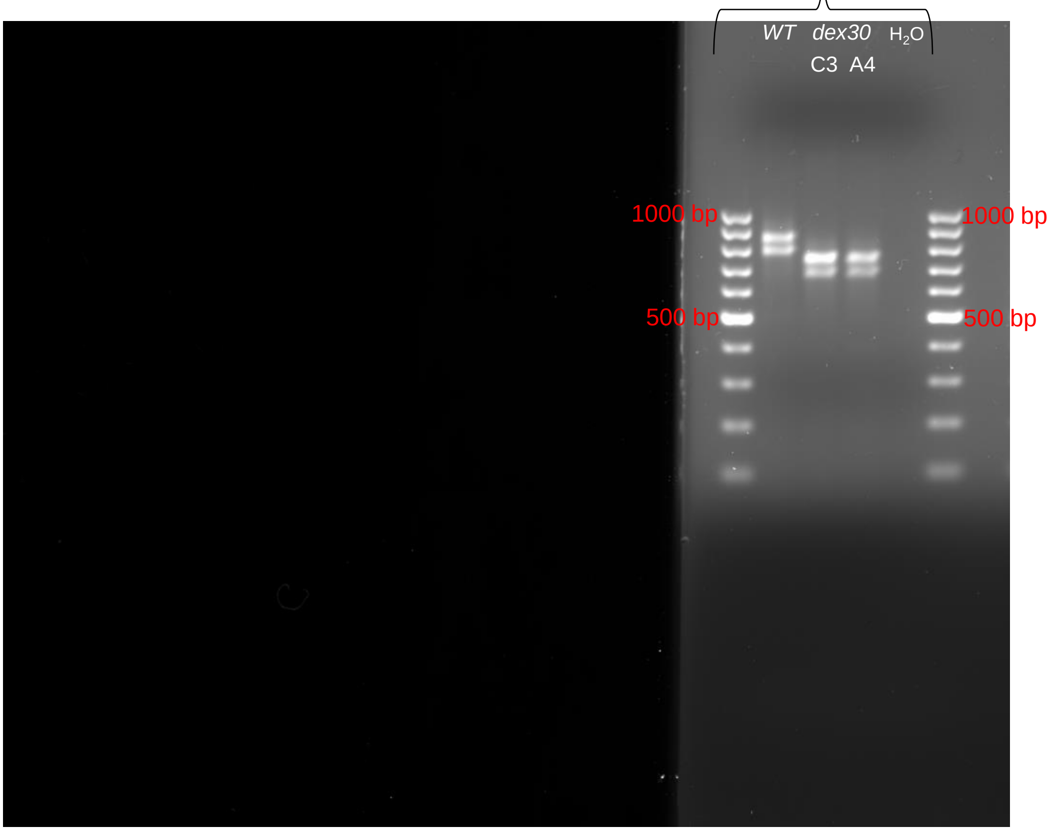

Full unedited gel for Figure S5A, RT-PCR with primers specific to *MADD* and *GAPDH*

Lanes corresponding to the cropped image

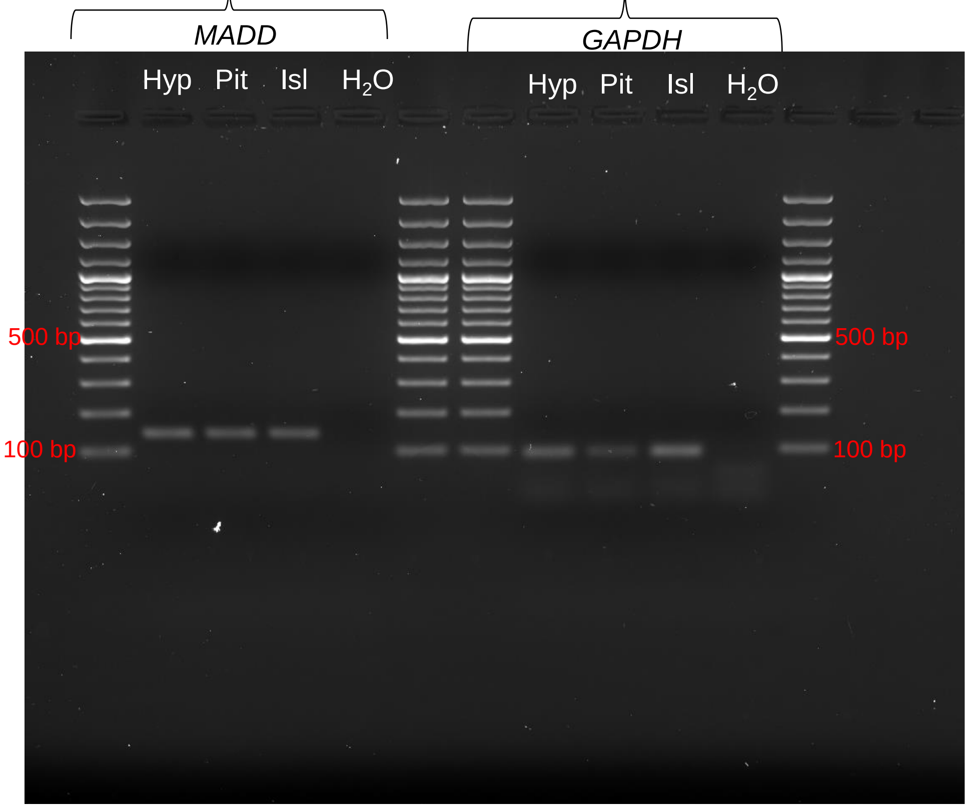

Lanes from the same gel were used for Figure 2A!

Full unedited blot for Figure S6D, antibody against MADD

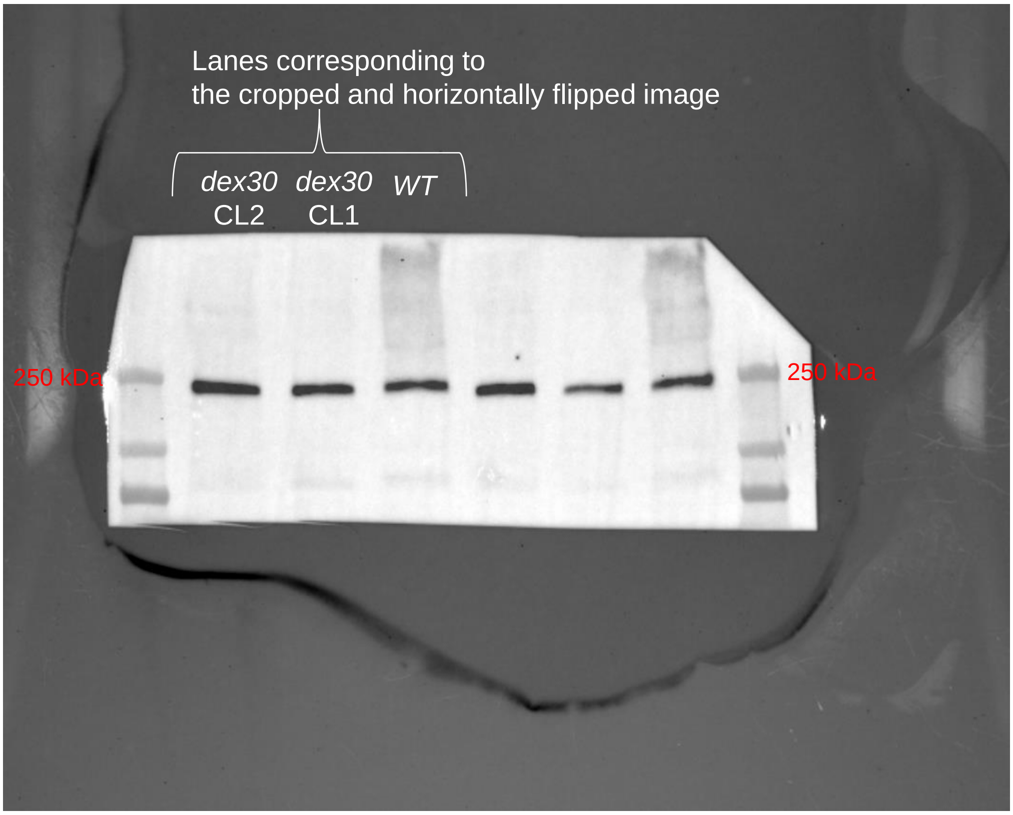

Full unedited blot for Figure S6D, antibody against beta-Actin

Lanes corresponding to
the cropped and horizontally flipped image

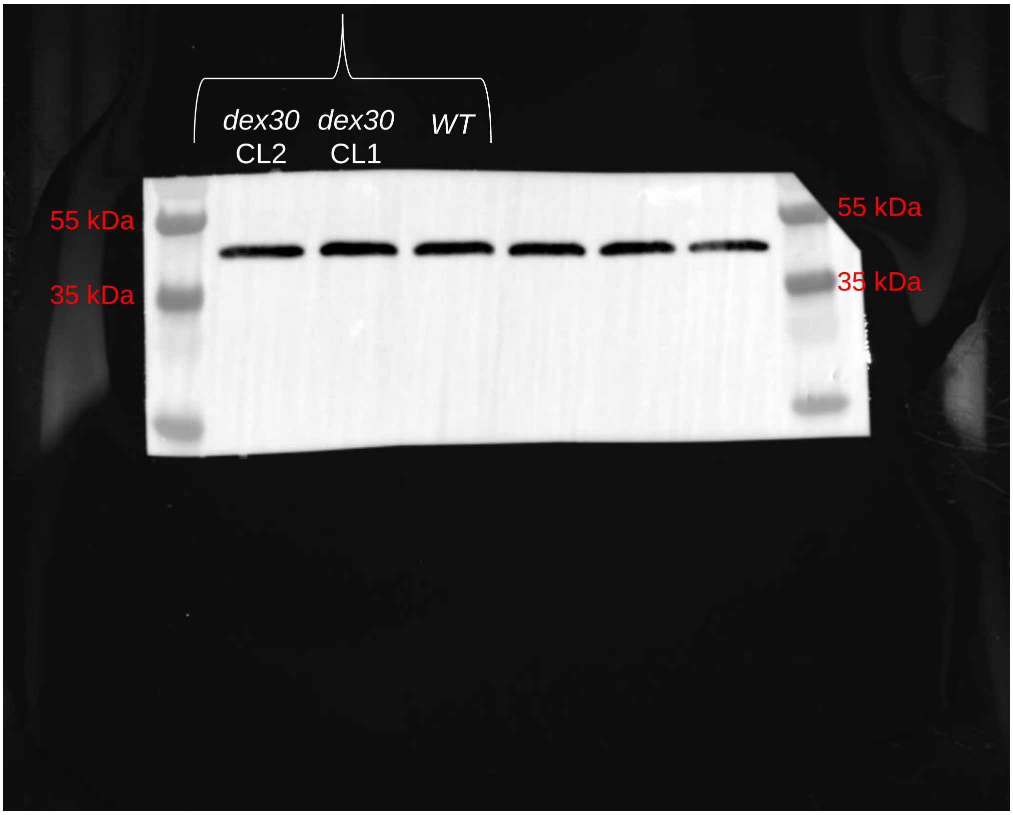

Full unedited blot for Figure S6I, antibody against phospho-ERK1/2

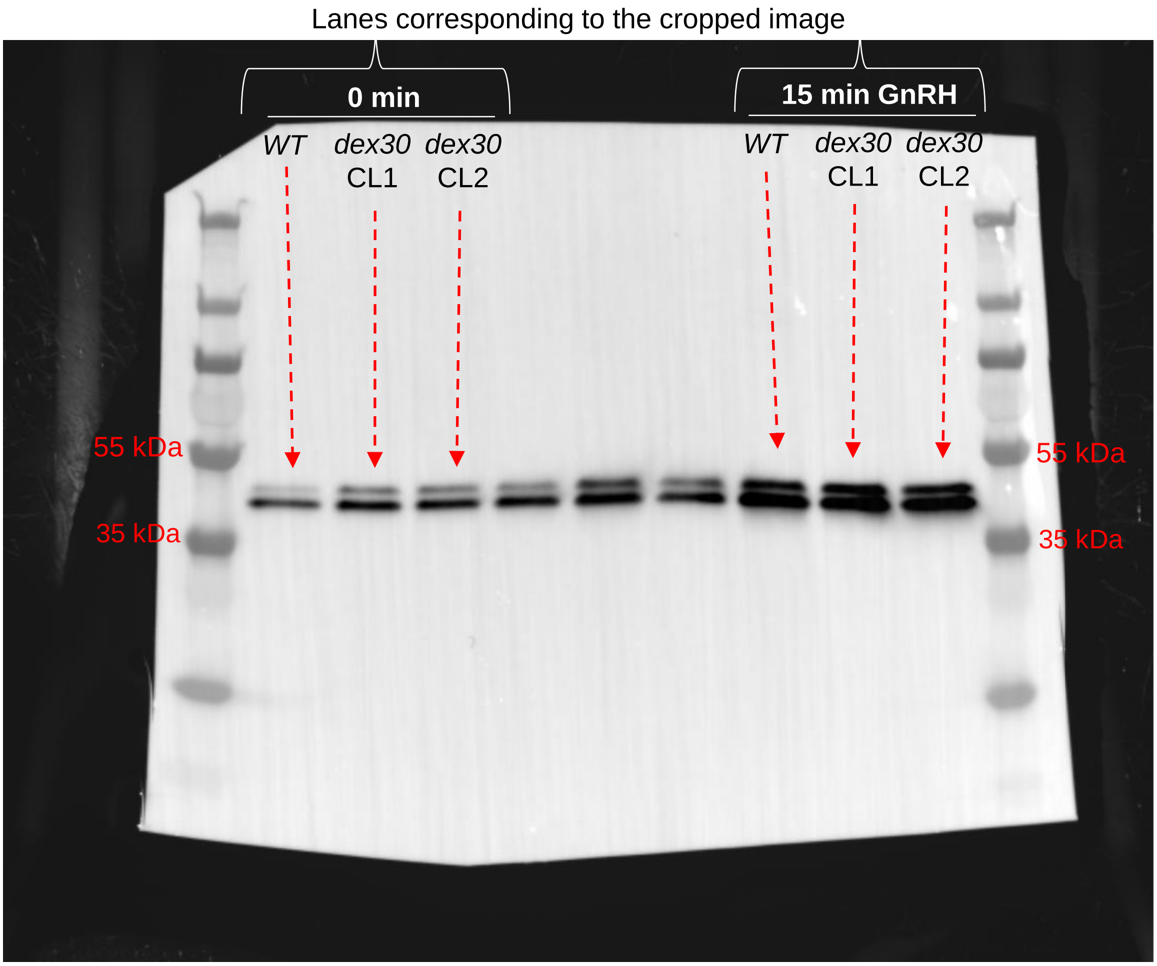

Full unedited blot for Figure S6I, antibody against total-ERK1/2

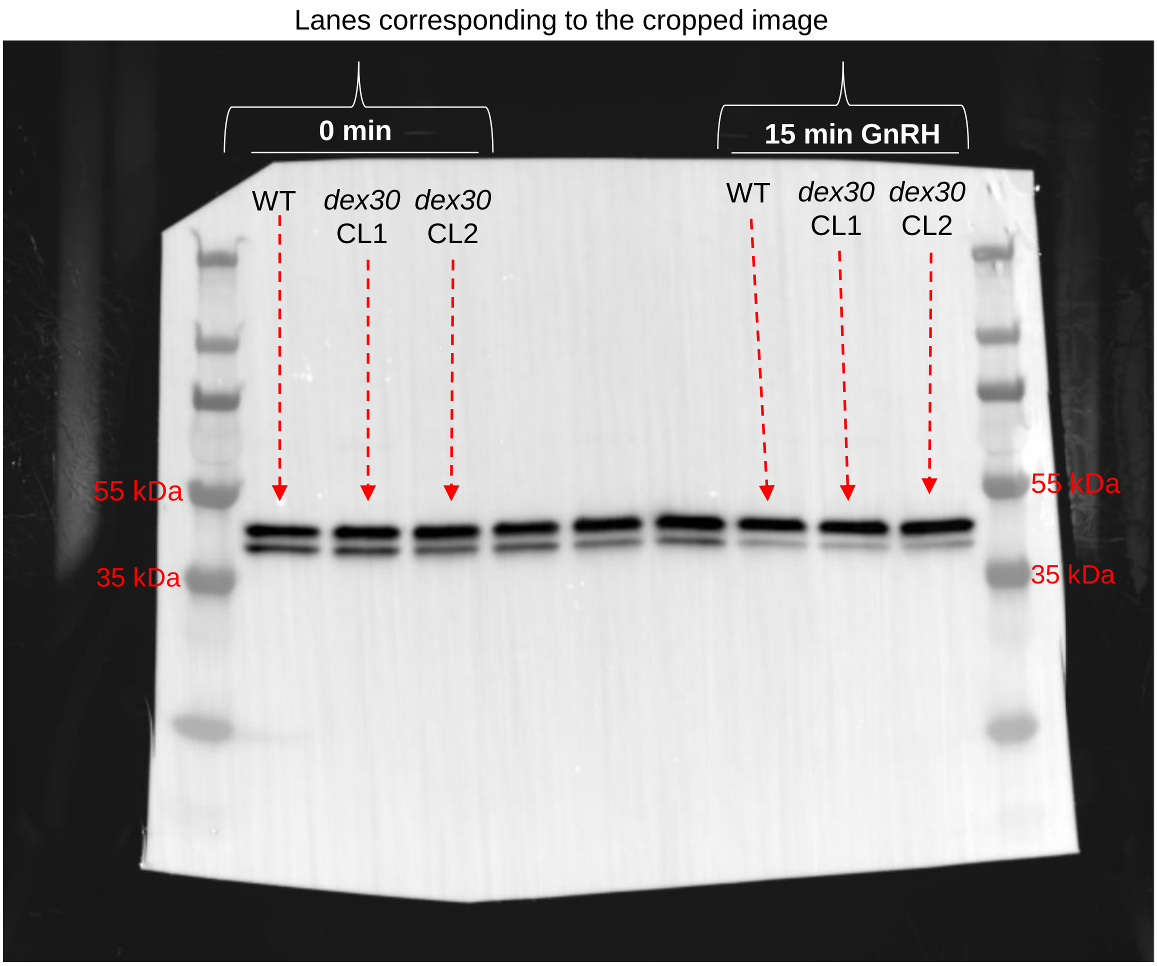

Full unedited blot for Figure S7A, antibody against RAB3A-D

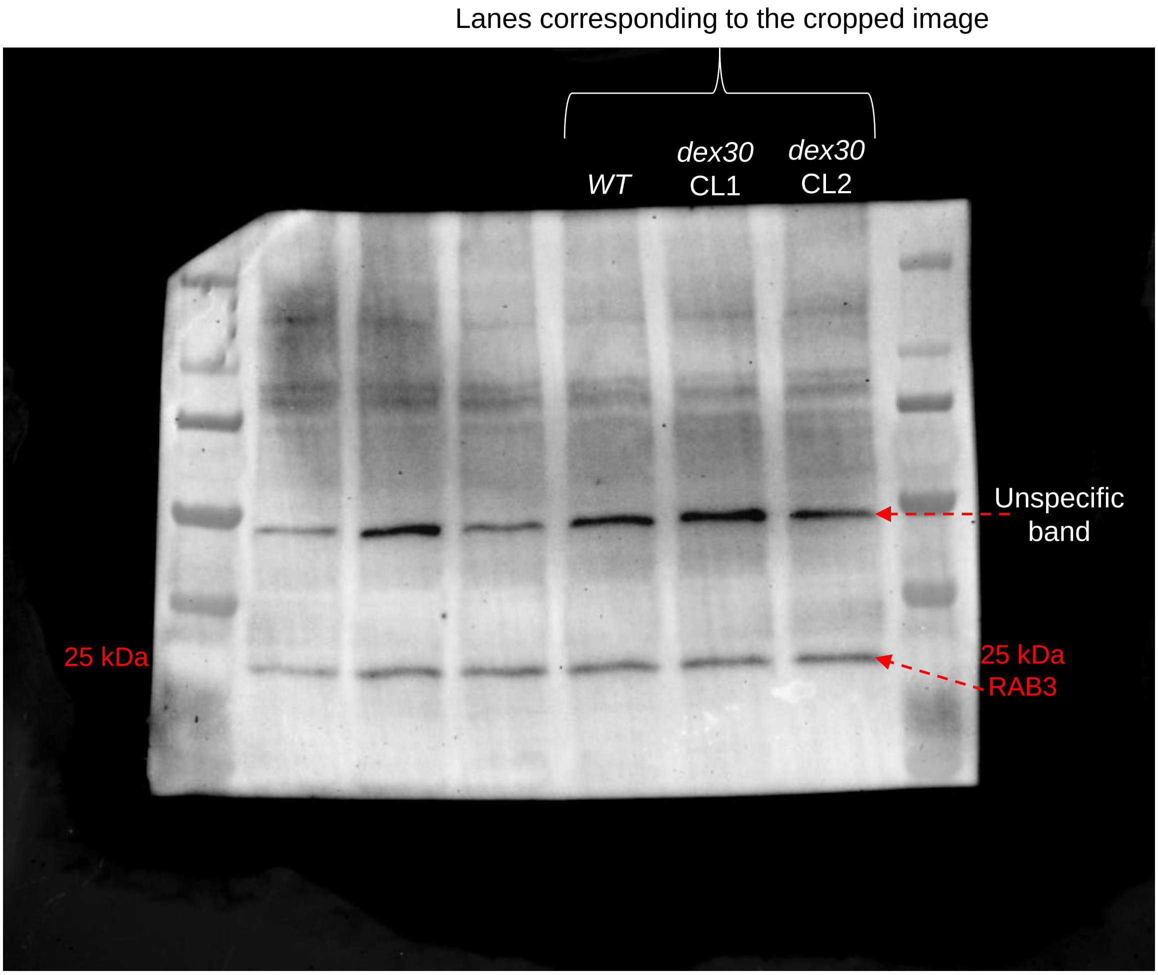

Full unedited blot for Figure S7A, antibody against beta-Actin

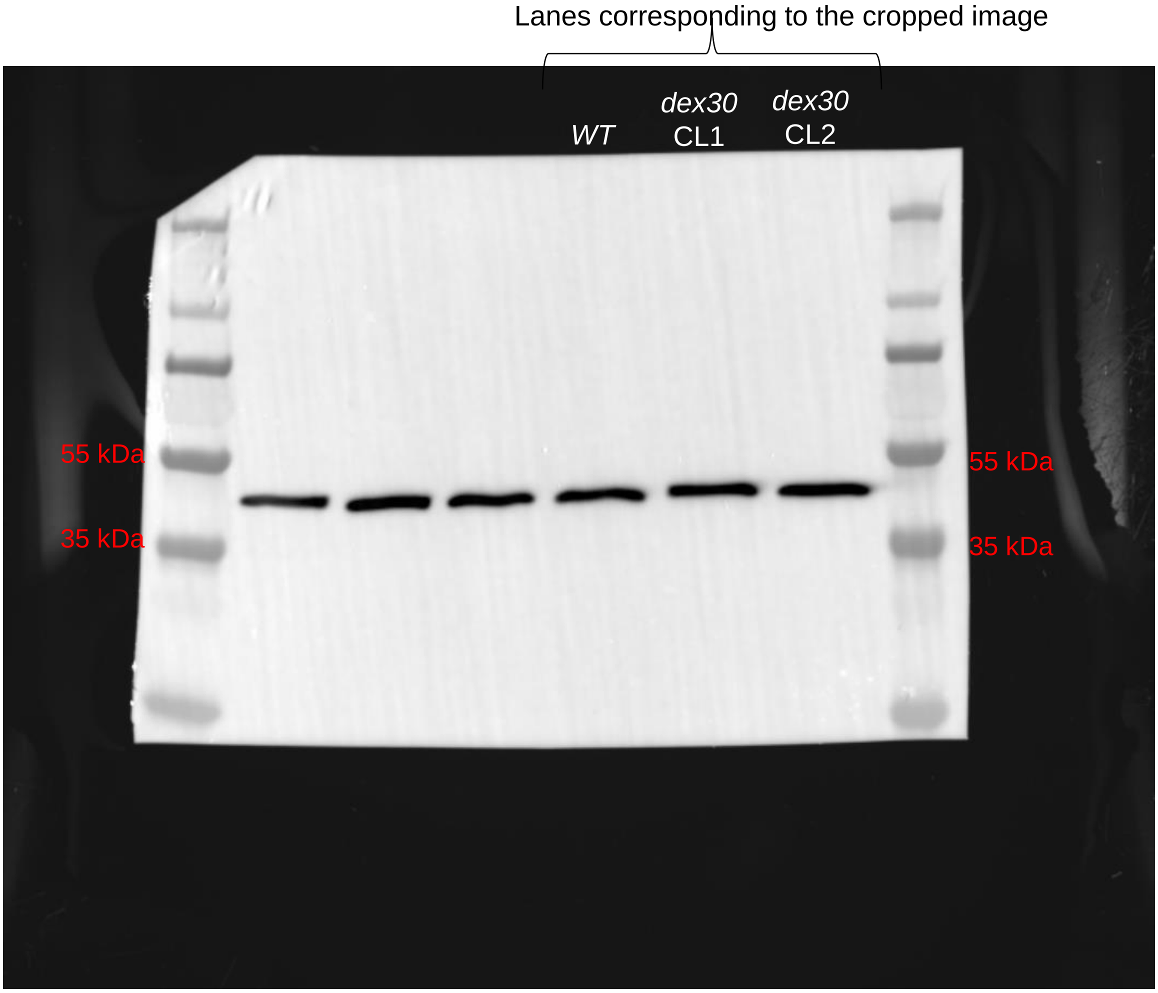

Full unedited gel for Figure S7C, RT-PCR with primers binding around *MADD* exon 30

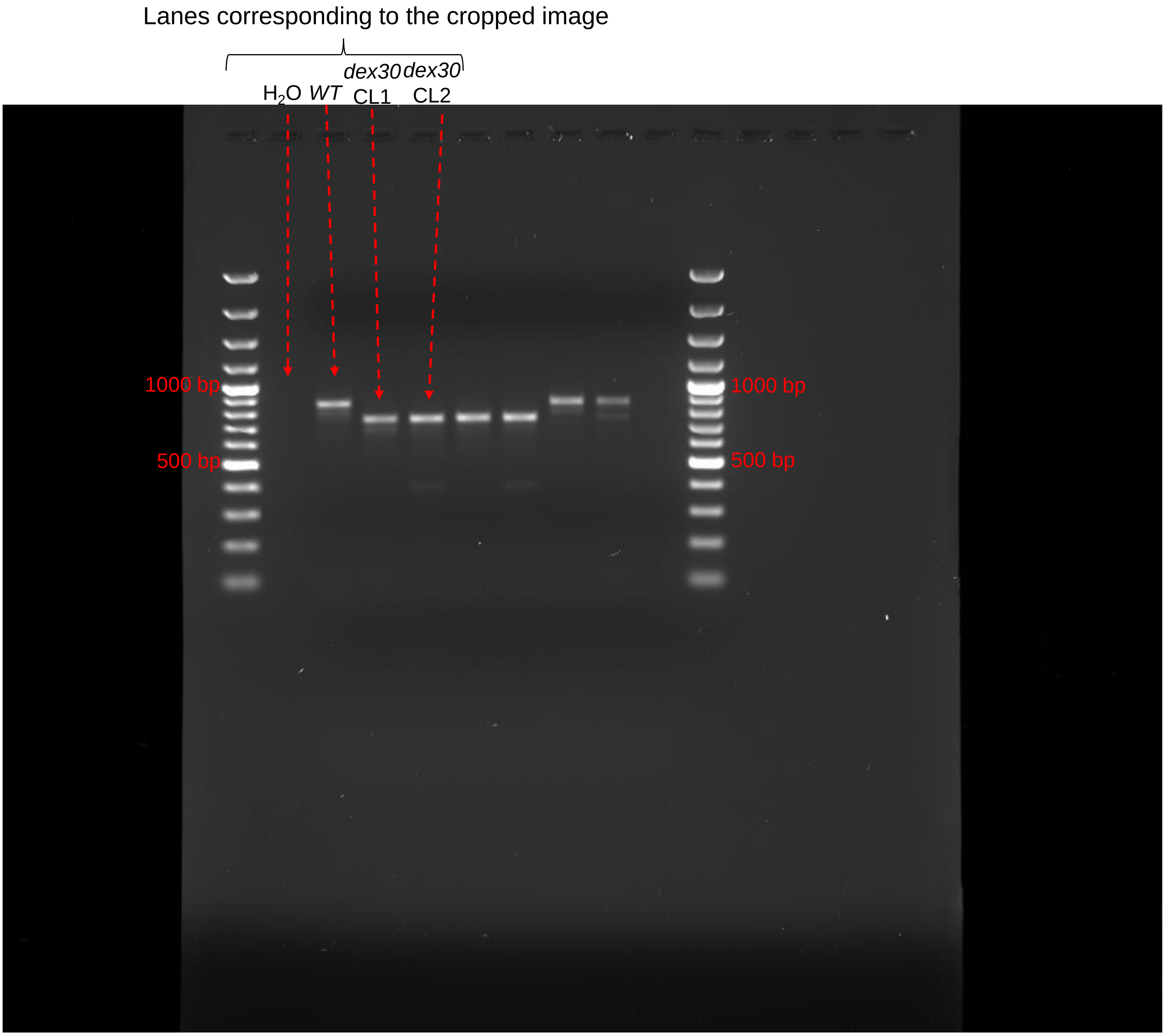

Full unedited blot for Figure S7D, antibody against RAB3A, *WT* and *dex30* CL1

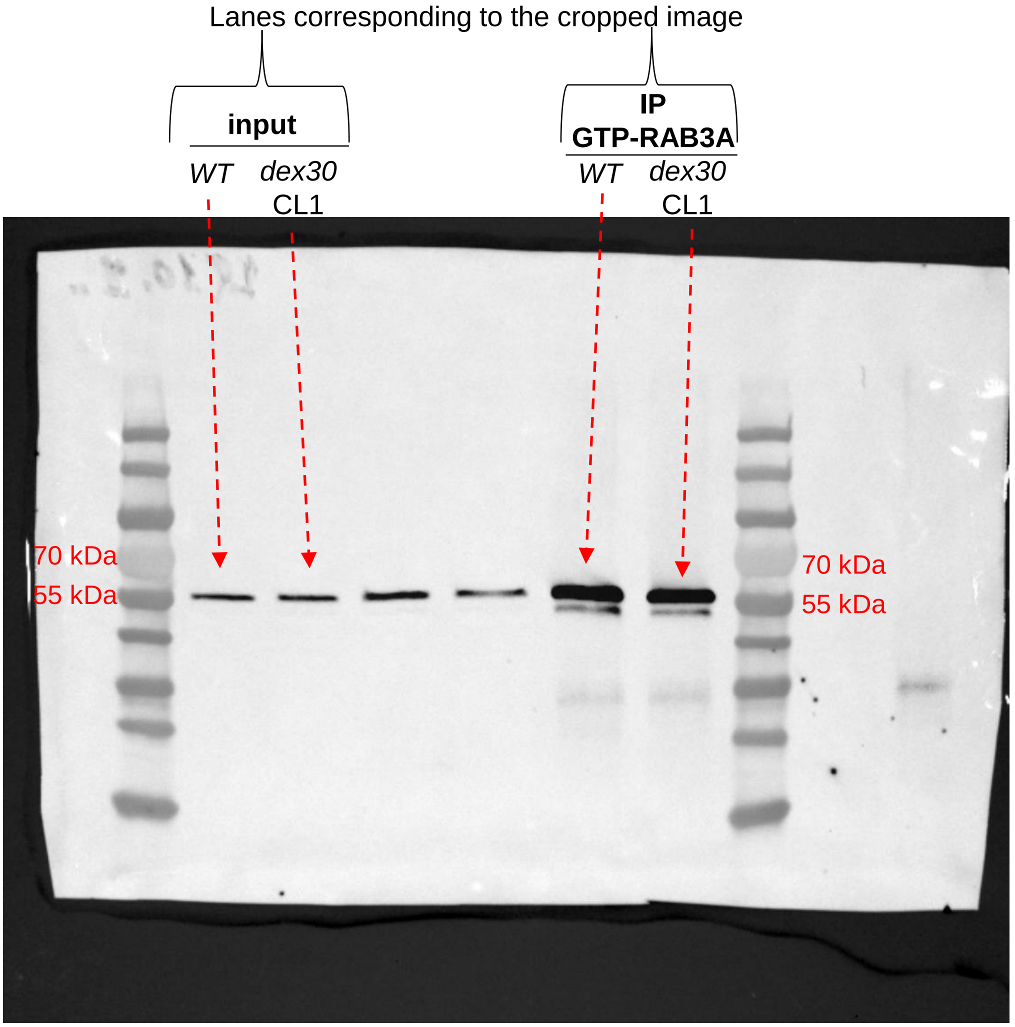

Full unedited blot for Figure S7D, antibody against RAB3A, *dex30* CL2

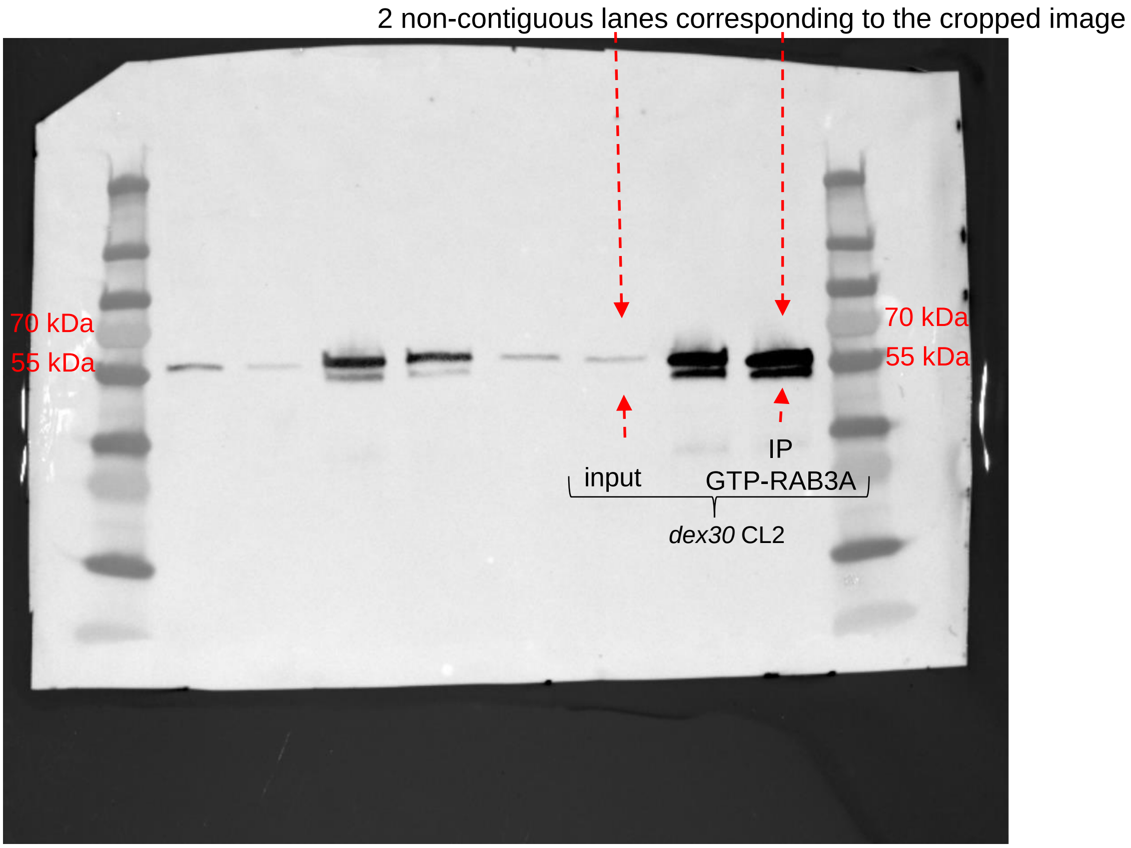

Supplement: Unedited blot and gel images [file jciinsight-9-167598-s175.pdf]
